# Supplementary material for: Combining demographic shifts with age-based resistance prevalence to estimate future antimicrobial resistance burden in Europe and implications for targets: A modelling study
Source: PLoS Med. 2025 Nov 4;22(11):e1004579. doi: 10.1371/journal.pmed.1004579 (PMC12585039; doi:10.1371/journal.pmed.1004579)
Supplement: S1 Appendix — Fig A2: Incidence of BSIs to 2030, summed across countries. A) by bacterial species. Acineto. stands for Acinetobacter. “projection” refers to population projection of either “BSL” or “LMRT” (defined differently for the UK, see Methods) B) by 15-year age-band, summed across all 8 species. Acronyms are female (f) and male (m). Table A1: Summary of difference in numbers of resistant BSI between “base” and “agesex” models. Total.diff = the total difference in resistant BSI numbers in 2030 between models. Female.diff and male.diff are the equivalent summaries split by sex. top.ages.female and top.ages.male are the age groups with the biggest differences between models for females and males respectively. (PDF) [file pmed.1004579.s001.pdf]

S1 Appendix: Sensitivity analysis and additional results

Table of Contents

Population pyramids 2
Incidence by demographics 3
Summary of “base” vs “agesex” models 4

Population pyramids

Figure A1 shows the population pyramids for 2030 and 2050 respectively, indicating an older age distribution in the “LMRT” scenario.

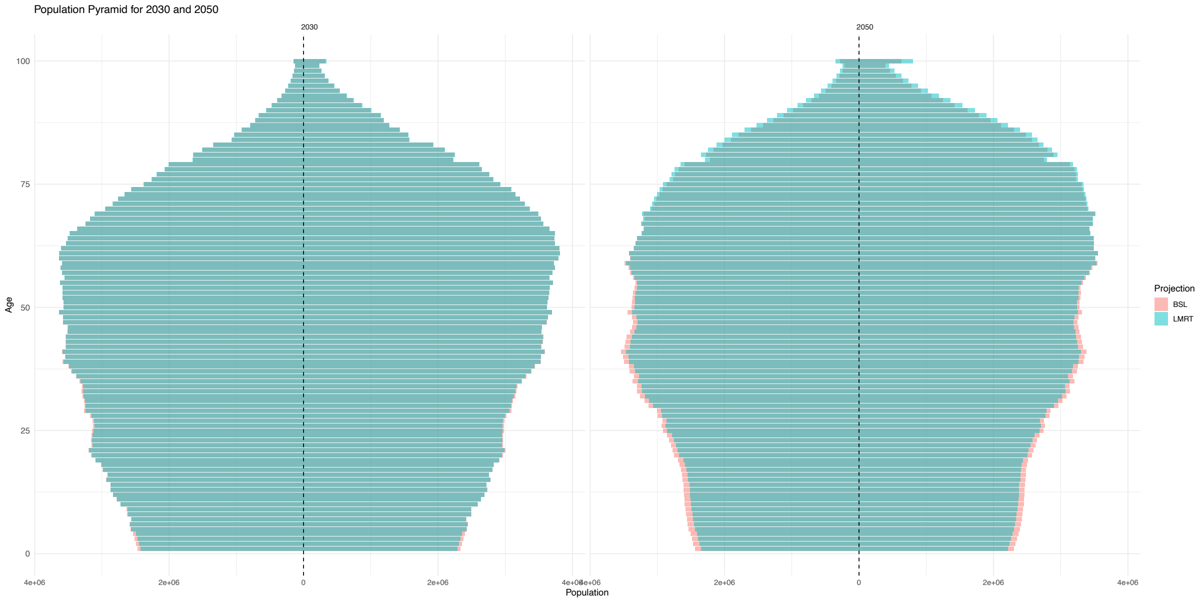

Figure A1: Population distribution for BSL and LMRT scenarios in 2030 and 2050

Incidence by demographics

We compared “BSL” population projections with “LMRT” population projections (see methods) and saw no real difference in the population projections (Figure A2).

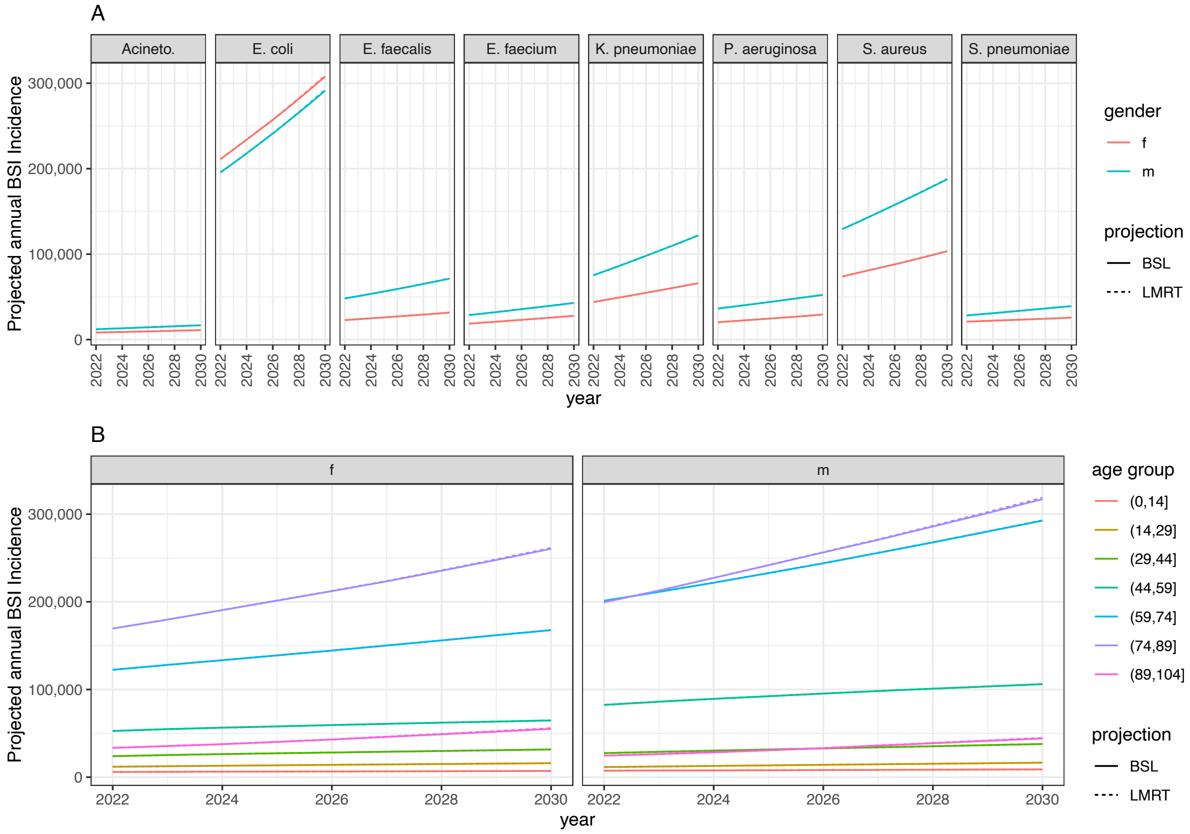

Figure A2: Incidence of BSIs to 2030, summed across countries. A) by bacterial species. Acineto. stands for Acinetobacter. “projection” refers to population projection of either “BSL” or “LMRT” (defined differently for the UK, see methods) B) by 15-year age-band, summed across all 8 species. Acronyms are female (f) and male (m)

Summary of “base” vs “agesex” models

Table A1: Summary of difference in numbers of resistant BSI between “base” and “agesex” models. Total.diff = the total difference in resistant BSI numbers in 2030 between models. Female.diff and male.diff are the equivalent summaries split by sex. top.ages.female and top.ages.male are the age groups with the biggest differences between models for females and males respectively.

Bacteria	Antibiotic	total.diff	female.diff	male.diff	top.ages.female	top.ages.male
Acinetobacter species	multi resistance (acispp)	-70 (-2416 - 2255)	-196 (-1234 - 815)	125 (-1181 - 1439)	91-100(neg) 81-90(neg)	51-60(pos) 61-70(pos)
Acinetobacter species	Amikacin	-235 (-4924 - 4325)	-301 (-2237 - 1553)	66 (-2688 - 2772)	91-100(neg) 81-90(neg)	61-70(pos) 51-60(pos)
Acinetobacter species	Aminoglycosides	-158 (-2590 - 2238)	-258 (-1304 - 778)	100 (-1286 - 1459)	91-100(neg) 81-90(neg)	51-60(pos) 61-70(pos)
Acinetobacter species	Carbapenems	-7 (-2302 - 2292)	-141 (-1144 - 852)	134 (-1158 - 1440)	91-100(neg) 81-90(neg)	61-70(pos) 51-60(pos)
Acinetobacter species	Fluroquinolones	-80 (-2291 - 2124)	-178 (-1144 - 769)	99 (-1147 - 1354)	91-100(neg) 81-90(neg)	51-60(pos) 61-70(pos)
Acinetobacter species	multi resistance (pseaer)	-86 (-2411 - 2238)	-197 (-1216 - 815)	111 (-1195 - 1422)	91-100(neg) 81-90(neg)	51-60(pos) 61-70(pos)
Enterococcus faecalis	Aminopenicillins	4 (-711 - 787)	3 (-325 - 375)	1 (-386 - 412)	91-100NA 61-70(pos)	91-100NA 51-60(pos)
Enterococcus faecalis	High-level aminoglycoside	30 (-4337 - 4515)	-144 (-2027 - 1749)	175 (-2310 - 2766)	91-100(neg) 81-90(neg)	71-80(pos) 61-70(pos)
Enterococcus faecalis	Vancomycin	4 (-2444 - 2445)	-20 (-1093 - 1054)	24 (-1350 - 1390)	71-80(neg) 81-90(neg)	51-60(pos) 61-70(pos)
Enterococcus faecium	Aminopenicillins	-153 (-3938 - 3665)	-70 (-1495 - 1371)	-84 (-2443 - 2293)	91-100(neg) 81-90(neg)	61-70(pos) 81-90(neg)

Enterococcus faecium	High-level aminoglycoside	-306 (-9737 - 9384)	-116 (-3217 - 3041)	-190 (-6519 - 6343)	91-100(neg) 81-90(neg)	91-100(neg) 81-90(neg)
Enterococcus faecium	Vancomycin	-358 (-5715 - 4990)	199 (-1731 - 2127)	-557 (-3984 - 2863)	91-100(neg) 61-70(pos)	91-100(neg) 81-90(neg)
Escherichia coli	Amikacin	41 (-10784 - 10907)	-218 (-5756 - 5502)	258 (-5028 - 5405)	81-90(neg) 71-80(neg)	81-90(pos) 71-80(pos)
Escherichia coli	Aminoglycosides	-582 (-10057 - 9017)	-4242 (-8968 - 514)	3660 (-1089 - 8502)	81-90(neg) 71-80(neg)	81-90(pos) 71-80(pos)
Escherichia coli	Aminopenicillins	778 (-14301 - 15767)	-4044 (-11824 - 3709)	4822 (-2477 - 12058)	71-80(neg) 81-90(neg)	61-70(pos) 71-80(pos)
Escherichia coli	Carbapenems	-328 (-18381 - 17944)	-1970 (-10839 - 7211)	1643 (-7541 - 10733)	81-90(neg) 71-80(neg)	81-90(pos) 71-80(pos)
Escherichia coli	Third-generation cephalosporins	-1506 (-13631 - 10474)	-7870 (-13976 - -1858)	6364 (346 - 12331)	81-90(neg) 71-80(neg)	81-90(pos) 71-80(pos)
Escherichia coli	Ertapenem	-1648 (-54064 - 49132)	-2860 (-27963 - 20698)	1212 (-26101 - 28434)	71-80(neg) 81-90(neg)	71-80(pos) 51-60(pos)
Escherichia coli	multi resistance (esccol)	-1104 (-9768 - 7507)	-3569 (-7916 - 739)	2465 (-1852 - 6768)	61-70(neg) 71-80(neg)	71-80(pos) 81-90(pos)
Escherichia coli	Fluoroquinolones	-1726 (-13947 - 10579)	-11332 (-17502 - -5194)	9606 (3554 - 15773)	81-90(neg) 71-80(neg)	81-90(pos) 71-80(pos)
Escherichia coli	multi resistance (pseaer)	-794 (-35434 - 25180)	-732 (-19024 - 9911)	-61 (-16410 - 15269)	71-80(neg) 81-90(neg)	71-80(neg) 81-90(neg)
Escherichia coli	piperacillin-tazobactam	-2 (-32920 - 32692)	-1996 (-19007 - 13799)	1994 (-13912 - 18893)	81-90(neg) 71-80(neg)	81-90(pos) 71-80(pos)
Klebsiella pneumoniae	Amikacin	-235 (-17721 - 17371)	-633 (-6698 - 5515)	398 (-11023 - 11856)	71-80(neg) 81-90(neg)	51-60(pos) 61-70(pos)

Klebsiella pneumoniae	Aminoglycosides	532 (-6768 - 7848)	-1939 (-4884 - 996)	2471 (-1885 - 6852)	81-90(neg) 71-80(neg)	51-60(pos) 61-70(pos)
Klebsiella pneumoniae	Carbapenems	754 (-9666 - 11278)	-824 (-4600 - 2982)	1578 (-5067 - 8296)	71-80(neg) 81-90(neg)	61-70(pos) 51-60(pos)
Klebsiella pneumoniae	Third-generation cephalosporins	688 (-7227 - 8580)	-2202 (-5439 - 990)	2890 (-1789 - 7590)	81-90(neg) 71-80(neg)	51-60(pos) 61-70(pos)
Klebsiella pneumoniae	Ertapenem	1336 (-26001 - 28560)	-538 (-10268 - 9323)	1875 (-15734 - 19238)	91-100(neg) 81-90(neg)	61-70(pos) 51-60(pos)
Klebsiella pneumoniae	Fluoroquinolones	314 (-7543 - 8220)	-2568 (-5775 - 652)	2882 (-1768 - 7568)	81-90(neg) 71-80(neg)	51-60(pos) 61-70(pos)
Klebsiella pneumoniae	multi resistance (klepne)	384 (-6850 - 7635)	-1918 (-4820 - 982)	2302 (-2030 - 6653)	71-80(neg) 81-90(neg)	51-60(pos) 61-70(pos)
Klebsiella pneumoniae	multi resistance (pseaer)	-401 (-28240 - 27377)	-1271 (-11337 - 8611)	870 (-16904 - 18766)	71-80(neg) 81-90(neg)	81-90(neg) 51-60(pos)
Klebsiella pneumoniae	piperacillin-tazobactam	622 (-19480 - 20977)	-1896 (-9245 - 5646)	2518 (-10236 - 15331)	71-80(neg) 81-90(neg)	51-60(pos) 61-70(pos)
Pseudomonas aeruginosa	Amikacin	13 (-2213 - 2255)	-88 (-1014 - 848)	101 (-1198 - 1407)	71-80(neg) 81-90(neg)	81-90(neg) 51-60(pos)
Pseudomonas aeruginosa	Aminoglycoside	50 (-2733 - 2836)	-143 (-1328 - 1026)	192 (-1404 - 1809)	71-80(neg) 81-90(neg)	61-70(pos) 51-60(pos)
Pseudomonas aeruginosa	Carbapenem	528 (-3294 - 4354)	-34 (-1646 - 1577)	562 (-1648 - 2777)	71-80(neg) 81-90(neg)	81-90(neg) 51-60(pos)
Pseudomonas aeruginosa	Ceftazidime	322 (-3613 - 4302)	-94 (-1759 - 1588)	416 (-1853 - 2714)	71-80(neg) 81-90(neg)	81-90(neg) 51-60(pos)
Pseudomonas aeruginosa	Fluoroquinolone	206 (-3810 - 4231)	-328 (-1998 - 1358)	534 (-1811 - 2873)	71-80(neg) 81-90(neg)	61-70(pos) 51-60(pos)

Pseudomonas aeruginosa	multi resistance (pseaer)	191 (-3063 - 3487)	-169 (-1527 - 1208)	360 (-1535 - 2278)	71-80(neg) 81-90(neg)	81-90(neg) 51-60(pos)
Pseudomonas aeruginosa	piperacillin-tazobactam	334 (-4094 - 4789)	-152 (-2004 - 1719)	486 (-2090 - 3071)	71-80(neg) 81-90(neg)	81-90(neg) 51-60(pos)
Staphylococcus aureus	Fluoroquinolone	-1919 (-8160 - 4386)	-834 (-3401 - 1750)	-1085 (-4760 - 2637)	51-60(neg) 81-90(pos)	51-60(neg) 81-90(pos)
Staphylococcus aureus	MRSA (oxacillin or cefoxitin)	-910 (-6973 - 5103)	-396 (-2861 - 2033)	-514 (-4113 - 3070)	51-60(neg) 81-90(pos)	51-60(neg) 81-90(pos)
Staphylococcus aureus	Rifampicin	-2 (-2144 - 2137)	-95 (-975 - 769)	94 (-1168 - 1368)	91-100(neg) 81-90(neg)	51-60(pos) 61-70(pos)
Streptococcus pneumoniae	cefIII (strepne)	3 (-1690 - 1736)	14 (-706 - 780)	-11 (-983 - 955)	81-90(pos) 1-10(pos)	1-10(pos) 61-70(neg)
Streptococcus pneumoniae	efq strepne	-161 (-3752 - 3437)	-74 (-1579 - 1418)	-87 (-2173 - 2018)	11-20(neg) 1-10(neg)	41-50(neg) 61-70(neg)
Streptococcus pneumoniae	Macrolide	26 (-2993 - 3078)	136 (-1241 - 1526)	-110 (-1751 - 1551)	91-100(pos) 81-90(pos)	51-60(neg) 61-70(neg)
Streptococcus pneumoniae	Penicillins	248 (-3702 - 4237)	142 (-1647 - 1927)	106 (-2056 - 2309)	91-100(pos) 1-10(pos)	51-60(neg) 1-10(pos)
Streptococcus pneumoniae	multi resistance (strpne)	139 (-2960 - 3280)	138 (-1253 - 1544)	0 (-1706 - 1735)	81-90(pos) 1-10(pos)	61-70(neg) 1-10(pos)
