# Supplementary material for: Combining demographic shifts with age-based resistance prevalence to estimate future antimicrobial resistance burden in Europe and implications for targets: A modelling study
Source: PLoS Med. 2025 Nov 4;22(11):e1004579. doi: 10.1371/journal.pmed.1004579 (PMC12585039; doi:10.1371/journal.pmed.1004579)
Supplement: S2 Appendix — Colour indicates country. Dashed lines indicate −0.7 and 0.7, equating to half and double the actual value. Fig A2: Predicted BSIs in 2023 under fixed or linear trends (type, colour) compared to ECDC-reported data (black point). A) Acinetobacter spp.|Aminoglycosides B) Acinetobacter spp.|Carbapenems. Fig A3: Predicted BSIs in 2023 under fixed or linear trends (type, colour) compared to ECDC-reported data (black point). A) Acinetobacter spp.|Fluoroquinolones B) Enterococcus faecalis|Aminopenicillins. Fig A4: Predicted BSIs in 2023 under fixed or linear trends (type, colour) compared to ECDC-reported data (black point). A) E. faecalis|High-level gentamicin B) E. faecalis|Vancomycin. Fig A5: Predicted BSIs in 2023 under fixed or linear trends (type, colour) compared to ECDC-reported data (black point). A) E. faecium|Aminopenicillins B) E. faecium|High-level gentamicin. Fig A6: Predicted BSIs in 2023 under fixed or linear trends (type, colour) compared to ECDC reported data (black point). A) E. faecium|Vancomycin B) Escherichia coli|Aminoglycosides. Fig A7: Predicted BSIs in 2023 under fixed or linear trends (type, colour) compared to ECDC-reported data (black point). A) E. coli|Aminopenicillins B) E. coli|Carbapenems. Fig A8: Predicted BSIs in 2023 under fixed or linear trends (type, colour) compared to ECDC reported data (black point). A) E. coli|Fluoroquinolones B) Klebsiella pneumoniae|Aminoglycosides. Fig A9: Predicted BSIs in 2023 under fixed or linear trends (type, colour) compared to ECDC-reported data (black point). A) K. pneumoniae|Carbapenems B) Pseudomonas aeruginosa|Aminoglycosides. Fig A10: Predicted BSIs in 2023 under fixed or linear trends (type, colour) compared to ECDC-reported data (black point). A) P. aeruginosa|Carbapenems B) P. aeruginosa|Ceftazidime. Fig A11: Predicted BSIs in 2023 under fixed or linear trends (type, colour) compared to ECDC-reported data (black point). A) P. aeruginosa|Fluoroquinolones B) Staphylococcus aureus|Meticillin (MRSA). [file pmed.1004579.s002.pdf]

S2 Appendix

Contents

1 - Country names 1

2 - Validation of incidence 1

3 - Including age/sex resistance rates 1

1 - Country names

AT is Austria, BE is Belgium, BG is Bulgaria, CY is Cyprus, CZ is Czechia, DE is Germany, DK is Denmark, EE is Estonia, EL is Greece, ES is Spain, FI is Finland, FR is France, HR is Croatia, HU is Hungary, IE is Ireland, IS is Iceland, IT is Italy, LI is Liechtenstein, LT is Lithuania, LU is Luxembourg, LV is Latvia, MT is Malta, NL is Netherlands, NO is Norway, PL is Poland, PT is Portugal, RO is Romania, SE is Sweden, SI is Slovenia and SK is Slovakia.

2 - Validation of incidence

Total BSI incidence in 2023 was compared to model predicted 2023 BSI incidence. Antibiotic groupings in the 2023 report were slightly different than in the more disaggregated 2015-2020 data used for predictions, so small differences in classification may exist, and they 2023 data is not age or sex stratified.

Figure A1 shows the log error comparing estimated vs actual data. The subsequent figures (A2-A12) show the predictions against the data.

3 - Including age/sex resistance rates

Total resistant BSIs per age and sex in 2030 were compared between the "base" model that assumed a constant proportion resistant across age and sexes, and the "agesex" model that allowed for variation in resistance by age and sex (see methods, Figure 1). The following plots (A13-A50) show a breakdown of the comparison by age and sex, and also by country.

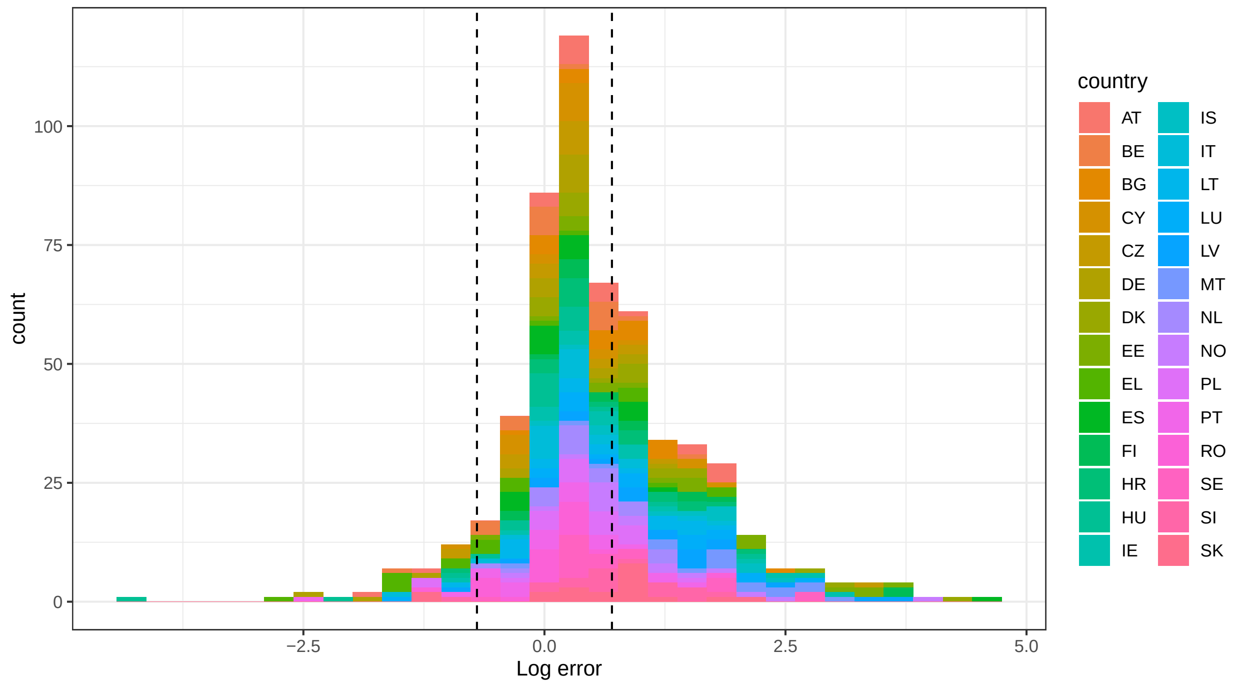

Figure A1: Log error of predicted BSIs in 2023 compared to ECDC reported data. Colour indicates country. Dashed lines indicate -0.7 and 0.7, equating to half and double the actual value.

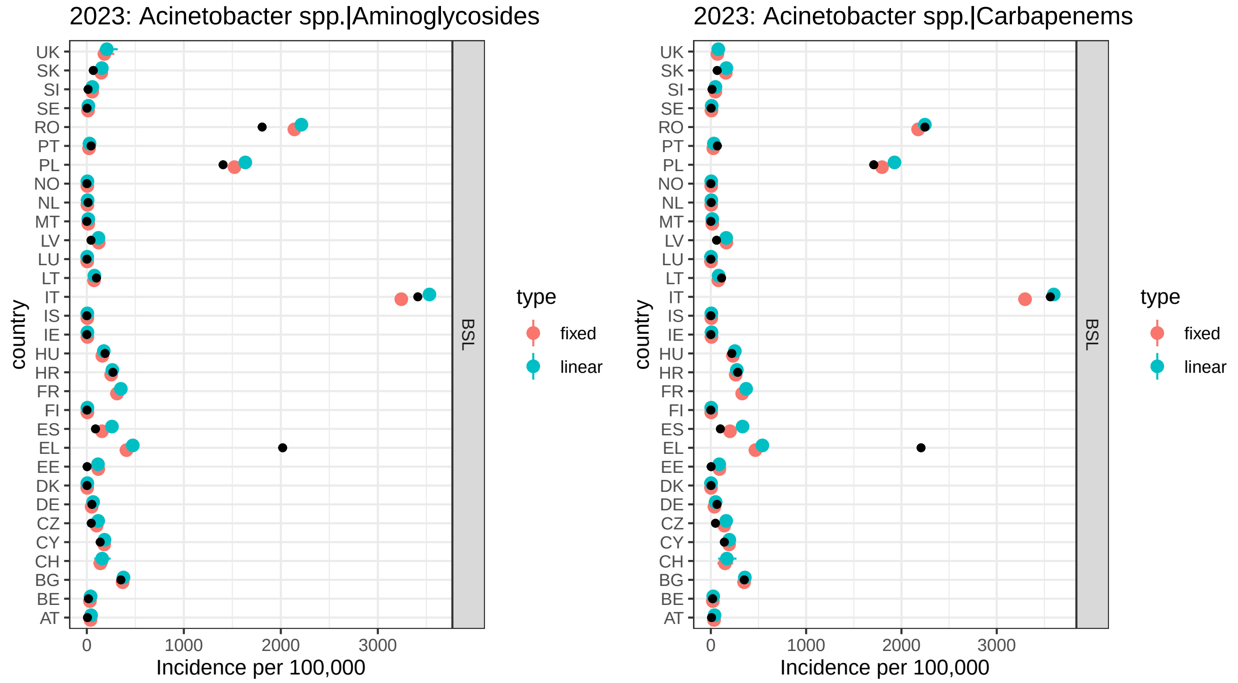

Figure A2: Predicted BSIs in 2023 under fixed or linear trends (type, colour) compared to ECDC reported data (black point). A) Acinetobacter spp.|Aminoglycosides B) Acinetobacter spp.|Carbapenems.

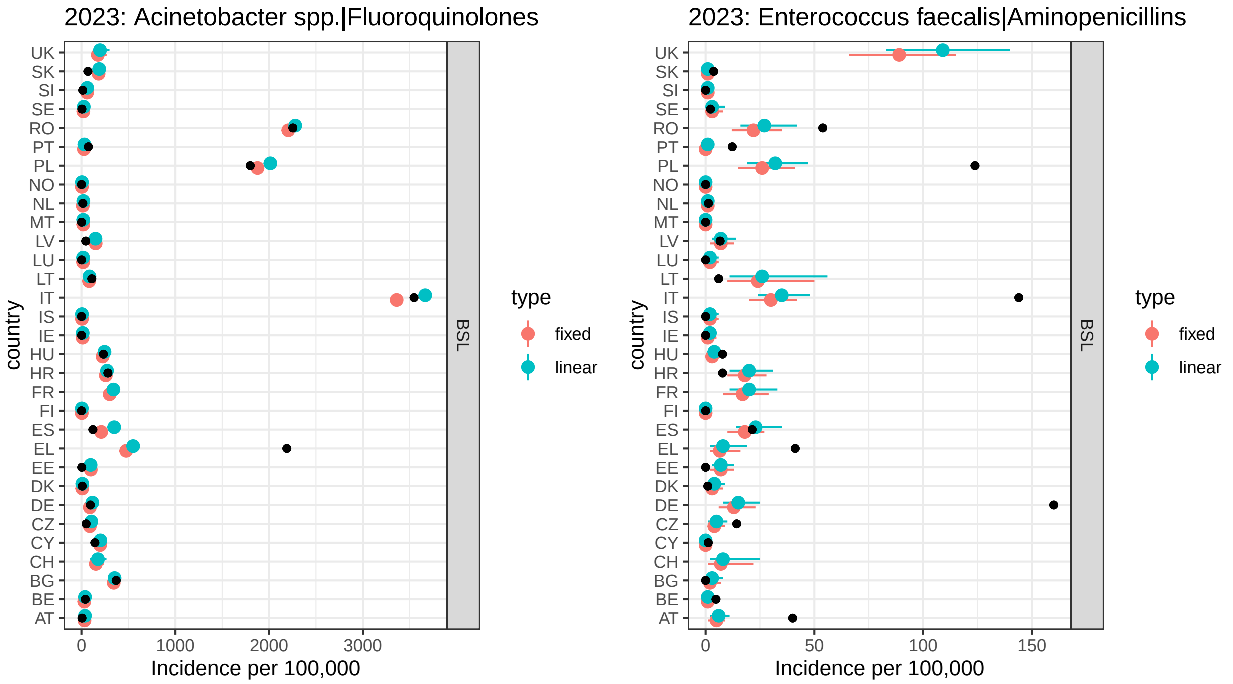

Figure A3: Predicted BSIs in 2023 under fixed or linear trends (type, colour) compared to ECDC reported data (black point).A) Acinetobacter spp.|Fluoroquinolones B) Enterococcus faecalis|Aminopenicillins.

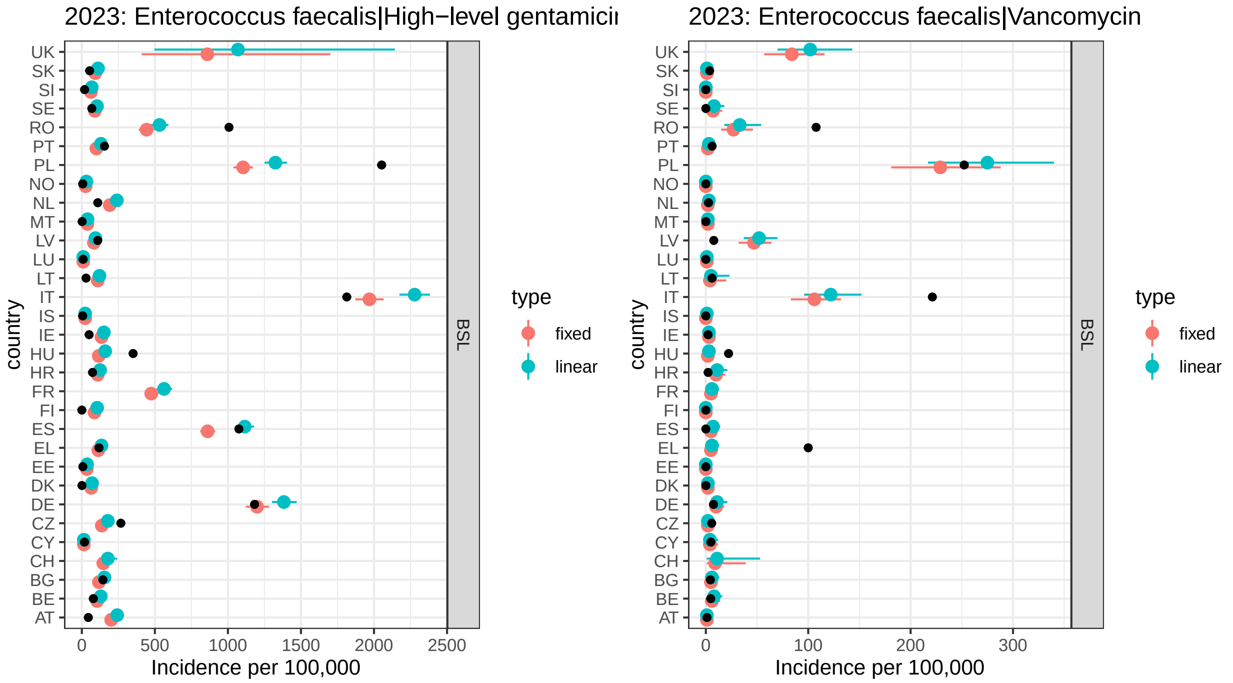

Figure A4: Predicted BSIs in 2023 under fixed or linear trends (type, colour) compared to ECDC reported data (black point).A) Enterococcus faecalis|High-level gentamicin B) Enterococcus faecalis|Vancomycin.

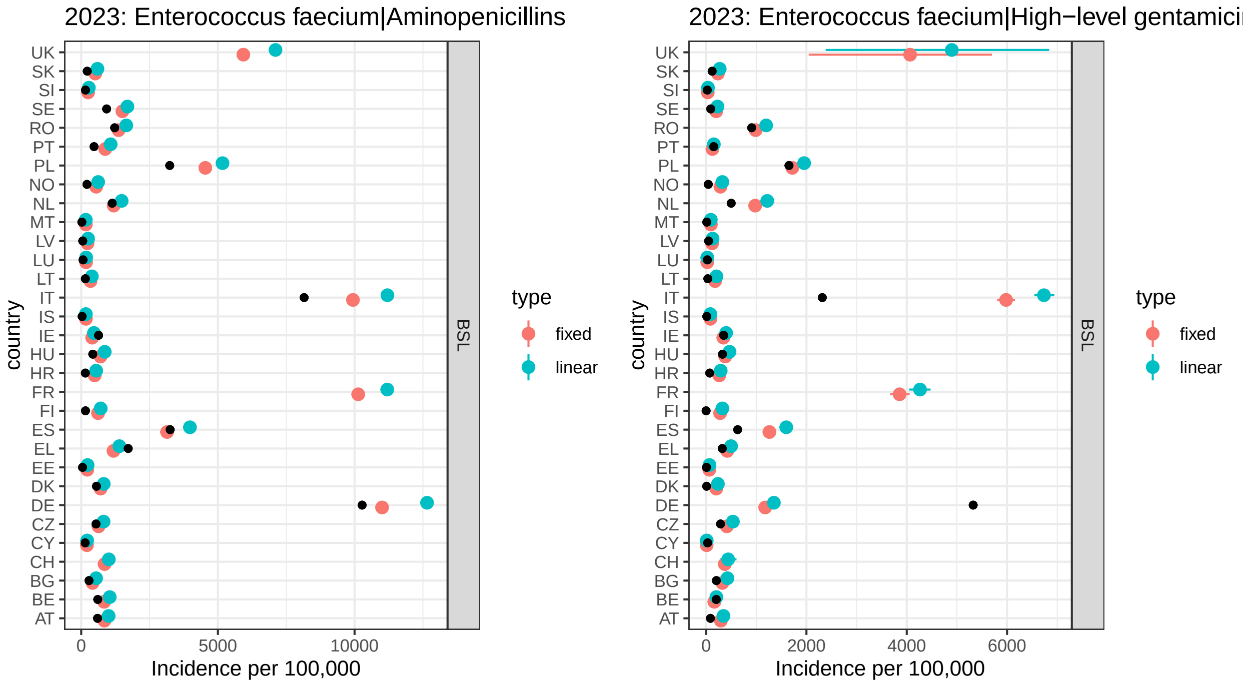

Figure A5: Predicted BSIs in 2023 under fixed or linear trends (type, colour) compared to ECDC reported data (black point).A) Enterococcus faecium|Aminopenicillins B) Enterococcus faecium|High-level gentamicin.

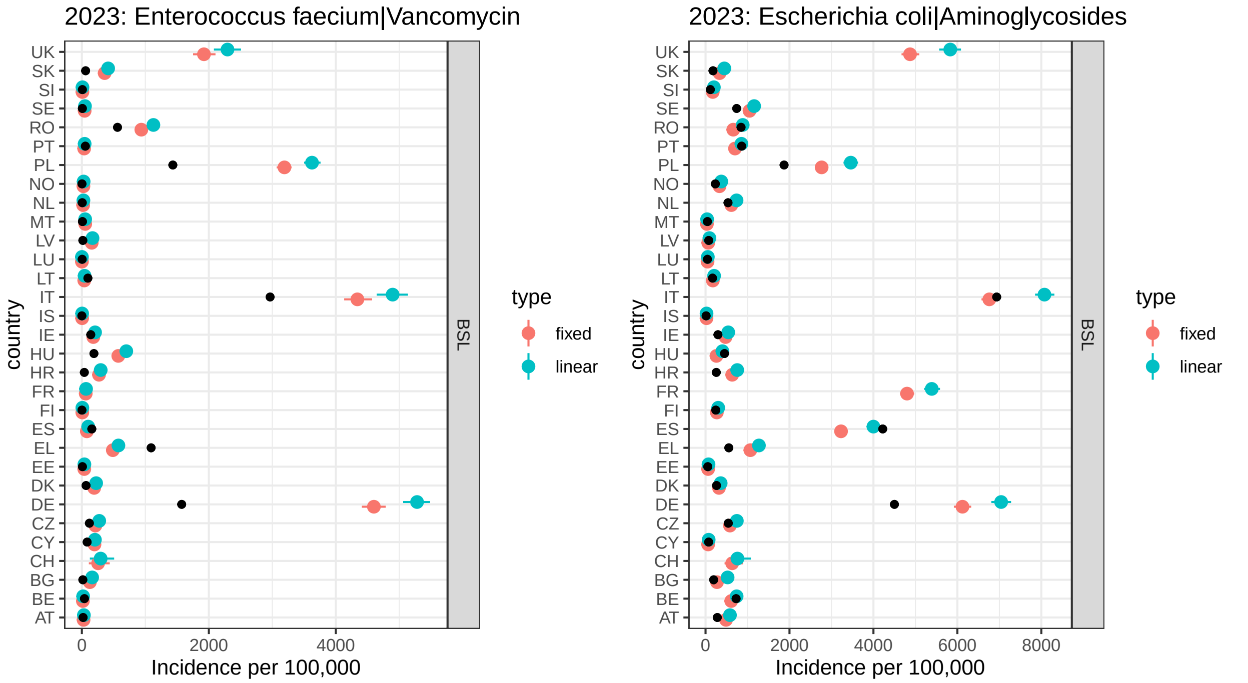

Figure A6: Predicted BSIs in 2023 under fixed or linear trends (type, colour) compared to ECDC reported data (black point).A) Enterococcus faecium|Vancomycin B) Escherichia coli|Aminoglycosides.

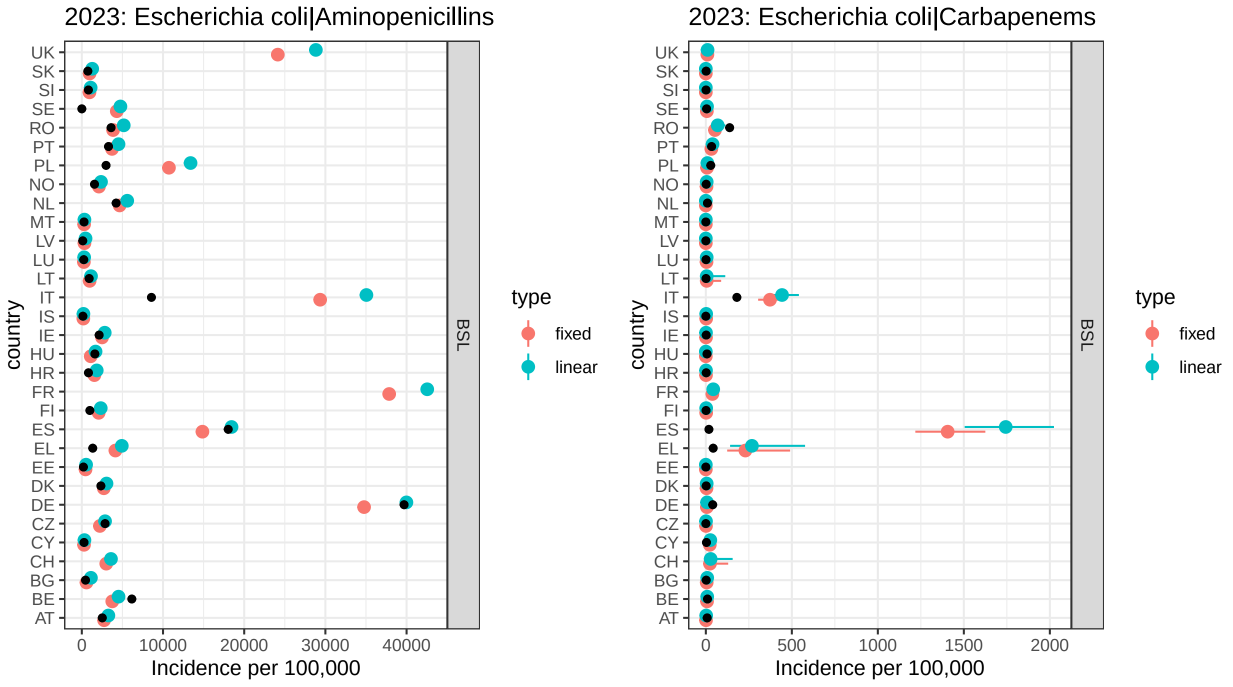

Figure A7: Predicted BSIs in 2023 under fixed or linear trends (type, colour) compared to ECDC reported data (black point).A) Escherichia coli|Aminopenicillins B) Escherichia coli|Carbapenems.

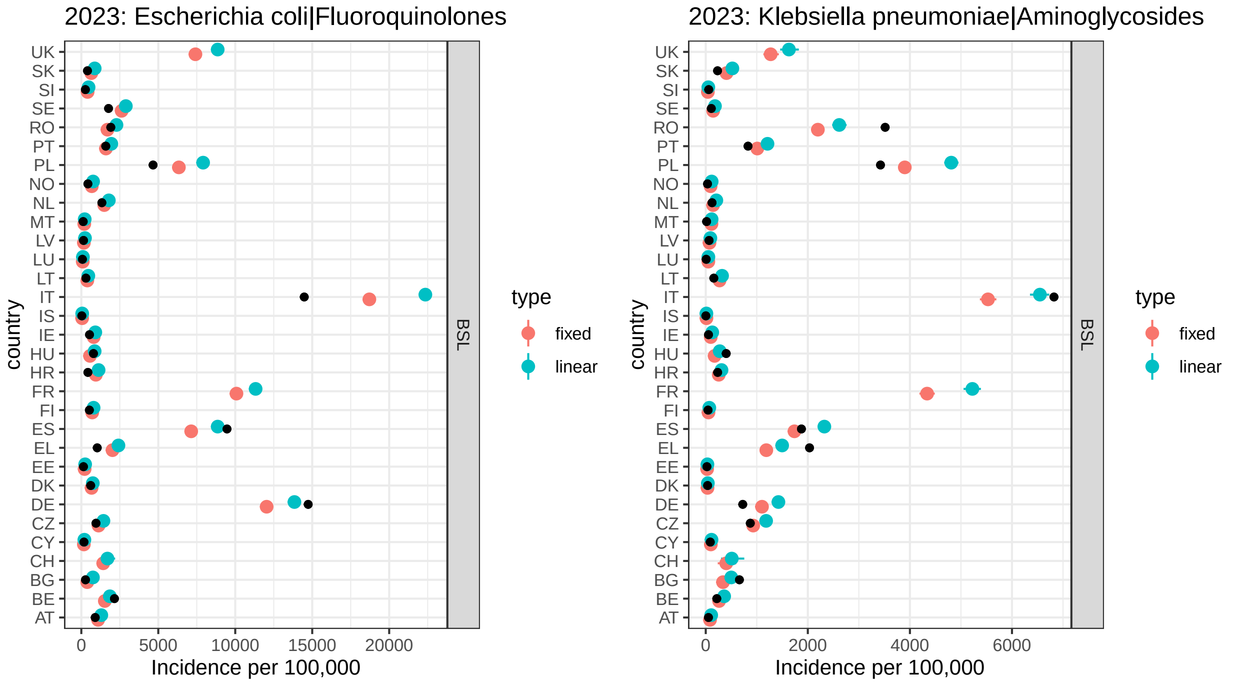

Figure A8: Predicted BSIs in 2023 under fixed or linear trends (type, colour) compared to ECDC reported data (black point).A) Escherichia coli|Fluoroquinolones B) Klebsiella pneumoniae|Aminoglycosides.

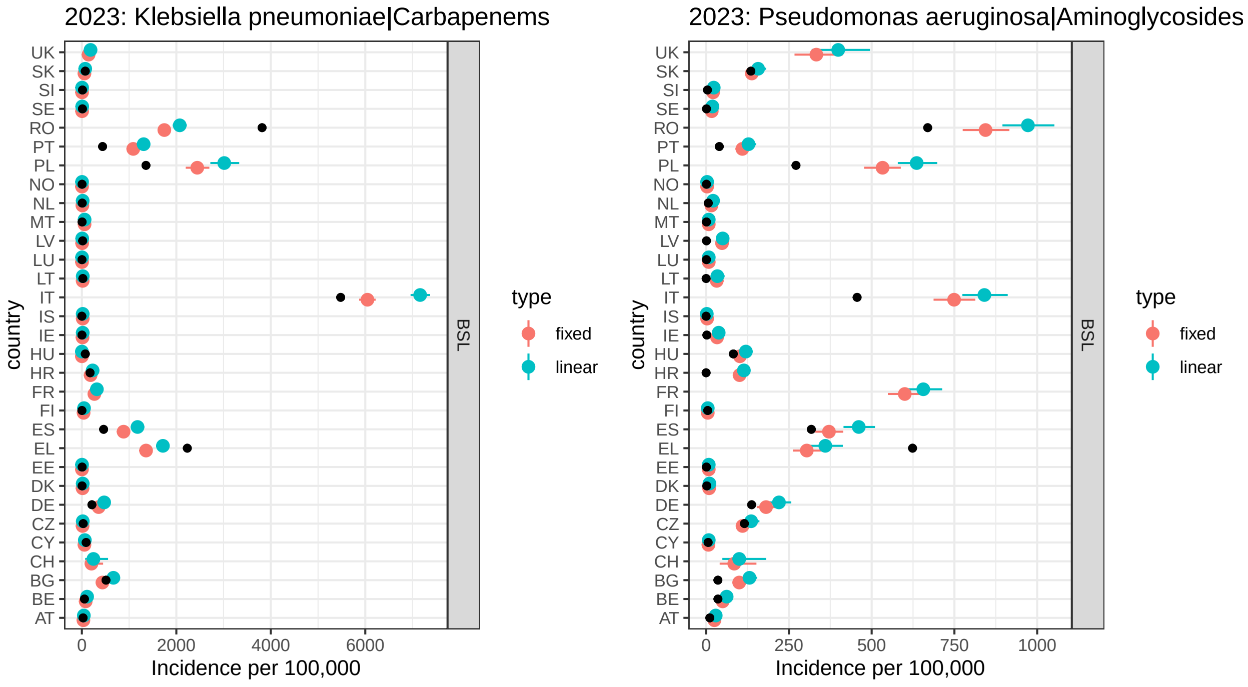

Figure A9: Predicted BSIs in 2023 under fixed or linear trends (type, colour) compared to ECDC reported data (black point).A) Klebsiella pneumoniae|Carbapenems B) Pseudomonas aeruginosa|Aminoglycosides.

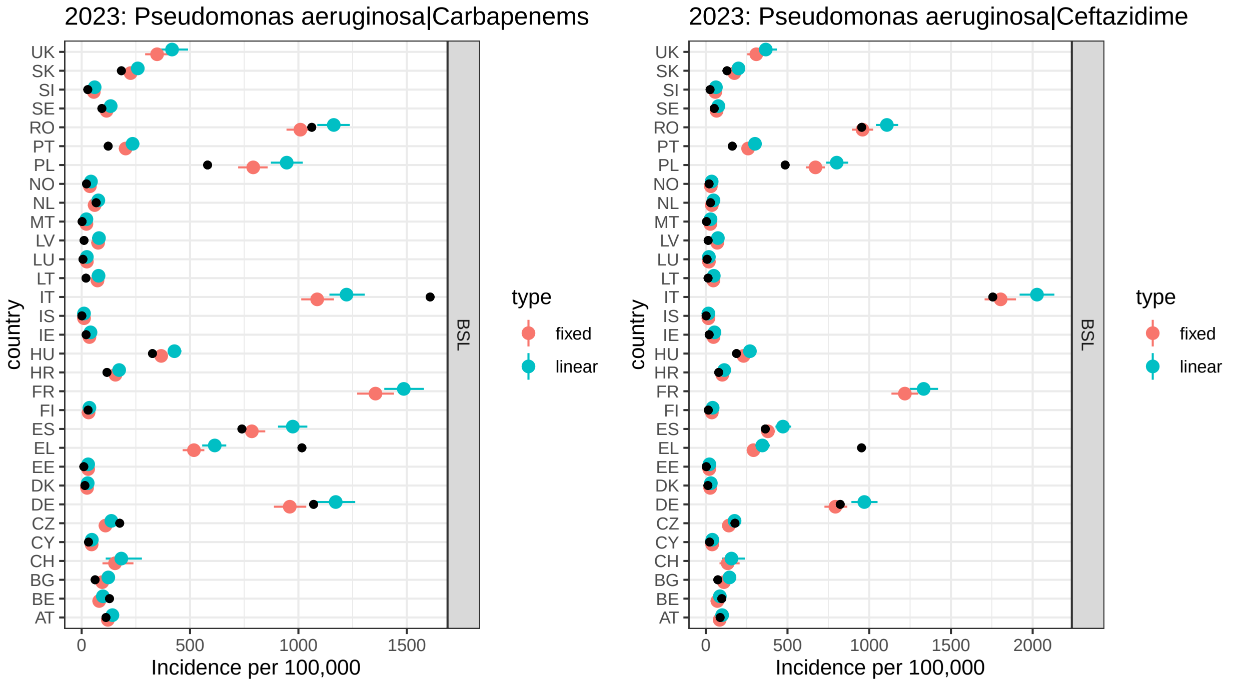

Figure A10: Predicted BSIs in 2023 under fixed or linear trends (type, colour) compared to ECDC reported data (black point).A) Pseudomonas aeruginosa|Carbapenems B) Pseudomonas aeruginosa|Ceftazidime.

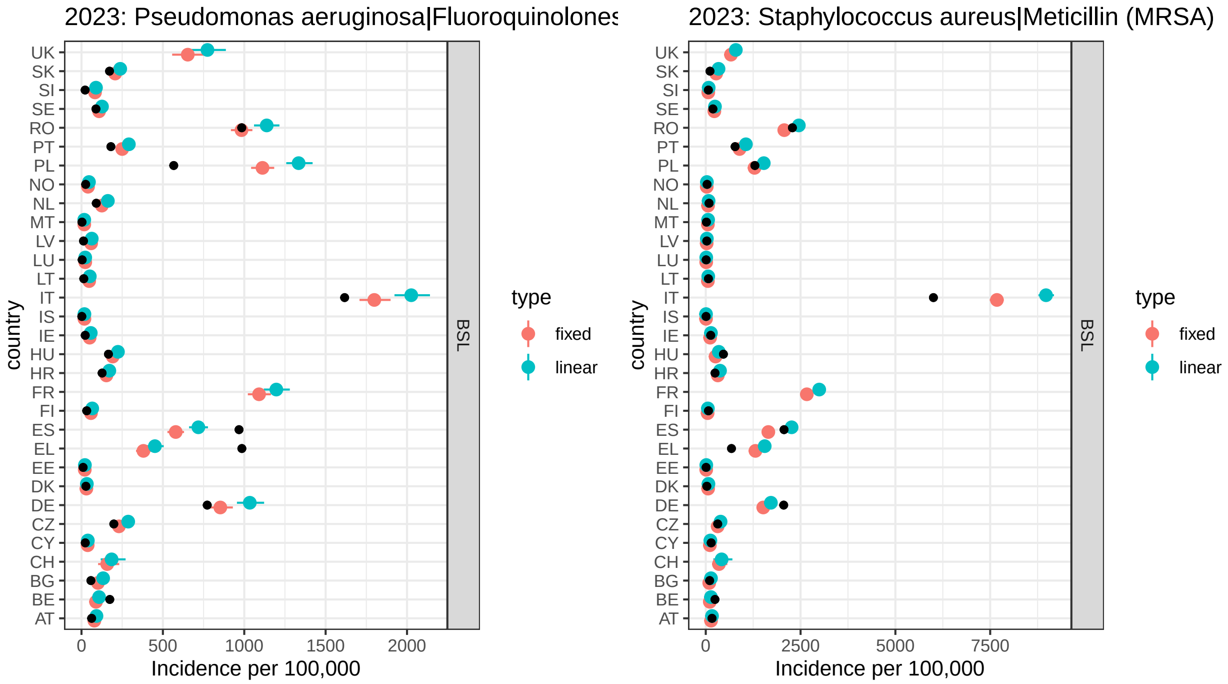

Figure A11: Predicted BSIs in 2023 under fixed or linear trends (type, colour) compared to ECDC reported data (black point).A) Pseudomonas aeruginosa|Fluoroquinolones B) Staphylococcus aureus|Meticillin (MRSA).

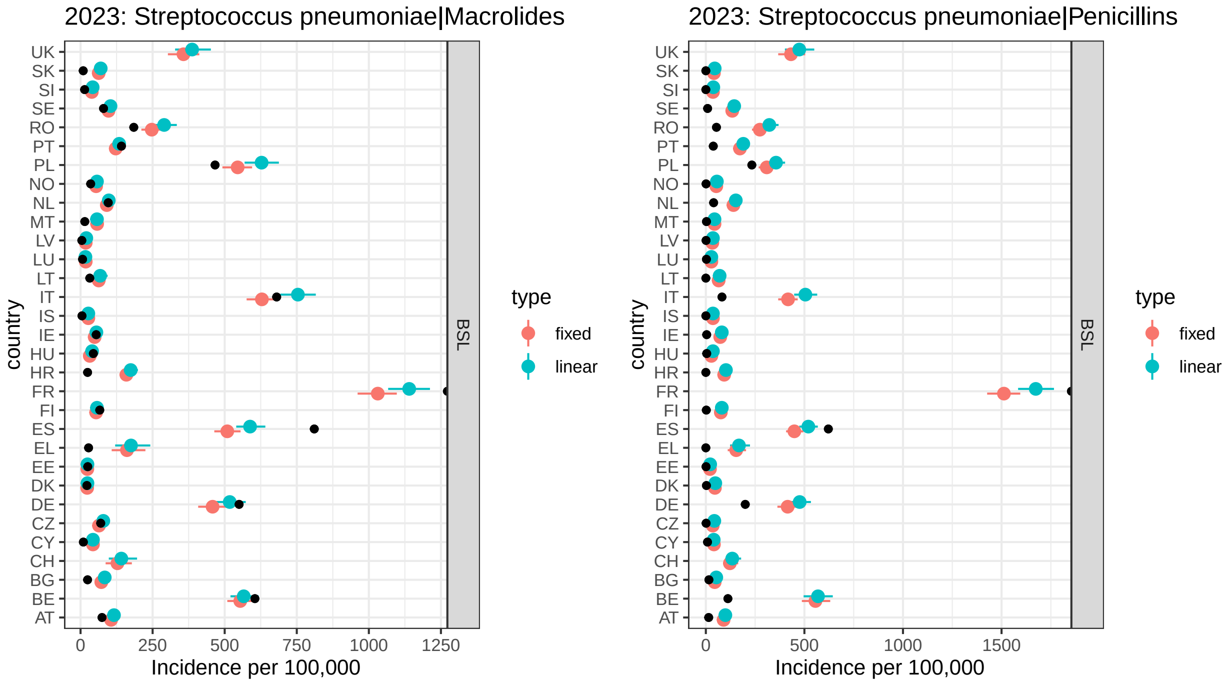

Figure A12: Predicted BSIs in 2023 under fixed or linear trends (type, colour) compared to ECDC reported data (black point).A) Streptococcus pneumoniae|Macrolides B) Streptococcus pneumoniae|Penicillins.

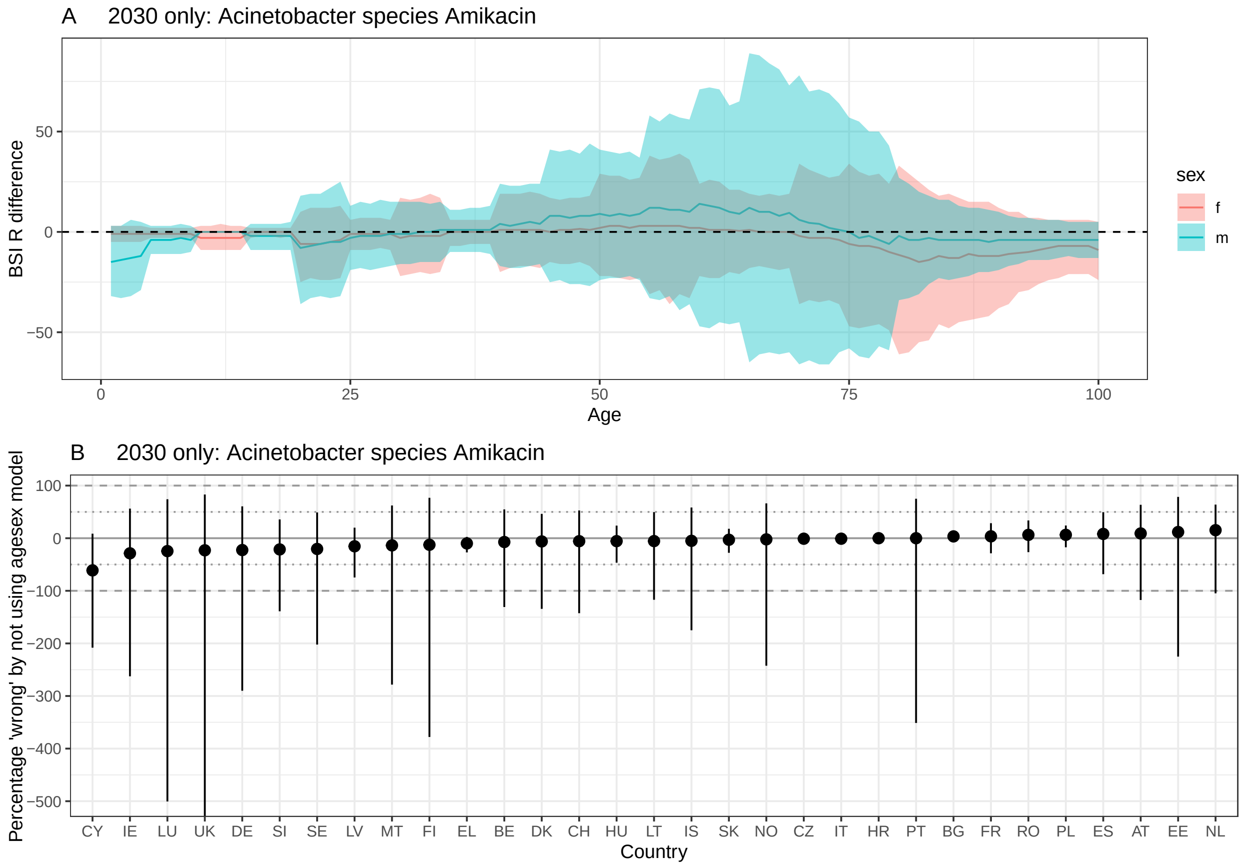

Figure A13: Difference between 'agesex' and 'base' model projections in 2030. A positive difference indicates that using the 'base' model underestimates resistant BSIs compared to the 'agesex' model. A) by age and sex. The line depicts the median and the ribbon the 95% quantiles. The dashed line at 0 indicates no difference. B) by country. The solid line indicates no difference, and the dotted / dashed lines represent a 50 and 100% difference respectively. The dot depicts the median and the ribbon the 95% quantiles. Acinetobacter species Amikacin .

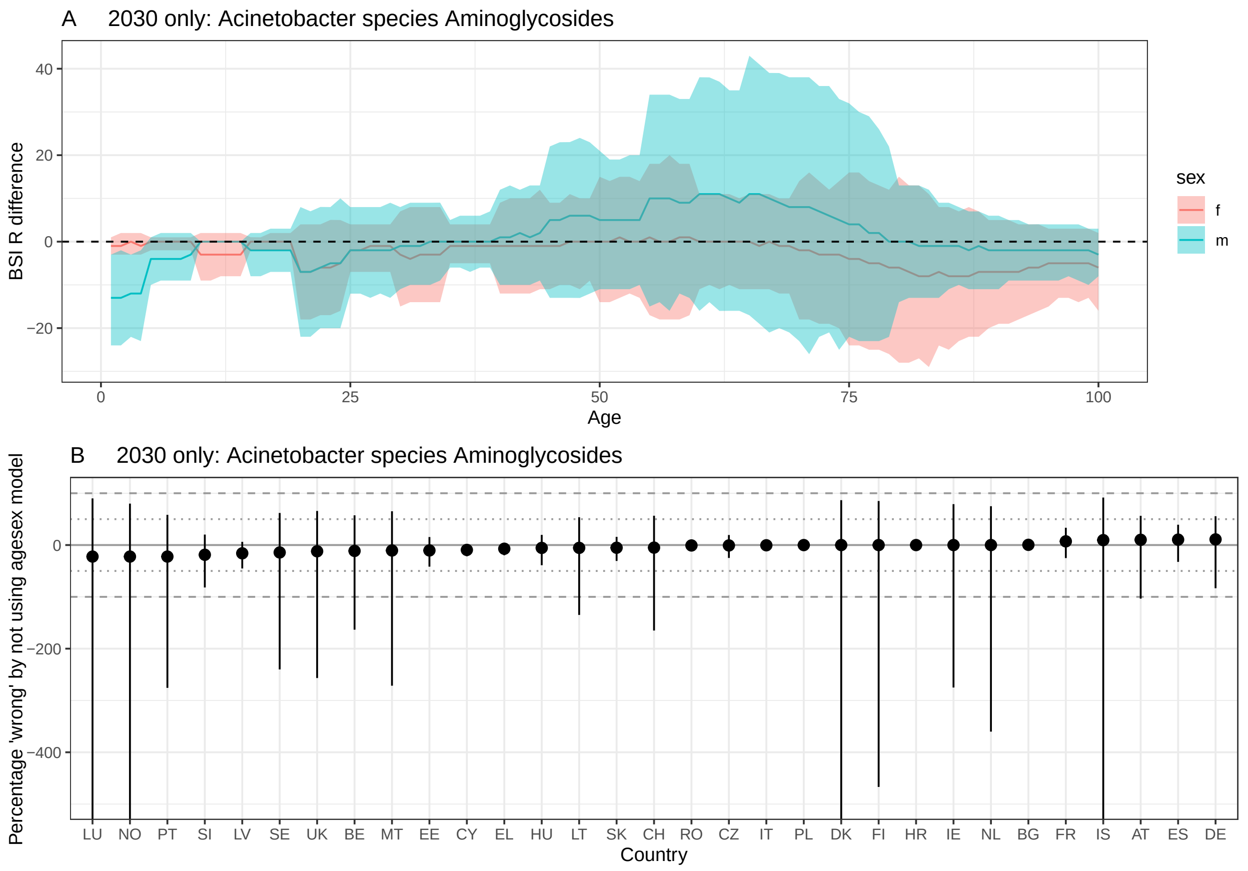

Figure A14: Difference between 'agesex' and 'base' model projections in 2030. A positive difference indicates that using the 'base' model underestimates resistant BSIs compared to the 'agesex' model. A) by age and sex. The line depicts the median and the ribbon the 95% quantiles. The dashed line at 0 indicates no difference. B) by country. The solid line indicates no difference, and the dotted / dashed lines represent a 50 and 100% difference respectively. The dot depicts the median and the ribbon the 95% quantiles. Acinetobacter species Aminoglycosides.

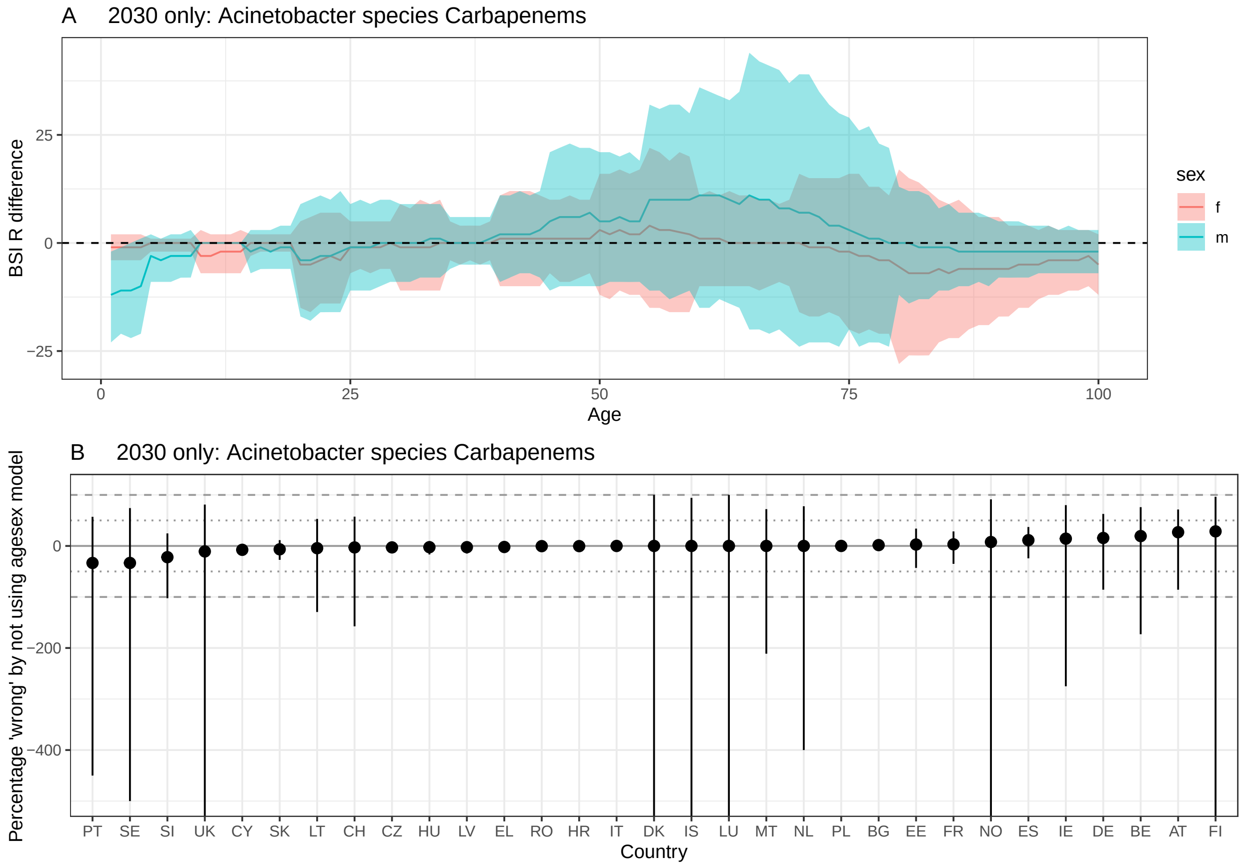

Figure A15: Difference between 'agesex' and 'base' model projections in 2030. A positive difference indicates that using the 'base' model underestimates resistant BSIs compared to the 'agesex' model. A) by age and sex. The line depicts the median and the ribbon the 95% quantiles. The dashed line at 0 indicates no difference. B) by country. The solid line indicates no difference, and the dotted / dashed lines represent a 50 and 100% difference respectively. The dot depicts the median and the ribbon the 95% quantiles. Acinetobacter species Carbapenems.

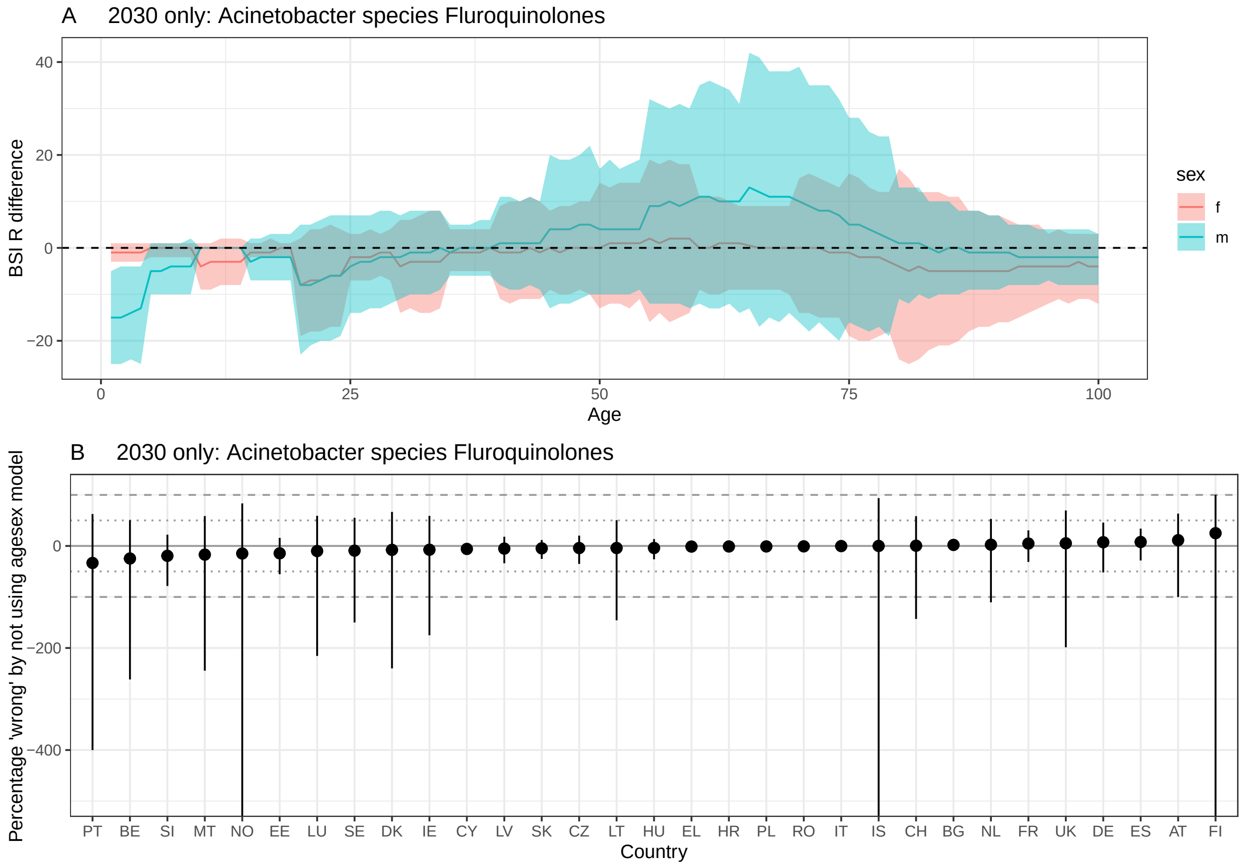

Figure A16: Difference between 'agesex' and 'base' model projections in 2030. A positive difference indicates that using the 'base' model underestimates resistant BSIs compared to the 'agesex' model. A) by age and sex. The line depicts the median and the ribbon the 95% quantiles. The dashed line at 0 indicates no difference. B) by country. The solid line indicates no difference, and the dotted / dashed lines represent a 50 and 100% difference respectively. The dot depicts the median and the ribbon the 95% quantiles. Acinetobacter species Fluroquinolones.

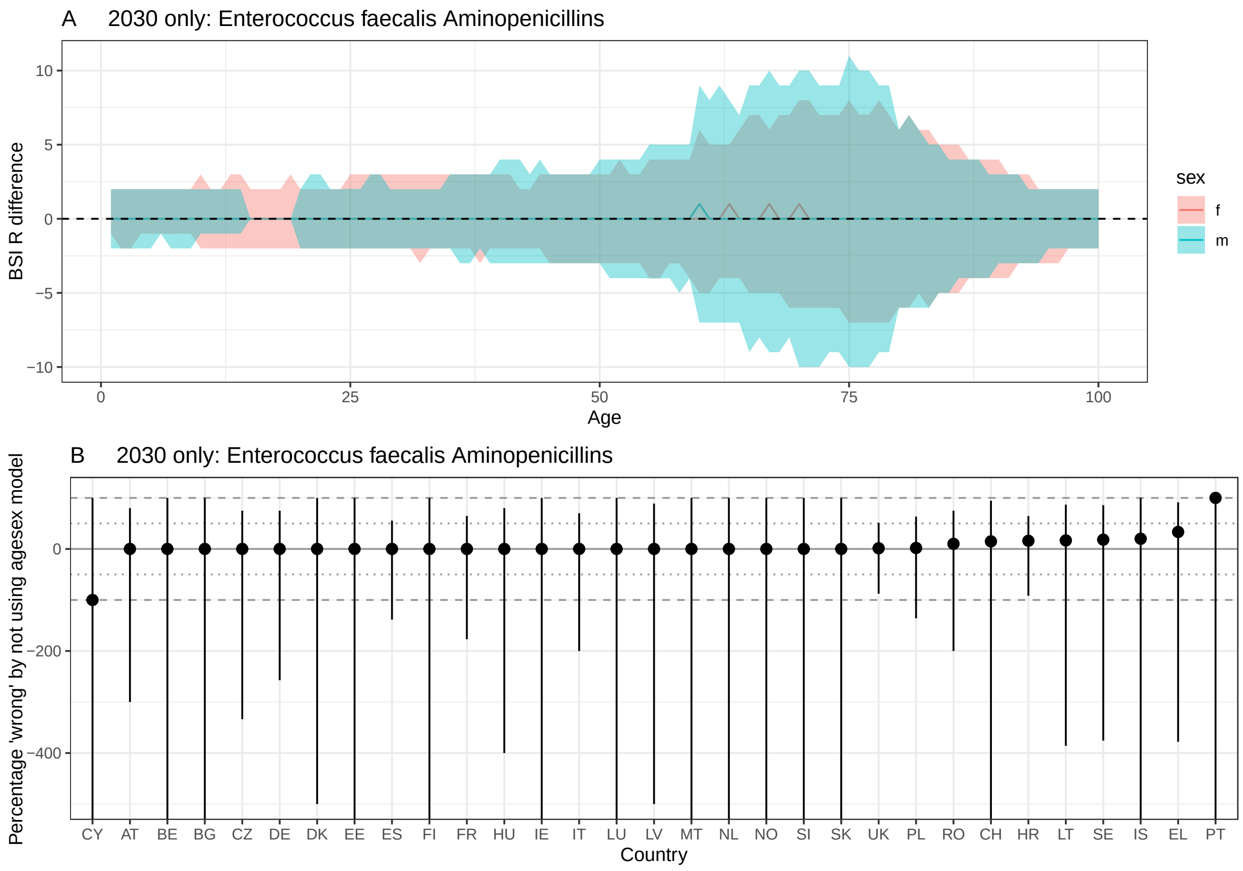

Figure A17: Difference between 'agesex' and 'base' model projections in 2030. A positive difference indicates that using the 'base' model underestimates resistant BSIs compared to the 'agesex' model. A) by age and sex. The line depicts the median and the ribbon the 95% quantiles. The dashed line at 0 indicates no difference. B) by country. The solid line indicates no difference, and the dotted / dashed lines represent a 50 and 100% difference respectively. The dot depicts the median and the ribbon the 95% quantiles. Enterococcus faecalis Aminopenicillins.

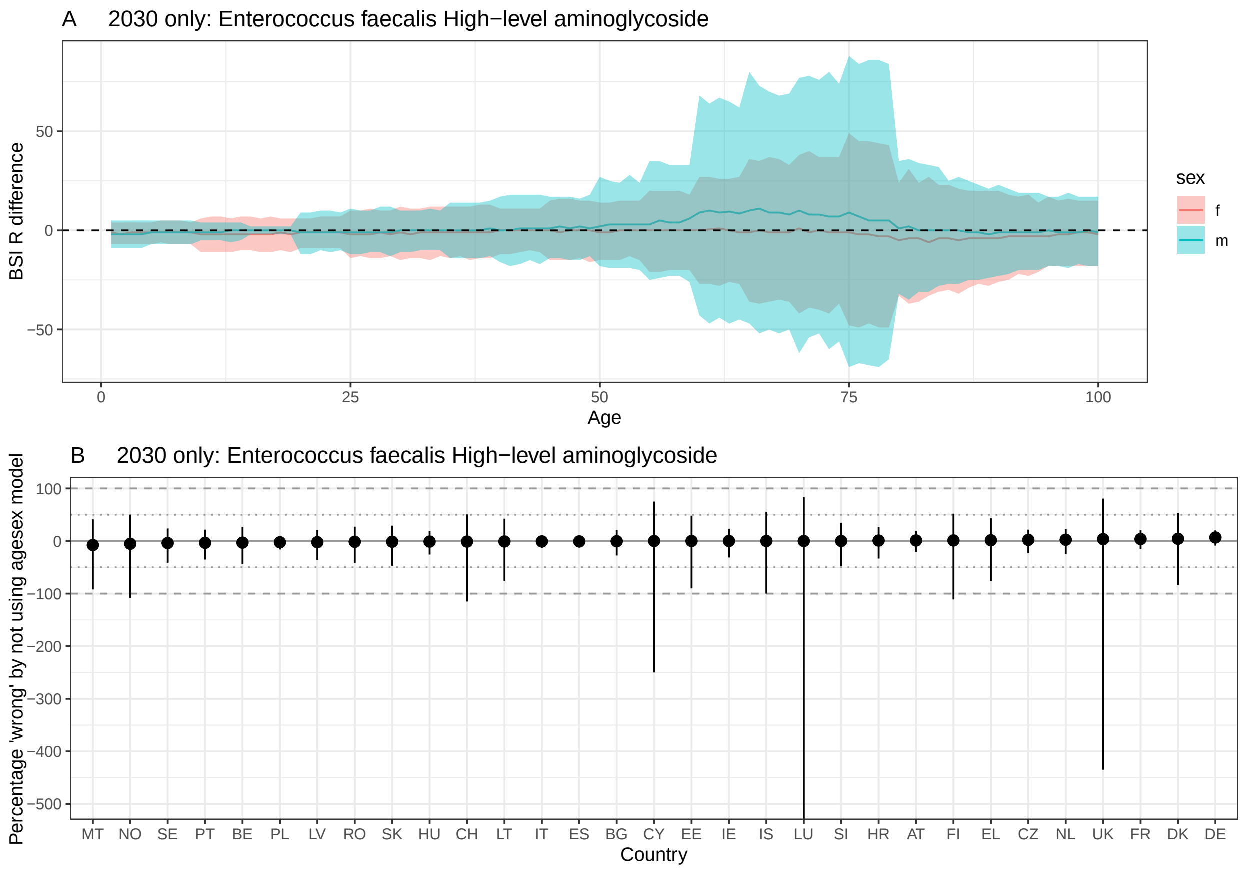

Figure A18: Difference between 'agesex' and 'base' model projections in 2030. A positive difference indicates that using the 'base' model underestimates resistant BSIs compared to the 'agesex' model. A) by age and sex. The line depicts the median and the ribbon the 95% quantiles. The dashed line at 0 indicates no difference. B) by country. The solid line indicates no difference, and the dotted / dashed lines represent a 50 and 100% difference respectively. The dot depicts the median and the ribbon the 95% quantiles. Enterococcus faecalis High-level aminoglycoside .

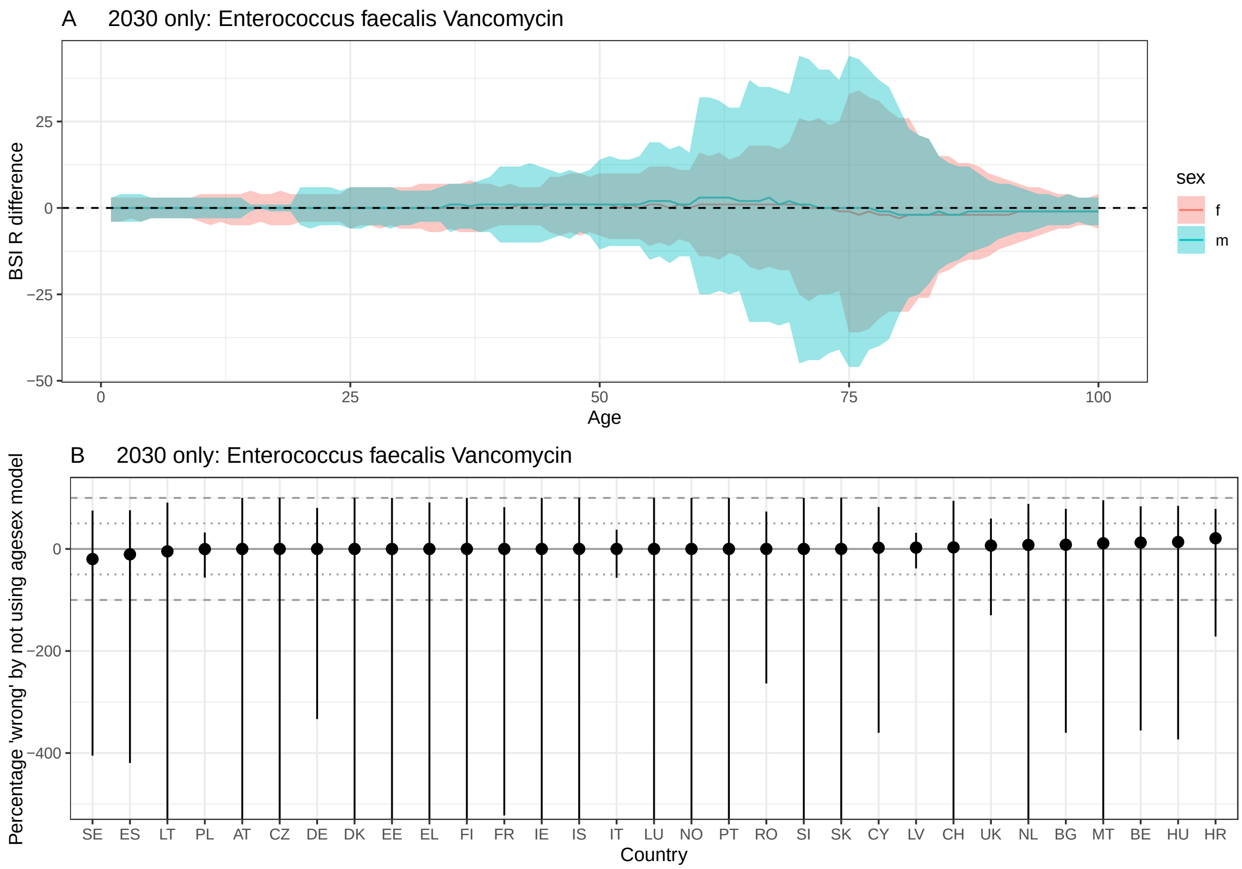

Figure A19: Difference between 'agesex' and 'base' model projections in 2030. A positive difference indicates that using the 'base' model underestimates resistant BSIs compared to the 'agesex' model. A) by age and sex. The line depicts the median and the ribbon the 95% quantiles. The dashed line at 0 indicates no difference. B) by country. The solid line indicates no difference, and the dotted / dashed lines represent a 50 and 100% difference respectively. The dot depicts the median and the ribbon the 95% quantiles. Enterococcus faecalis Vancomycin.

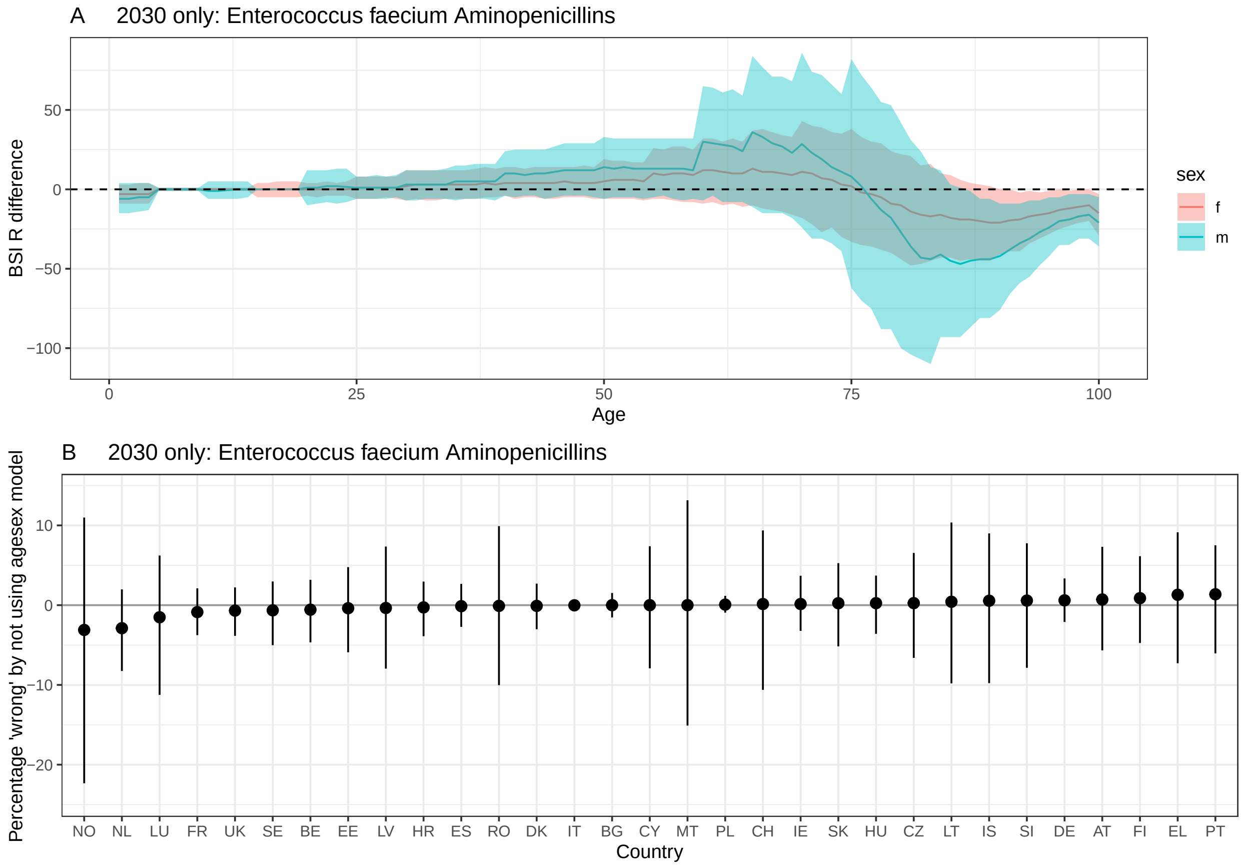

Figure A20: Difference between 'agesex' and 'base' model projections in 2030. A positive difference indicates that using the 'base' model underestimates resistant BSIs compared to the 'agesex' model. A) by age and sex. The line depicts the median and the ribbon the 95% quantiles. The dashed line at 0 indicates no difference. B) by country. The solid line indicates no difference, and the dotted / dashed lines represent a 50 and 100% difference respectively. The dot depicts the median and the ribbon the 95% quantiles. Enterococcus faecium Aminopenicillins.

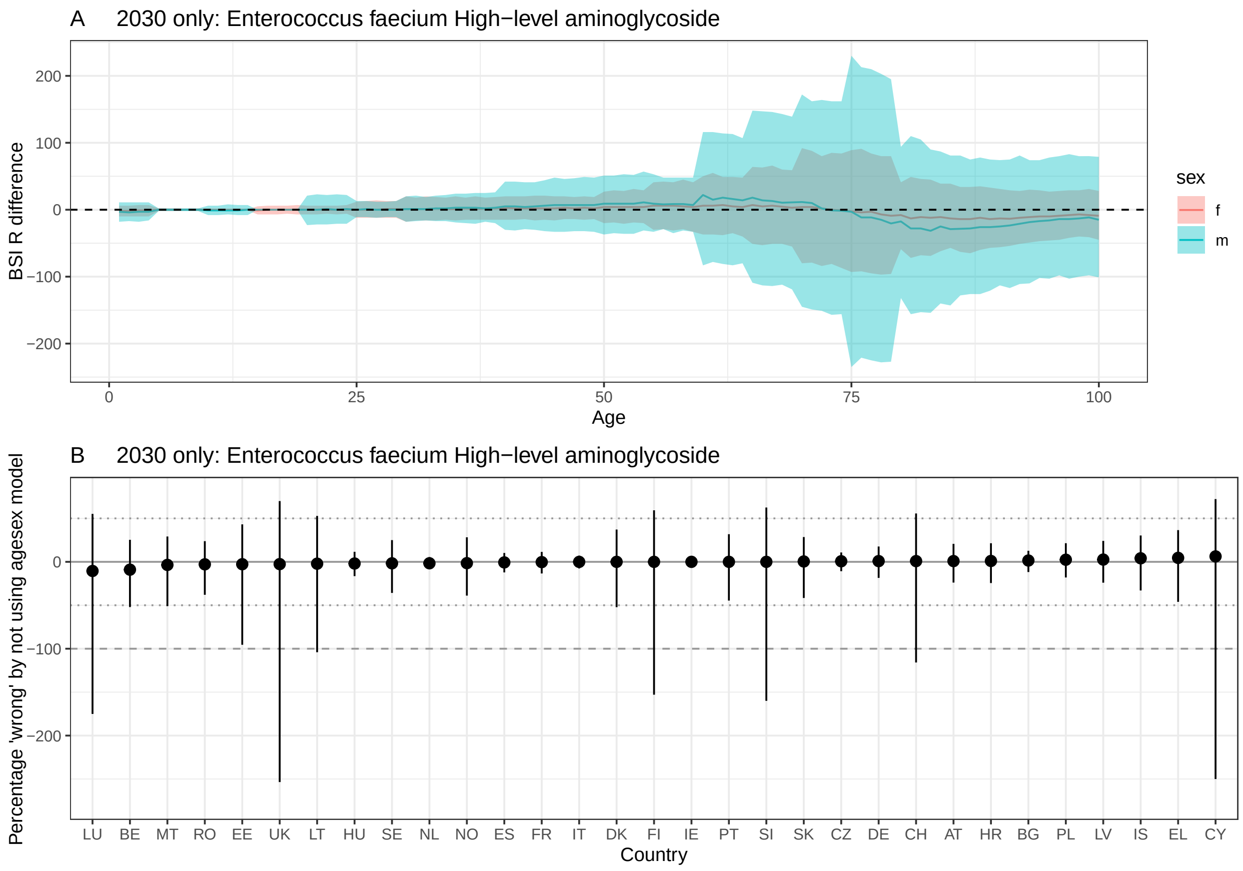

Figure A21: Difference between 'agesex' and 'base' model projections in 2030. A positive difference indicates that using the 'base' model underestimates resistant BSIs compared to the 'agesex' model. A) by age and sex. The line depicts the median and the ribbon the 95% quantiles. The dashed line at 0 indicates no difference. B) by country. The solid line indicates no difference, and the dotted / dashed lines represent a 50 and 100% difference respectively. The dot depicts the median and the ribbon the 95% quantiles. Enterococcus faecium High-level aminoglycoside .

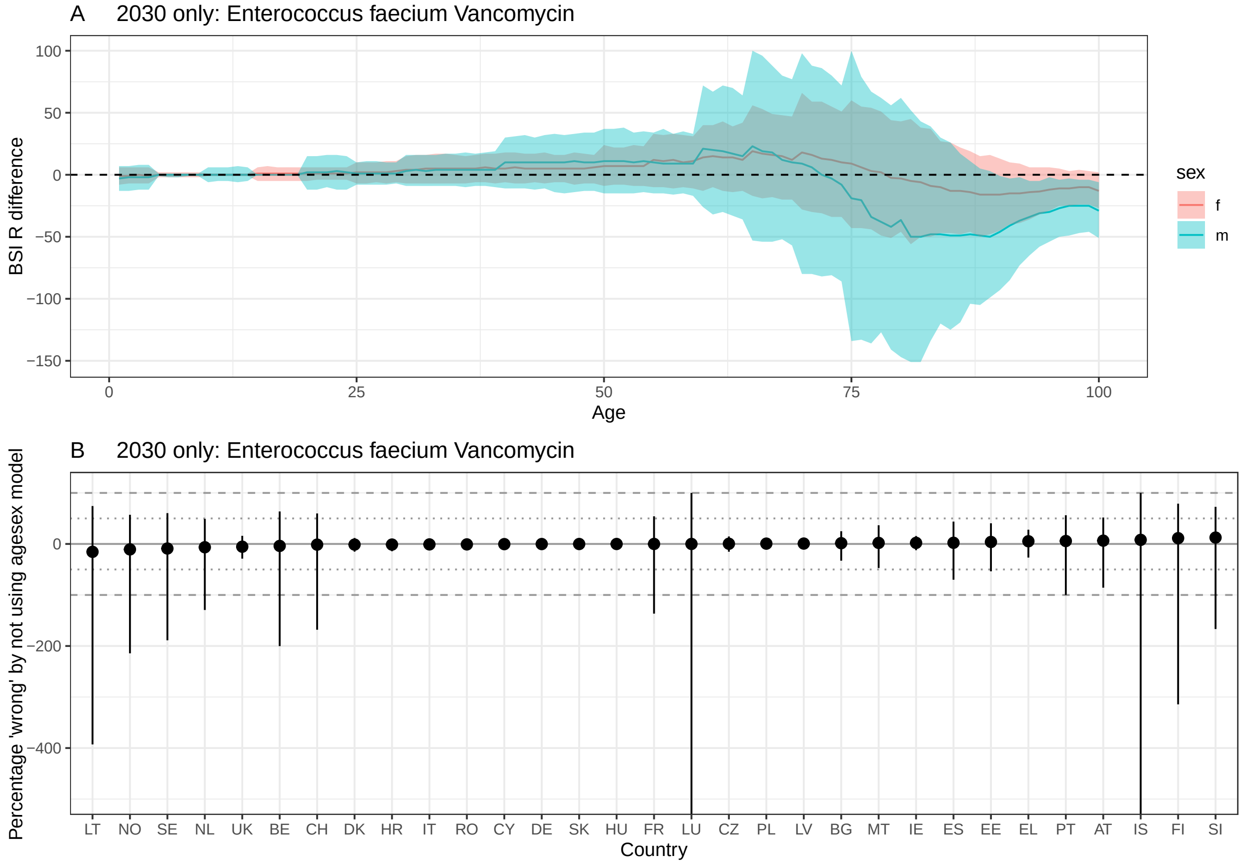

Figure A22: Difference between 'agesex' and 'base' model projections in 2030. A positive difference indicates that using the 'base' model underestimates resistant BSIs compared to the 'agesex' model. A) by age and sex. The line depicts the median and the ribbon the 95% quantiles. The dashed line at 0 indicates no difference. B) by country. The solid line indicates no difference, and the dotted / dashed lines represent a 50 and 100% difference respectively. The dot depicts the median and the ribbon the 95% quantiles. Enterococcus faecium Vancomycin.

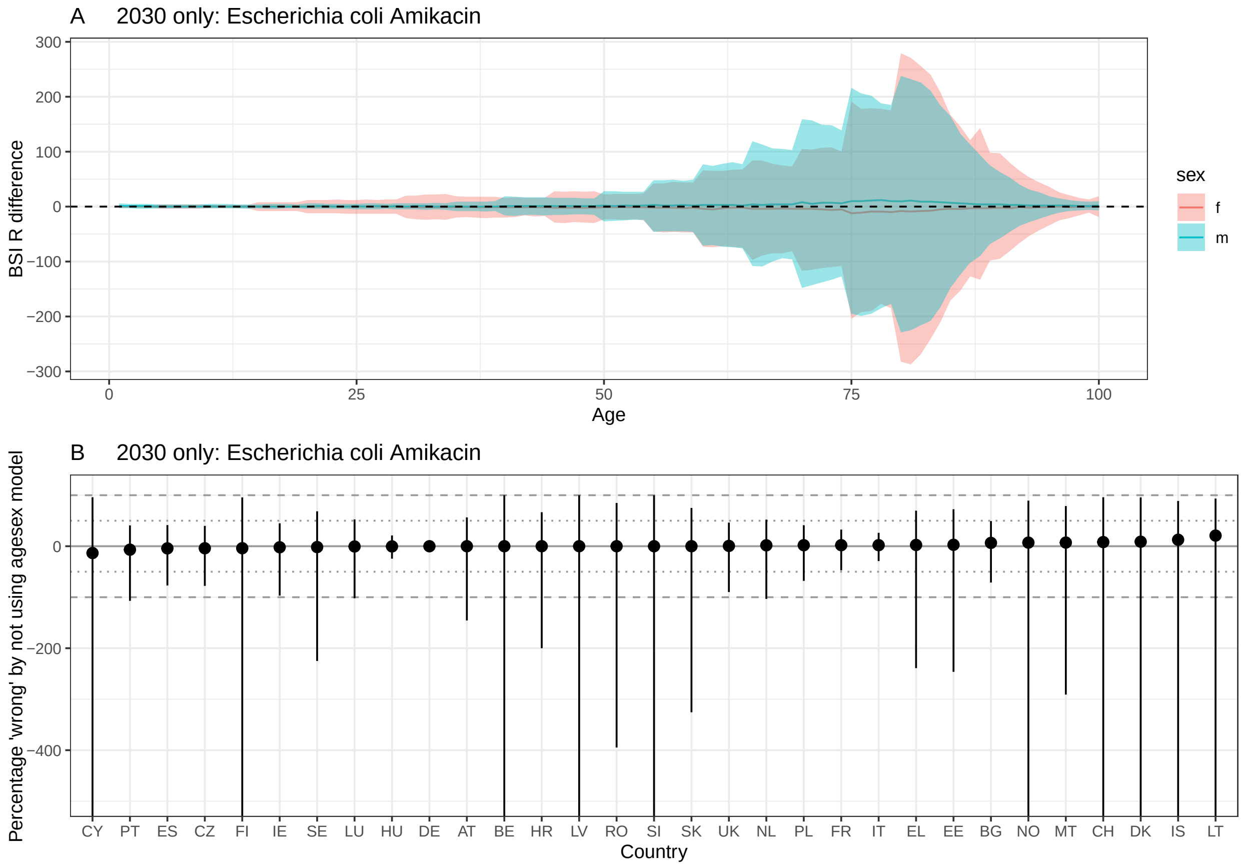

Figure A23: Difference between 'agesex' and 'base' model projections in 2030. A positive difference indicates that using the 'base' model underestimates resistant BSIs compared to the 'agesex' model. A) by age and sex. The line depicts the median and the ribbon the 95% quantiles. The dashed line at 0 indicates no difference. B) by country. The solid line indicates no difference, and the dotted / dashed lines represent a 50 and 100% difference respectively. The dot depicts the median and the ribbon the 95% quantiles. Escherichia coli Amikacin.

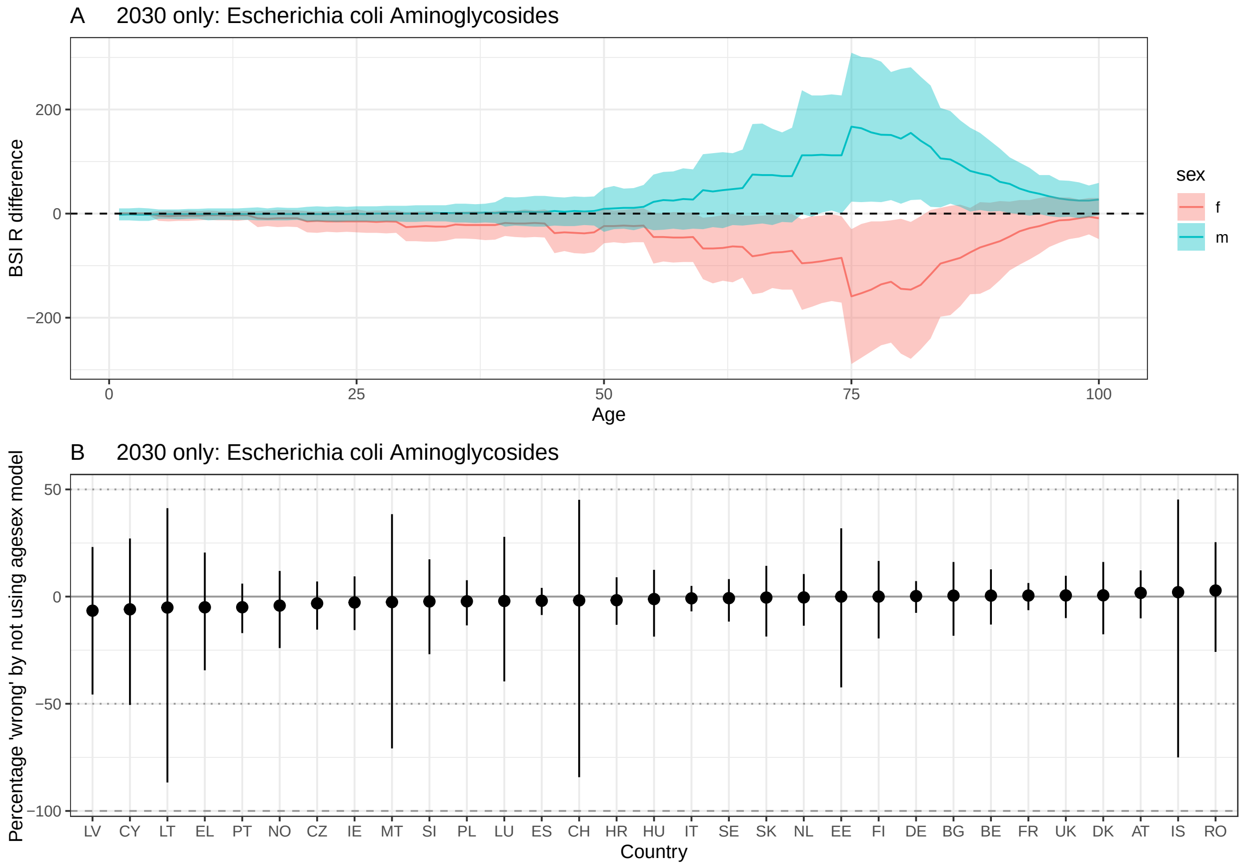

Figure A24: Difference between 'agesex' and 'base' model projections in 2030. A positive difference indicates that using the 'base' model underestimates resistant BSIs compared to the 'agesex' model. A) by age and sex. The line depicts the median and the ribbon the 95% quantiles. The dashed line at 0 indicates no difference. B) by country. The solid line indicates no difference, and the dotted / dashed lines represent a 50 and 100% difference respectively. The dot depicts the median and the ribbon the 95% quantiles. Escherichia coli Aminoglycosides.

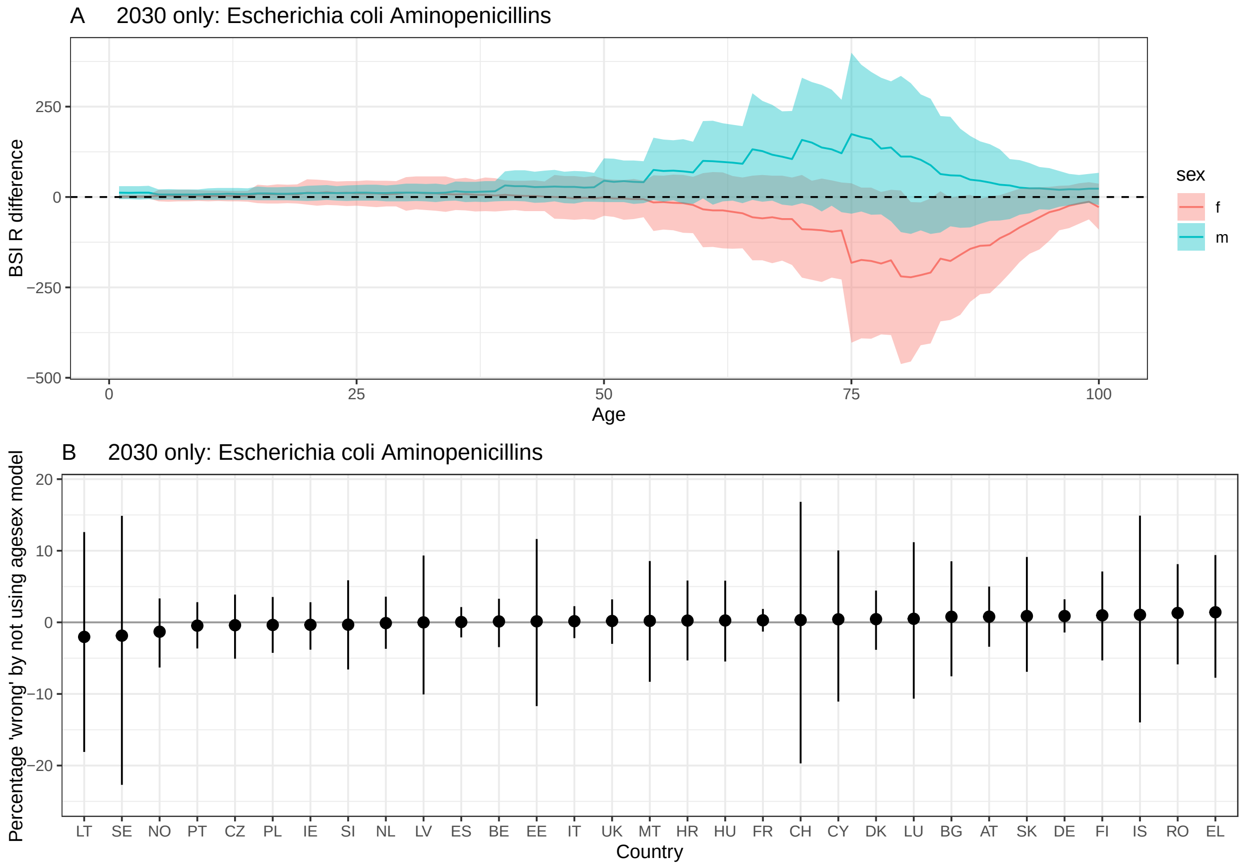

Figure A25: Difference between 'agesex' and 'base' model projections in 2030. A positive difference indicates that using the 'base' model underestimates resistant BSIs compared to the 'agesex' model. A) by age and sex. The line depicts the median and the ribbon the 95% quantiles. The dashed line at 0 indicates no difference. B) by country. The solid line indicates no difference, and the dotted / dashed lines represent a 50 and 100% difference respectively. The dot depicts the median and the ribbon the 95% quantiles. Escherichia coli Aminopenicillins.

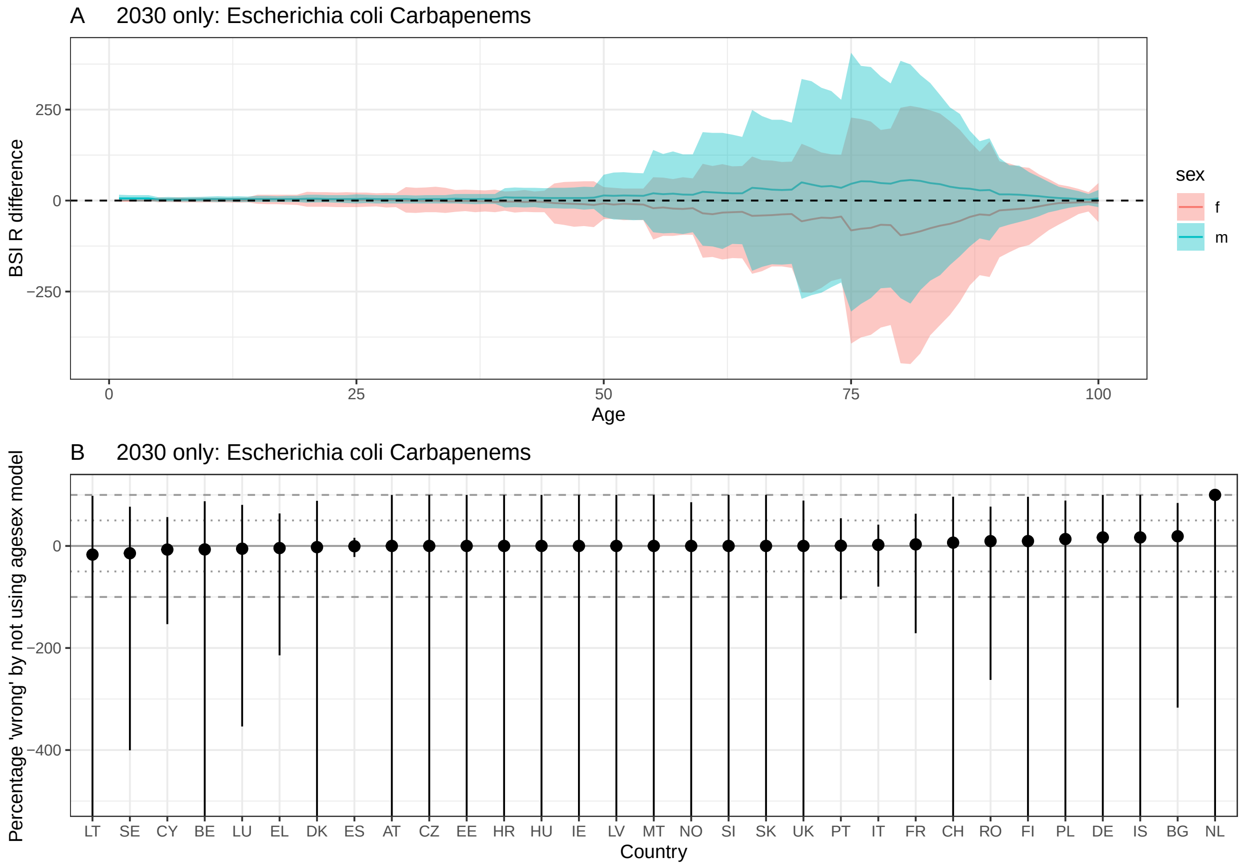

Figure A26: Difference between 'agesex' and 'base' model projections in 2030. A positive difference indicates that using the 'base' model underestimates resistant BSIs compared to the 'agesex' model. A) by age and sex. The line depicts the median and the ribbon the 95% quantiles. The dashed line at 0 indicates no difference. B) by country. The solid line indicates no difference, and the dotted / dashed lines represent a 50 and 100% difference respectively. The dot depicts the median and the ribbon the 95% quantiles. Escherichia coli Carbapenems.

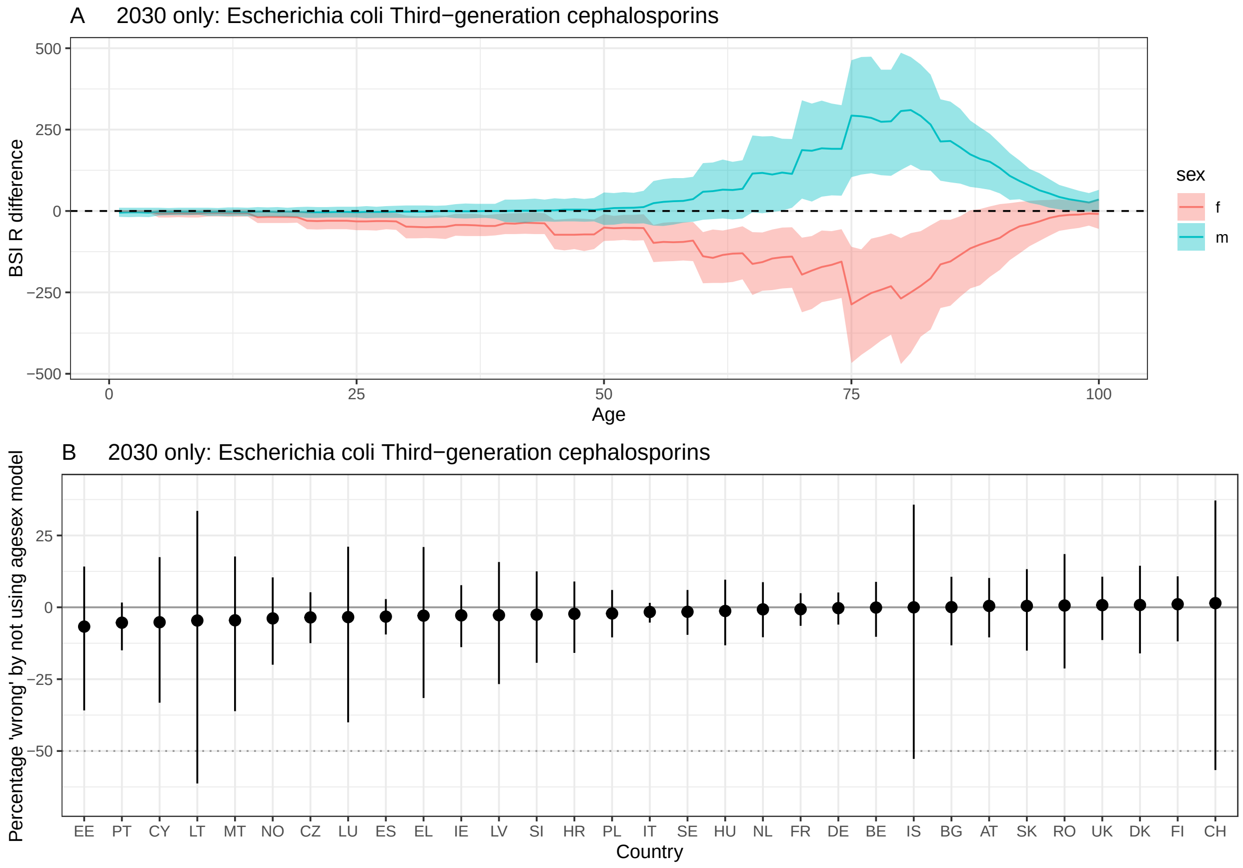

Figure A27: Difference between 'agesex' and 'base' model projections in 2030. A positive difference indicates that using the 'base' model underestimates resistant BSIs compared to the 'agesex' model. A) by age and sex. The line depicts the median and the ribbon the 95% quantiles. The dashed line at 0 indicates no difference. B) by country. The solid line indicates no difference, and the dotted / dashed lines represent a 50 and 100% difference respectively. The dot depicts the median and the ribbon the 95% quantiles. Escherichia coli Third-generation cephalosporins.

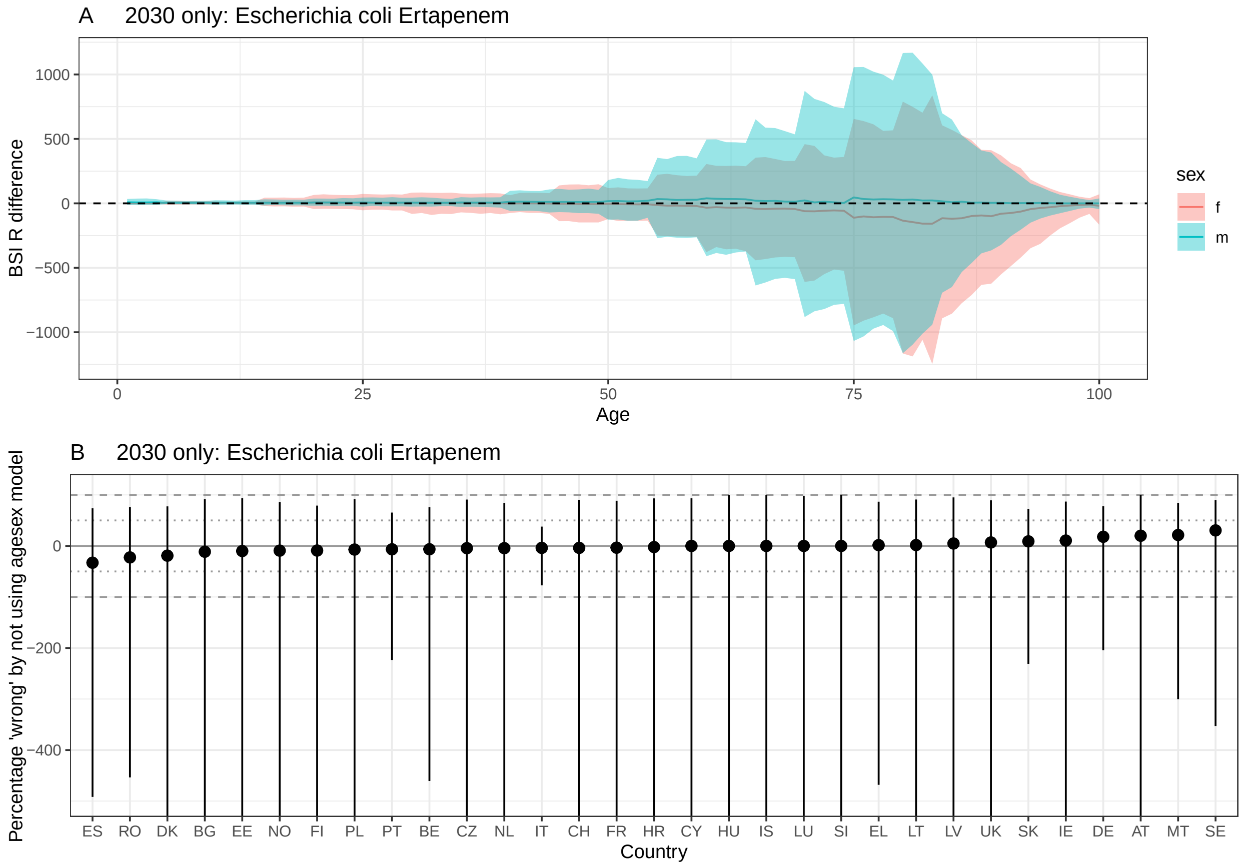

Figure A28: Difference between 'agesex' and 'base' model projections in 2030. A positive difference indicates that using the 'base' model underestimates resistant BSIs compared to the 'agesex' model. A) by age and sex. The line depicts the median and the ribbon the 95% quantiles. The dashed line at 0 indicates no difference. B) by country. The solid line indicates no difference, and the dotted / dashed lines represent a 50 and 100% difference respectively. The dot depicts the median and the ribbon the 95% quantiles. Escherichia coli Ertapenem.

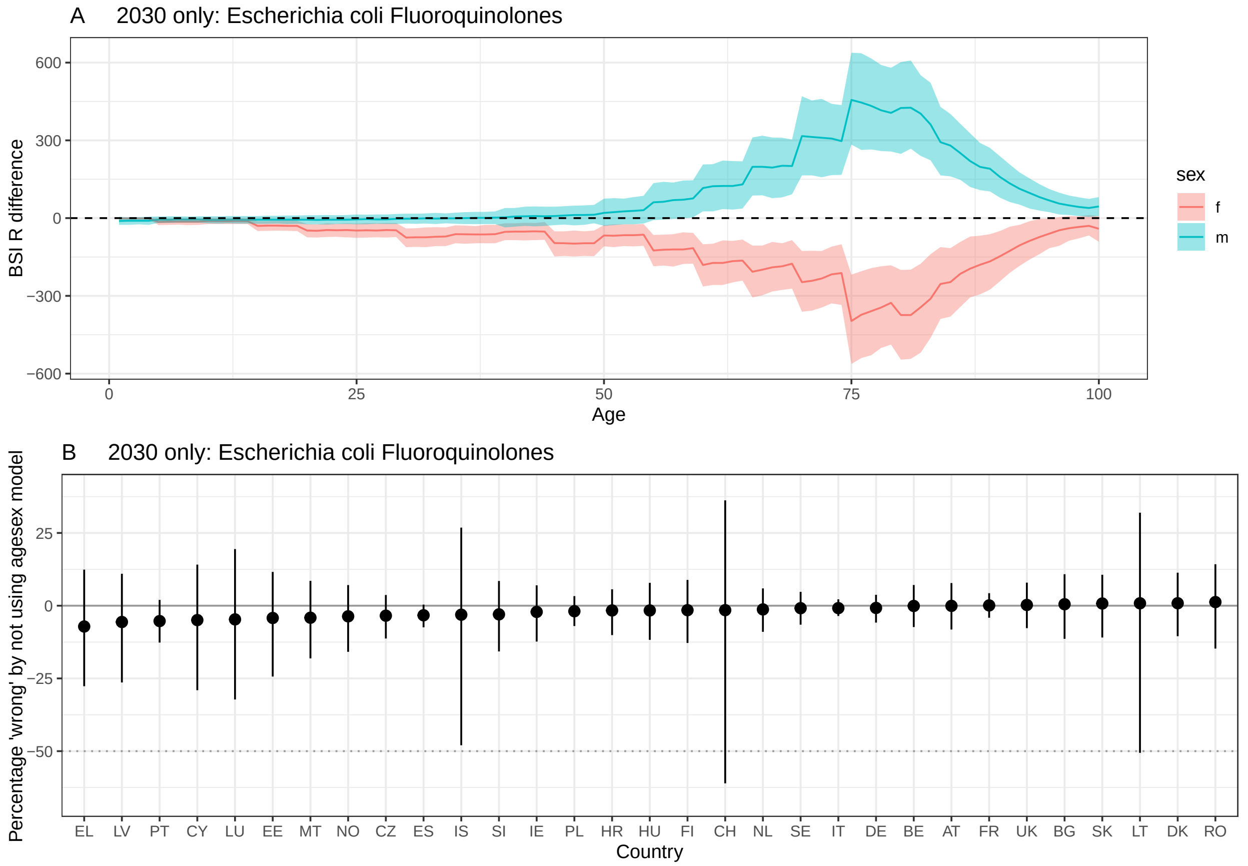

Figure A29: Difference between 'agesex' and 'base' model projections in 2030. A positive difference indicates that using the 'base' model underestimates resistant BSIs compared to the 'agesex' model. A) by age and sex. The line depicts the median and the ribbon the 95% quantiles. The dashed line at 0 indicates no difference. B) by country. The solid line indicates no difference, and the dotted / dashed lines represent a 50 and 100% difference respectively. The dot depicts the median and the ribbon the 95% quantiles. Escherichia coli Fluoroquinolones.

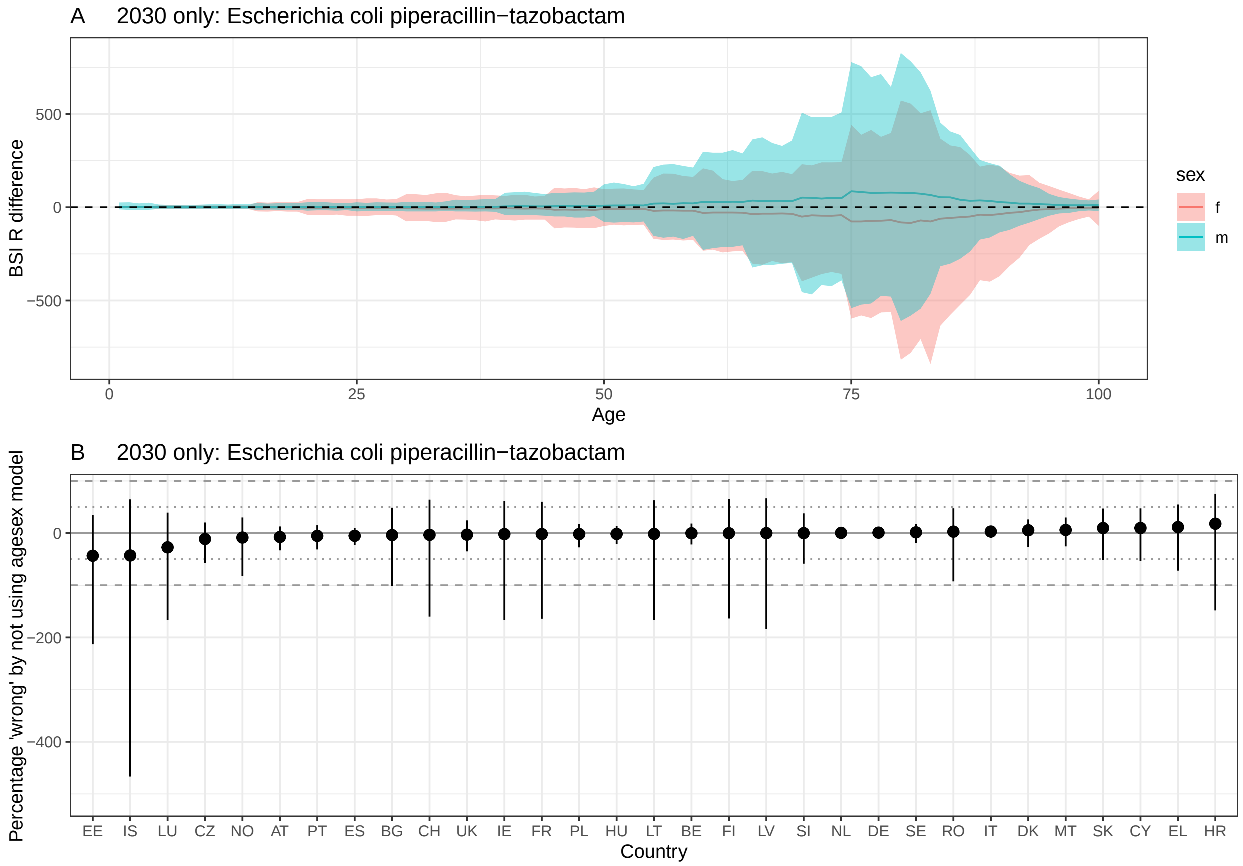

Figure A30: Difference between 'agesex' and 'base' model projections in 2030. A positive difference indicates that using the 'base' model underestimates resistant BSIs compared to the 'agesex' model. A) by age and sex. The line depicts the median and the ribbon the 95% quantiles. The dashed line at 0 indicates no difference. B) by country. The solid line indicates no difference, and the dotted / dashed lines represent a 50 and 100% difference respectively. The dot depicts the median and the ribbon the 95% quantiles. Escherichia coli piperacillin-tazobactam.

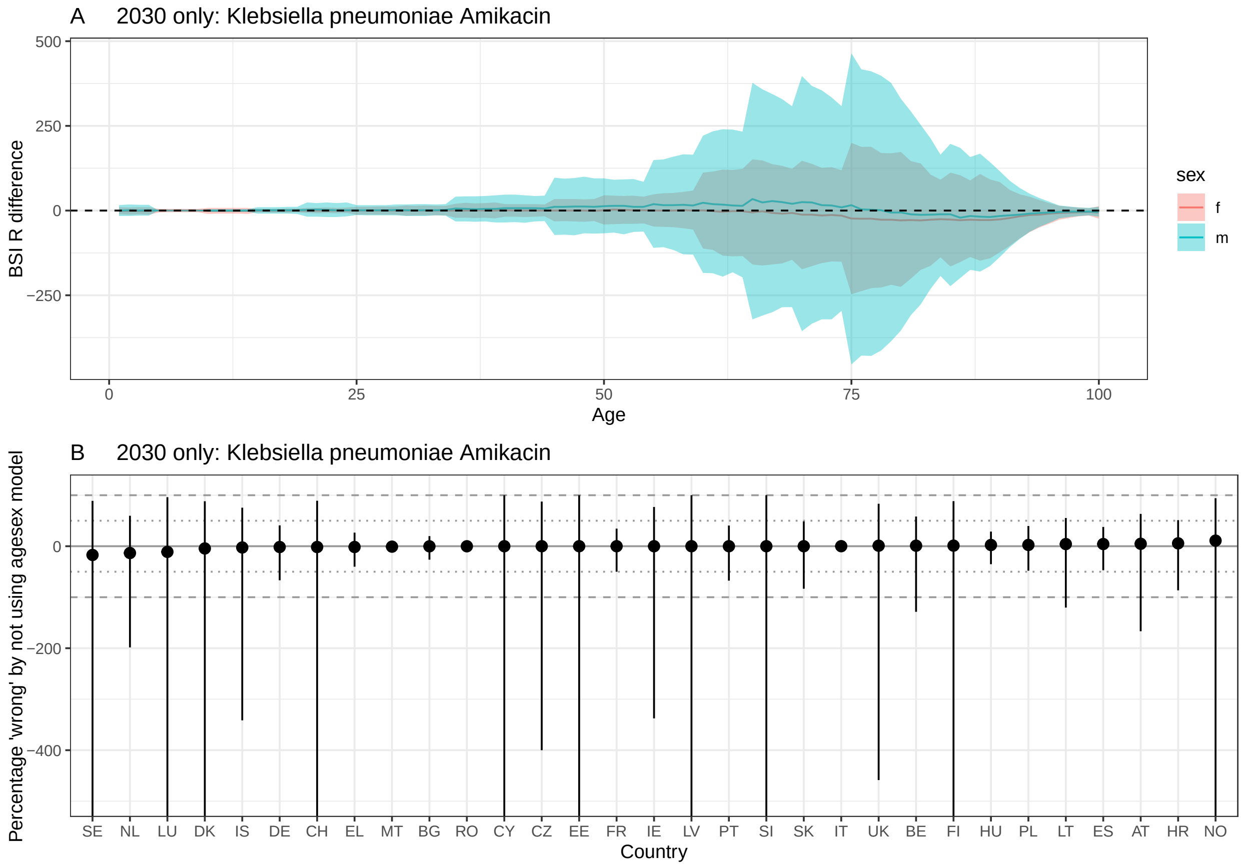

Figure A31: Difference between 'agesex' and 'base' model projections in 2030. A positive difference indicates that using the 'base' model underestimates resistant BSIs compared to the 'agesex' model. A) by age and sex. The line depicts the median and the ribbon the 95% quantiles. The dashed line at 0 indicates no difference. B) by country. The solid line indicates no difference, and the dotted / dashed lines represent a 50 and 100% difference respectively. The dot depicts the median and the ribbon the 95% quantiles. Klebsiella pneumoniae Amikacin.

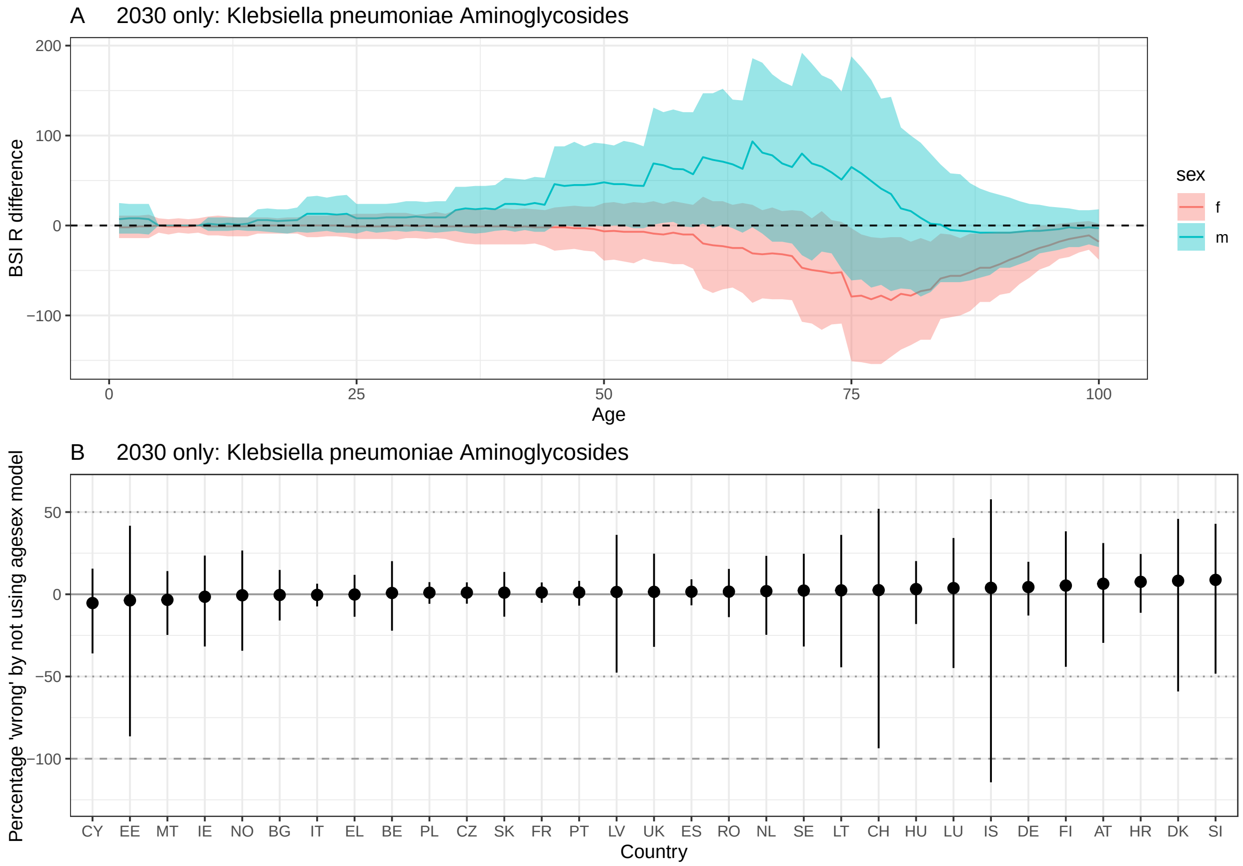

Figure A32: Difference between 'agesex' and 'base' model projections in 2030. A positive difference indicates that using the 'base' model underestimates resistant BSIs compared to the 'agesex' model. A) by age and sex. The line depicts the median and the ribbon the 95% quantiles. The dashed line at 0 indicates no difference. B) by country. The solid line indicates no difference, and the dotted / dashed lines represent a 50 and 100% difference respectively. The dot depicts the median and the ribbon the 95% quantiles. Klebsiella pneumoniae Aminoglycosides.

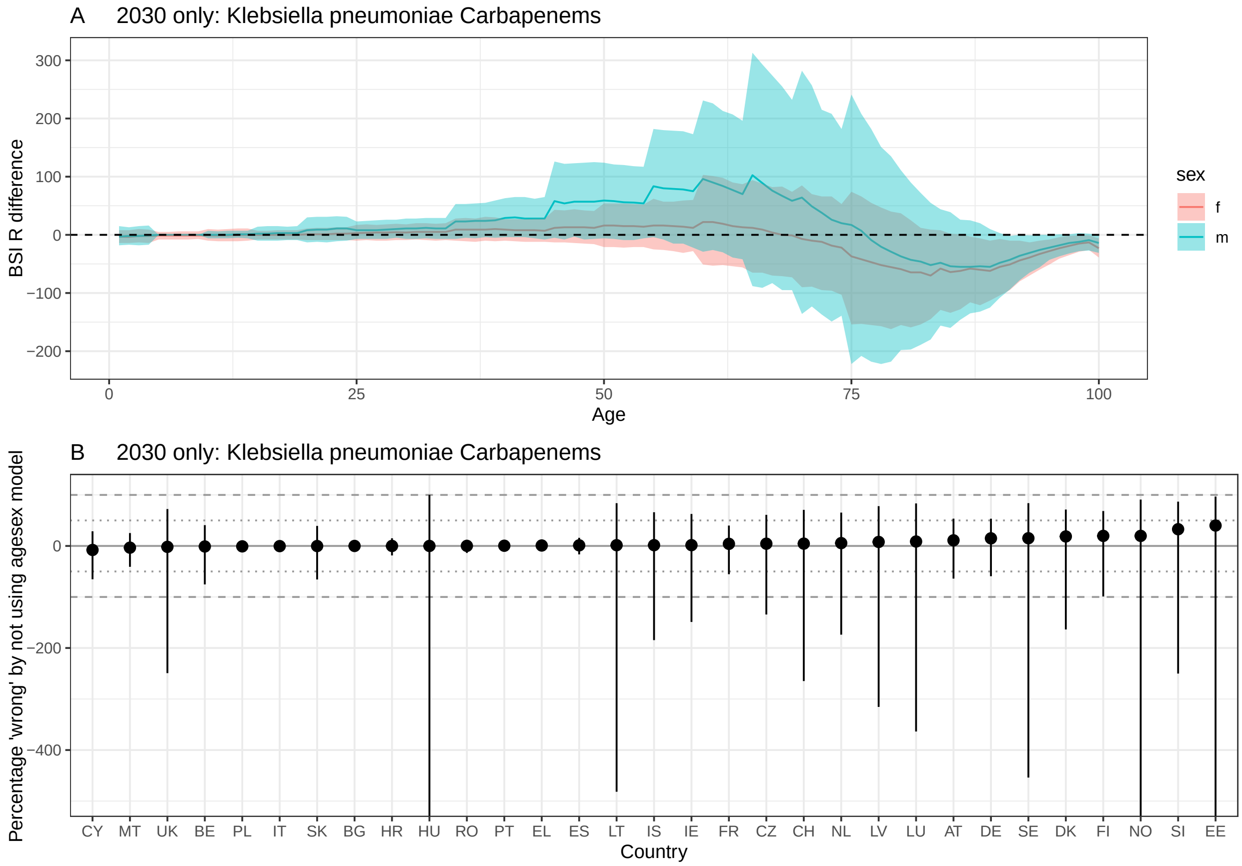

Figure A33: Difference between 'agesex' and 'base' model projections in 2030. A positive difference indicates that using the 'base' model underestimates resistant BSIs compared to the 'agesex' model. A) by age and sex. The line depicts the median and the ribbon the 95% quantiles. The dashed line at 0 indicates no difference. B) by country. The solid line indicates no difference, and the dotted / dashed lines represent a 50 and 100% difference respectively. The dot depicts the median and the ribbon the 95% quantiles. Klebsiella pneumoniae Carbapenems.

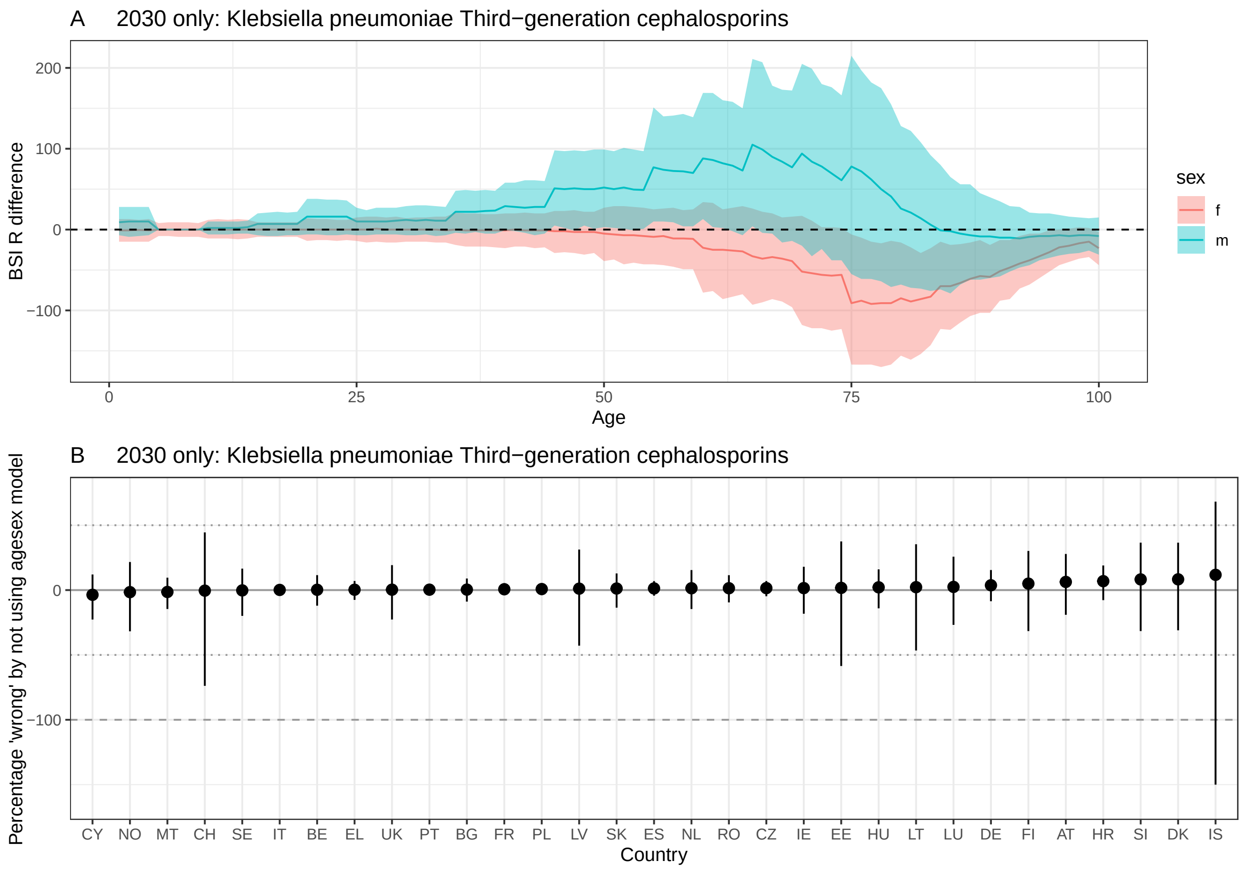

Figure A34: Difference between 'agesex' and 'base' model projections in 2030. A positive difference indicates that using the 'base' model underestimates resistant BSIs compared to the 'agesex' model. A) by age and sex. The line depicts the median and the ribbon the 95% quantiles. The dashed line at 0 indicates no difference. B) by country. The solid line indicates no difference, and the dotted / dashed lines represent a 50 and 100% difference respectively. The dot depicts the median and the ribbon the 95% quantiles. Klebsiella pneumoniae Third-generation cephalosporins.

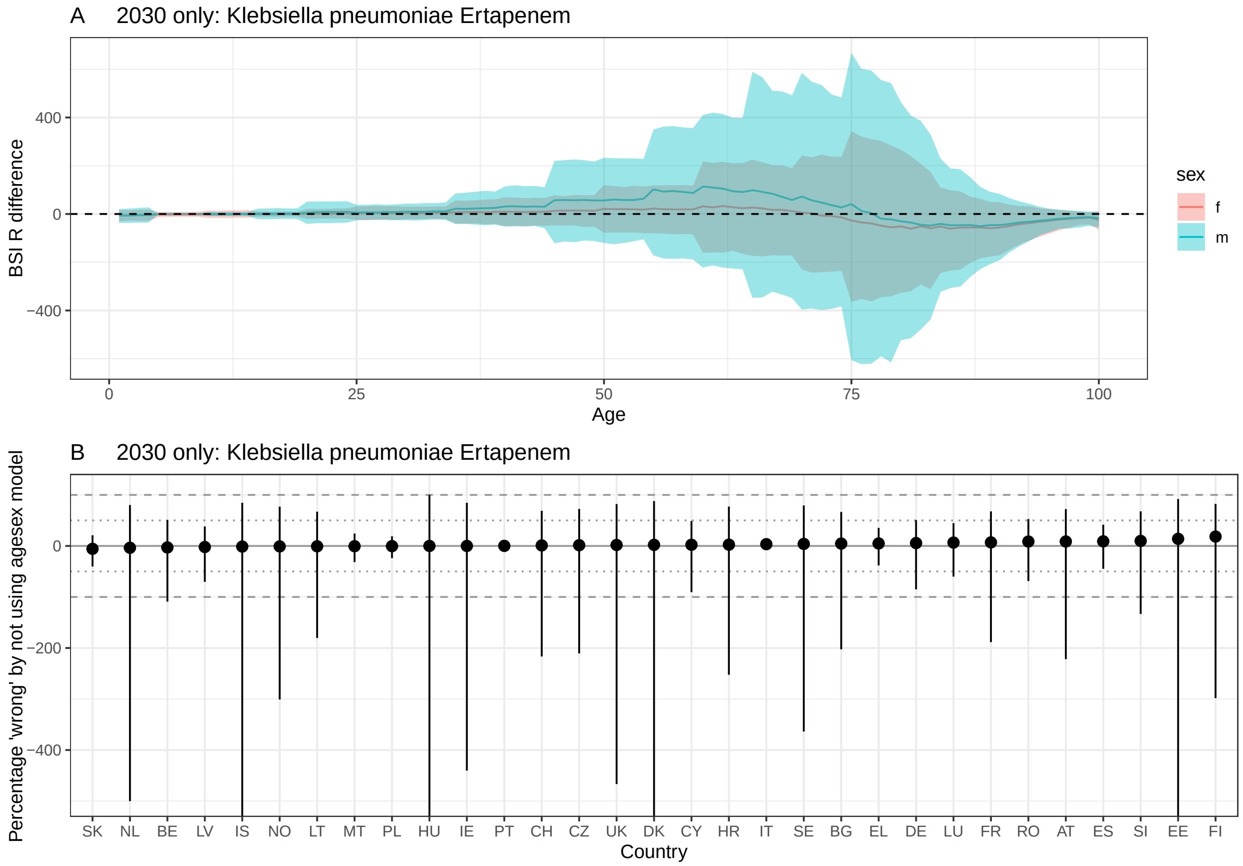

Figure A35: Difference between 'agesex' and 'base' model projections in 2030. A positive difference indicates that using the 'base' model underestimates resistant BSIs compared to the 'agesex' model. A) by age and sex. The line depicts the median and the ribbon the 95% quantiles. The dashed line at 0 indicates no difference. B) by country. The solid line indicates no difference, and the dotted / dashed lines represent a 50 and 100% difference respectively. The dot depicts the median and the ribbon the 95% quantiles. Klebsiella pneumoniae Ertapenem.

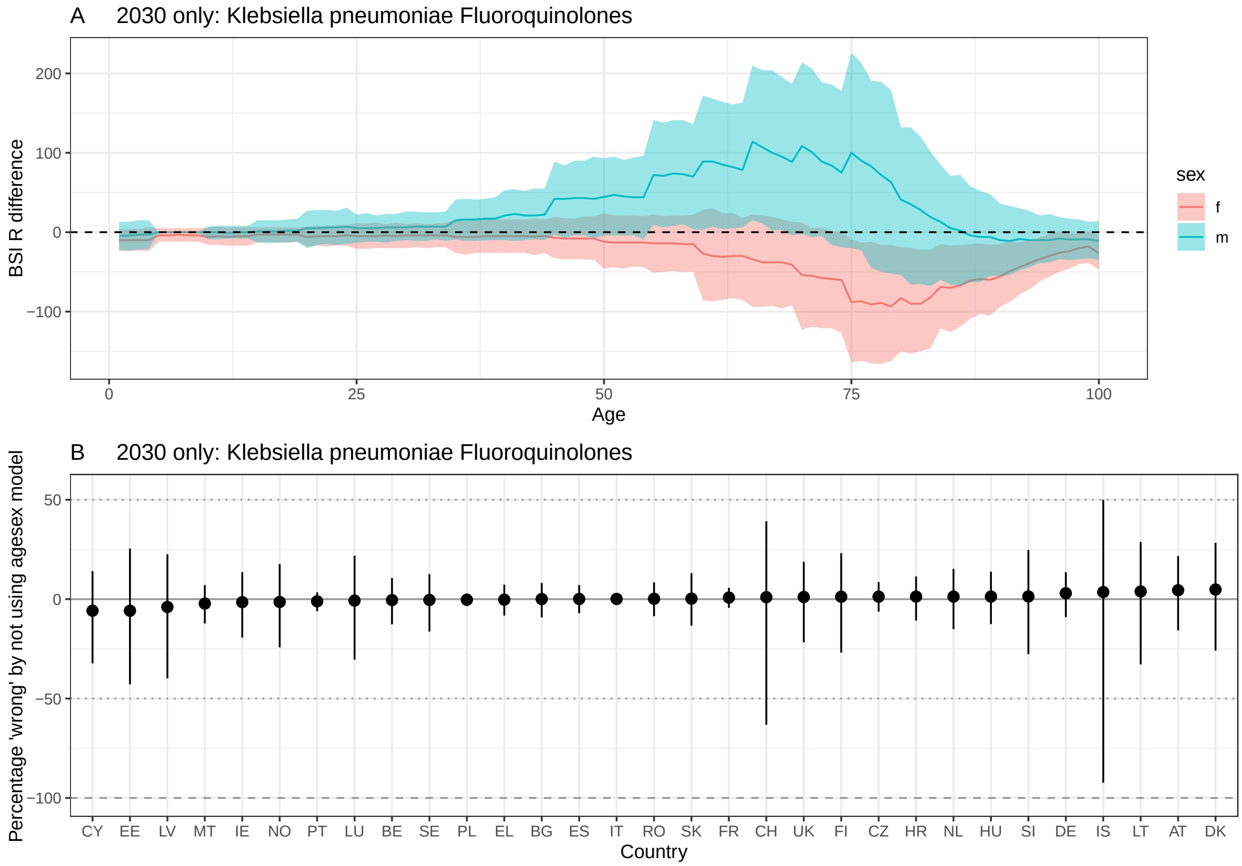

Figure A36: Difference between 'agesex' and 'base' model projections in 2030. A positive difference indicates that using the 'base' model underestimates resistant BSIs compared to the 'agesex' model. A) by age and sex. The line depicts the median and the ribbon the 95% quantiles. The dashed line at 0 indicates no difference. B) by country. The solid line indicates no difference, and the dotted / dashed lines represent a 50 and 100% difference respectively. The dot depicts the median and the ribbon the 95% quantiles. Klebsiella pneumoniae Fluoroquinolones.

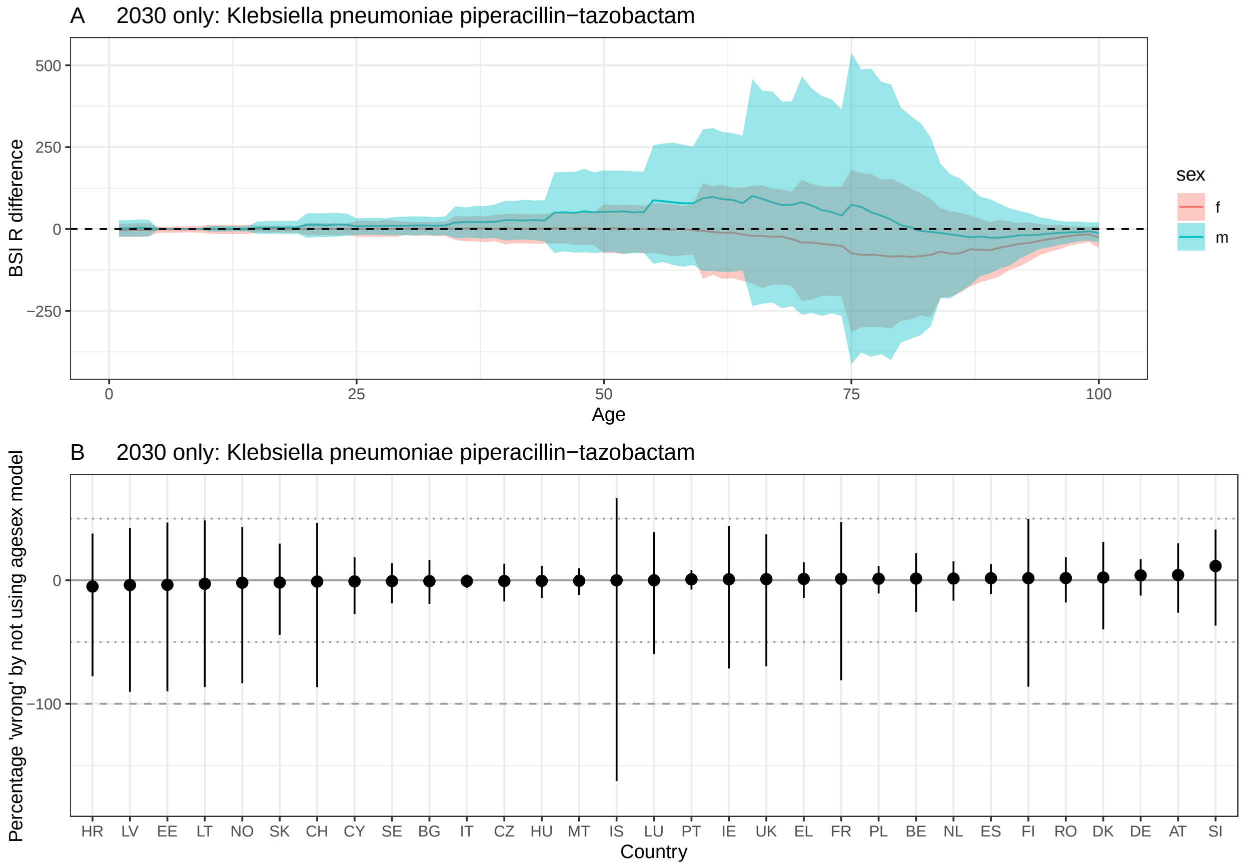

Figure A37: Difference between 'agesex' and 'base' model projections in 2030. A positive difference indicates that using the 'base' model underestimates resistant BSIs compared to the 'agesex' model. A) by age and sex. The line depicts the median and the ribbon the 95% quantiles. The dashed line at 0 indicates no difference. B) by country. The solid line indicates no difference, and the dotted / dashed lines represent a 50 and 100% difference respectively. The dot depicts the median and the ribbon the 95% quantiles. Klebsiella pneumoniae piperacillin-tazobactam.

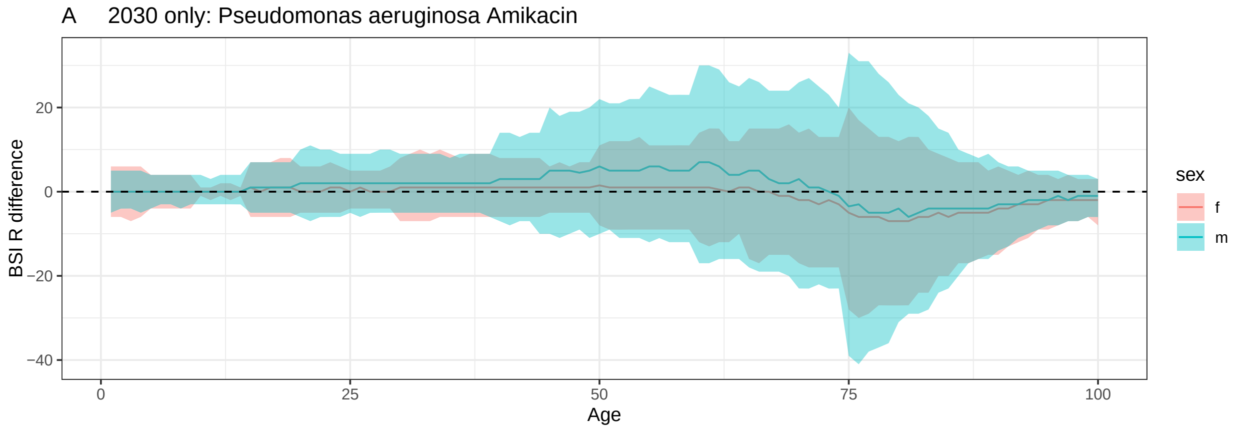

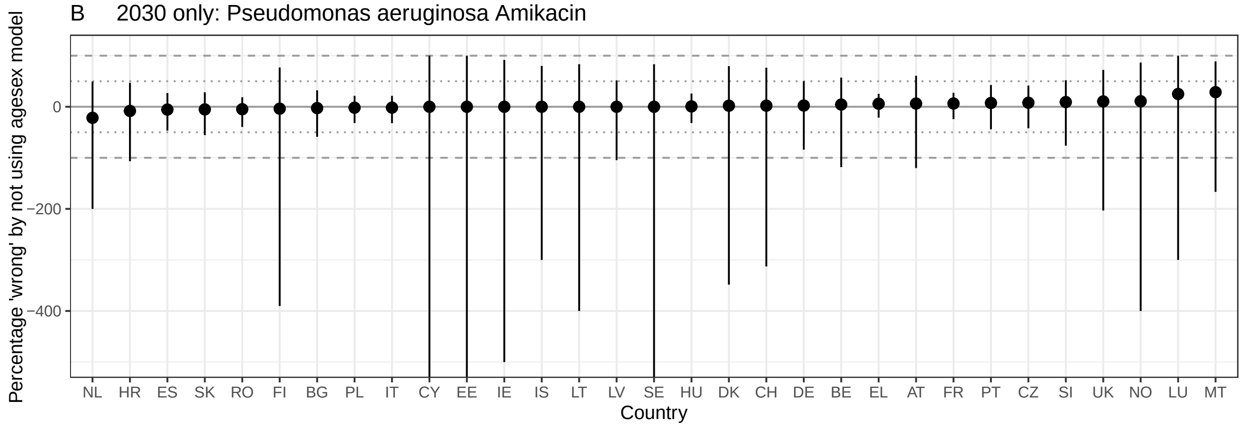

Figure A38: Difference between 'agesex' and 'base' model projections in 2030. A positive difference indicates that using the 'base' model underestimates resistant BSIs compared to the 'agesex' model. A) by age and sex. The line depicts the median and the ribbon the 95% quantiles. The dashed line at 0 indicates no difference. B) by country. The solid line indicates no difference, and the dotted / dashed lines represent a 50 and 100% difference respectively. The dot depicts the median and the ribbon the 95% quantiles. Pseudomonas aeruginosa Amikacin.

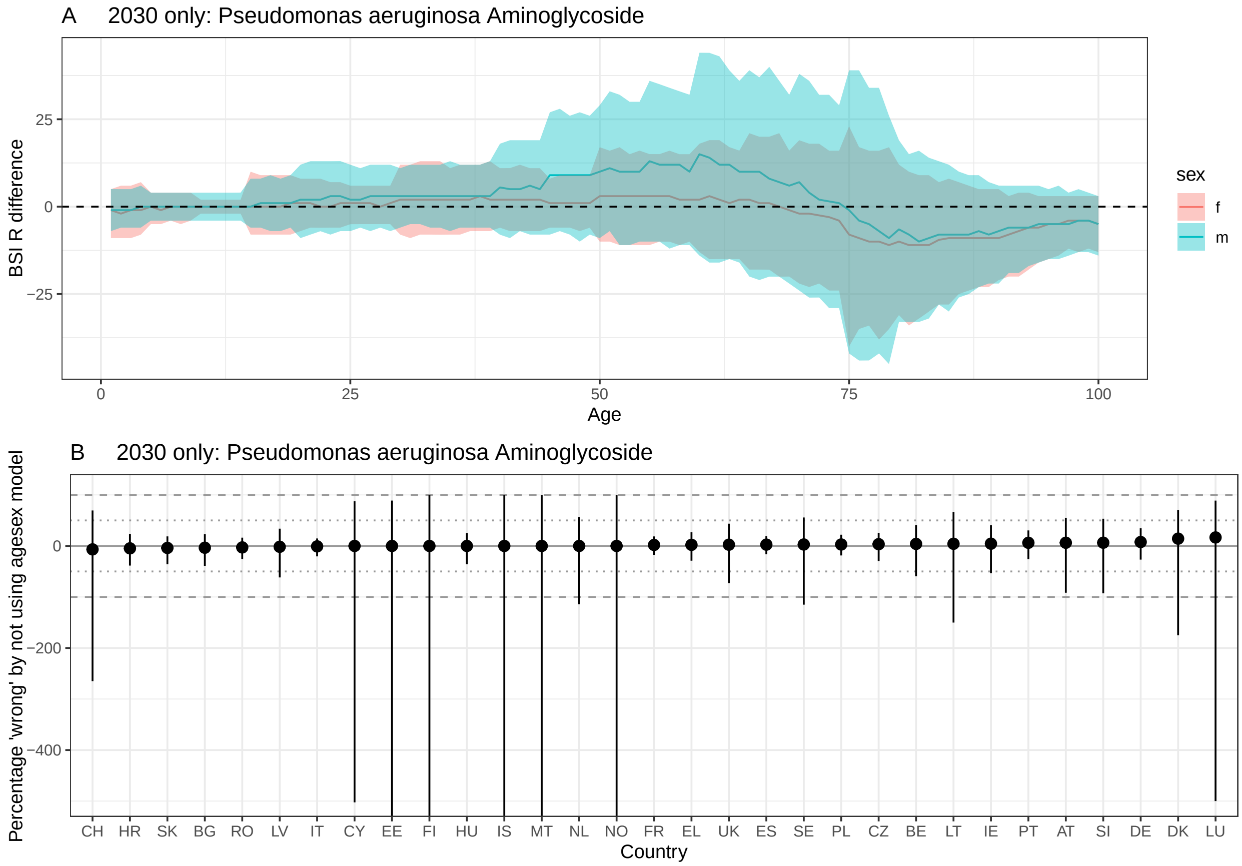

Figure A39: Difference between ‘agesex’ and ‘base’ model projections in 2030. A positive difference indicates that using the ‘base’ model underestimates resistant BSIs compared to the ‘agesex’ model. A) by age and sex. The line depicts the median and the ribbon the 95% quantiles. The dashed line at 0 indicates no difference. B) by country. The solid line indicates no difference, and the dotted / dashed lines represent a 50 and 100% difference respectively. The dot depicts the median and the ribbon the 95% quantiles. Pseudomonas aeruginosa Aminoglycoside.

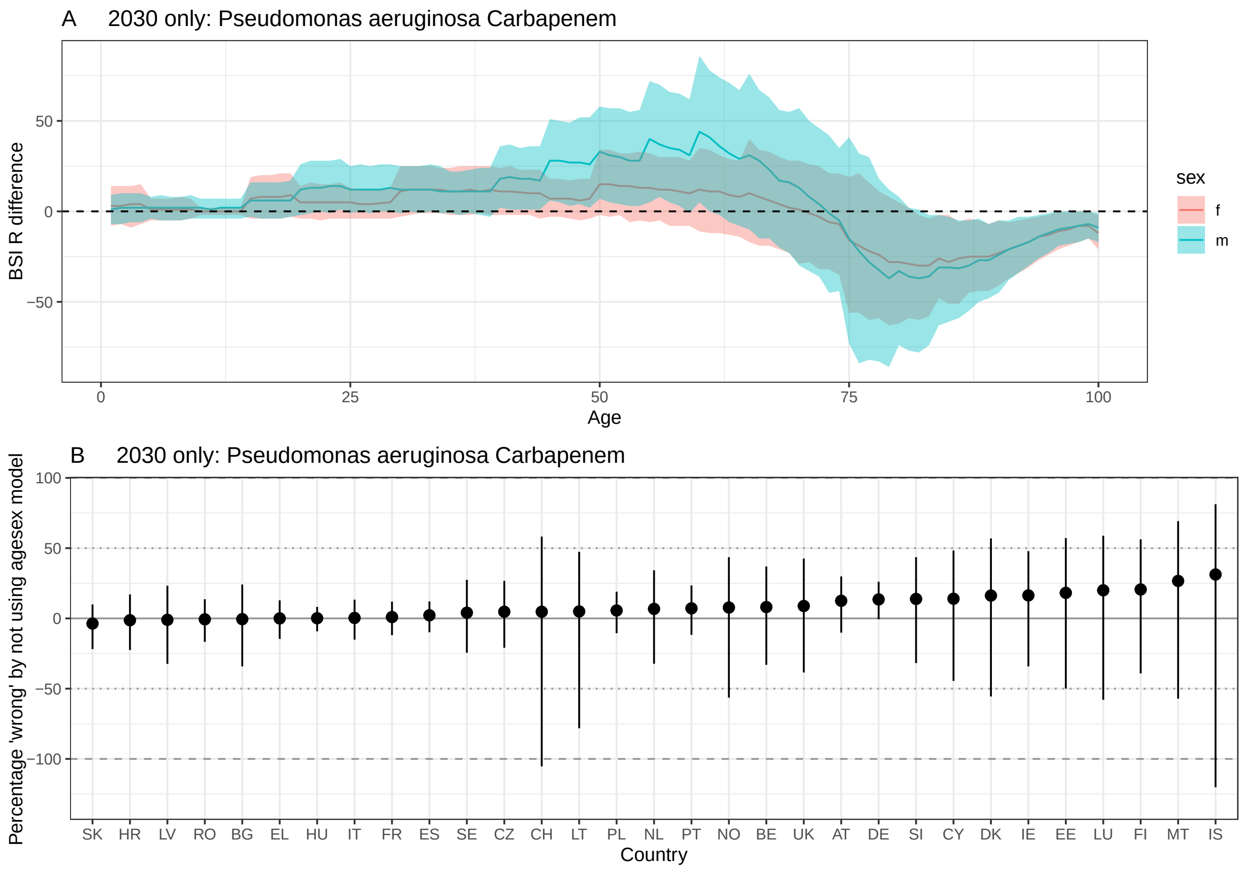

Figure A40: Difference between 'agesex' and 'base' model projections in 2030. A positive difference indicates that using the 'base' model underestimates resistant BSIs compared to the 'agesex' model. A) by age and sex. The line depicts the median and the ribbon the 95% quantiles. The dashed line at 0 indicates no difference. B) by country. The solid line indicates no difference, and the dotted / dashed lines represent a 50 and 100% difference respectively. The dot depicts the median and the ribbon the 95% quantiles. Pseudomonas aeruginosa Carbapenem.

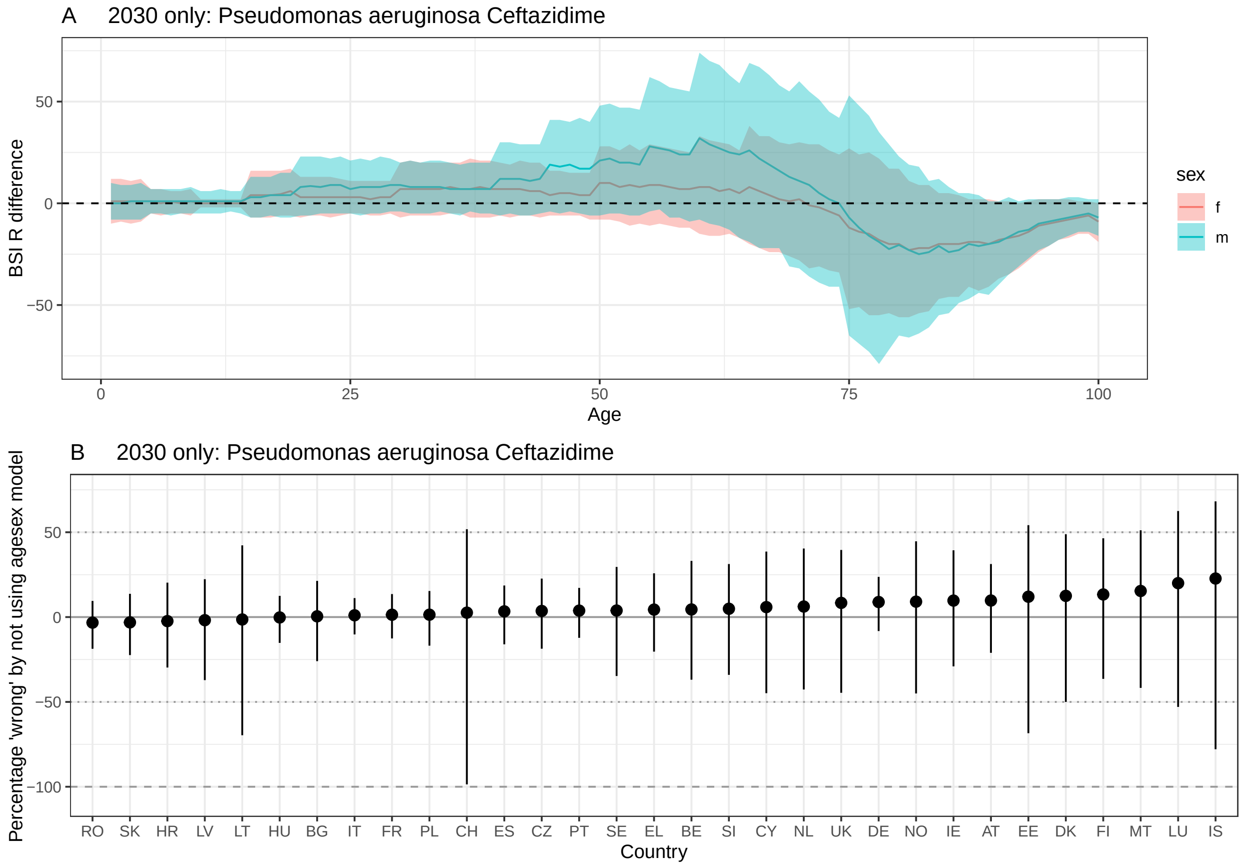

Figure A41: Difference between 'agesex' and 'base' model projections in 2030. A positive difference indicates that using the 'base' model underestimates resistant BSIs compared to the 'agesex' model. A) by age and sex. The line depicts the median and the ribbon the 95% quantiles. The dashed line at 0 indicates no difference. B) by country. The solid line indicates no difference, and the dotted / dashed lines represent a 50 and 100% difference respectively. The dot depicts the median and the ribbon the 95% quantiles. Pseudomonas aeruginosa Ceftazidime.

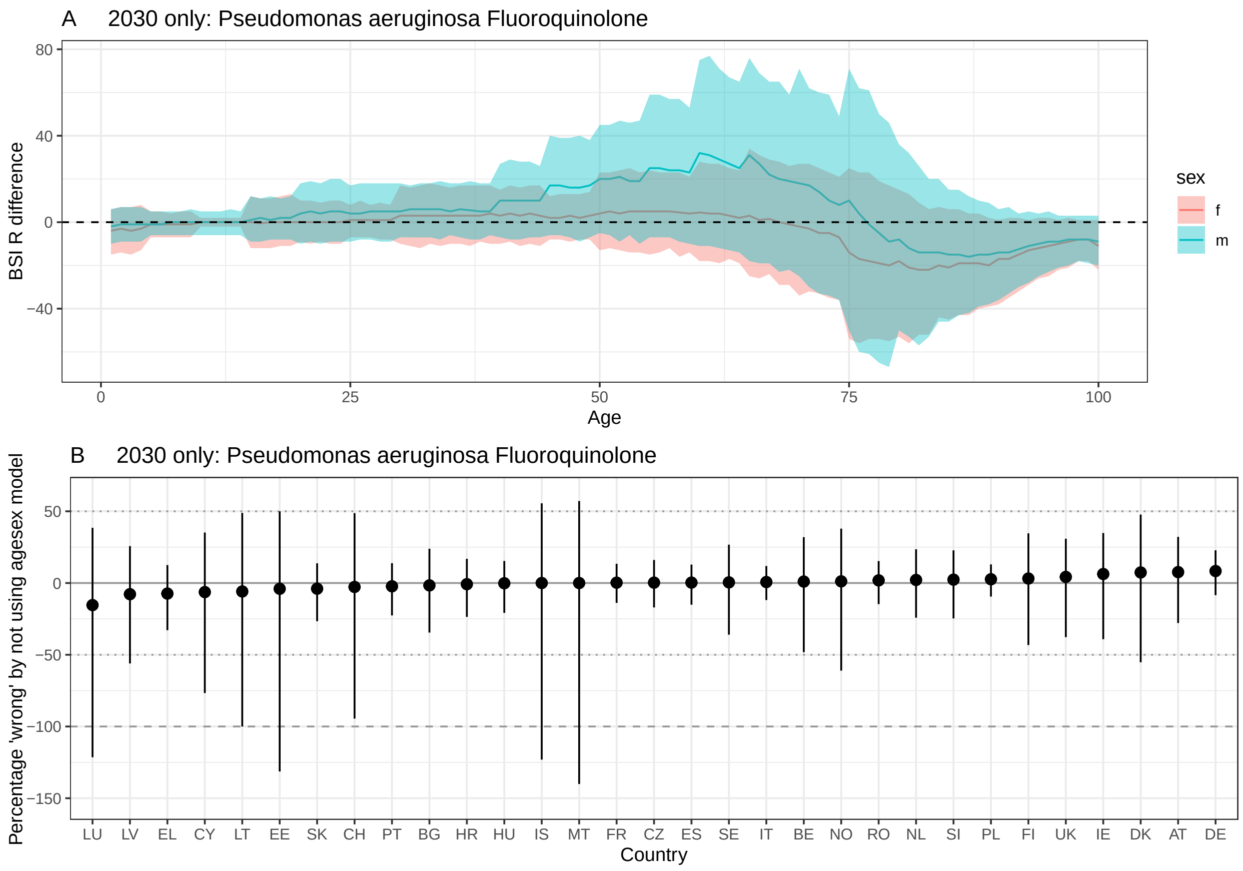

Figure A42: Difference between 'agesex' and 'base' model projections in 2030. A positive difference indicates that using the 'base' model underestimates resistant BSIs compared to the 'agesex' model. A) by age and sex. The line depicts the median and the ribbon the 95% quantiles. The dashed line at 0 indicates no difference. B) by country. The solid line indicates no difference, and the dotted / dashed lines represent a 50 and 100% difference respectively. The dot depicts the median and the ribbon the 95% quantiles. Pseudomonas aeruginosa Fluoroquinolone.

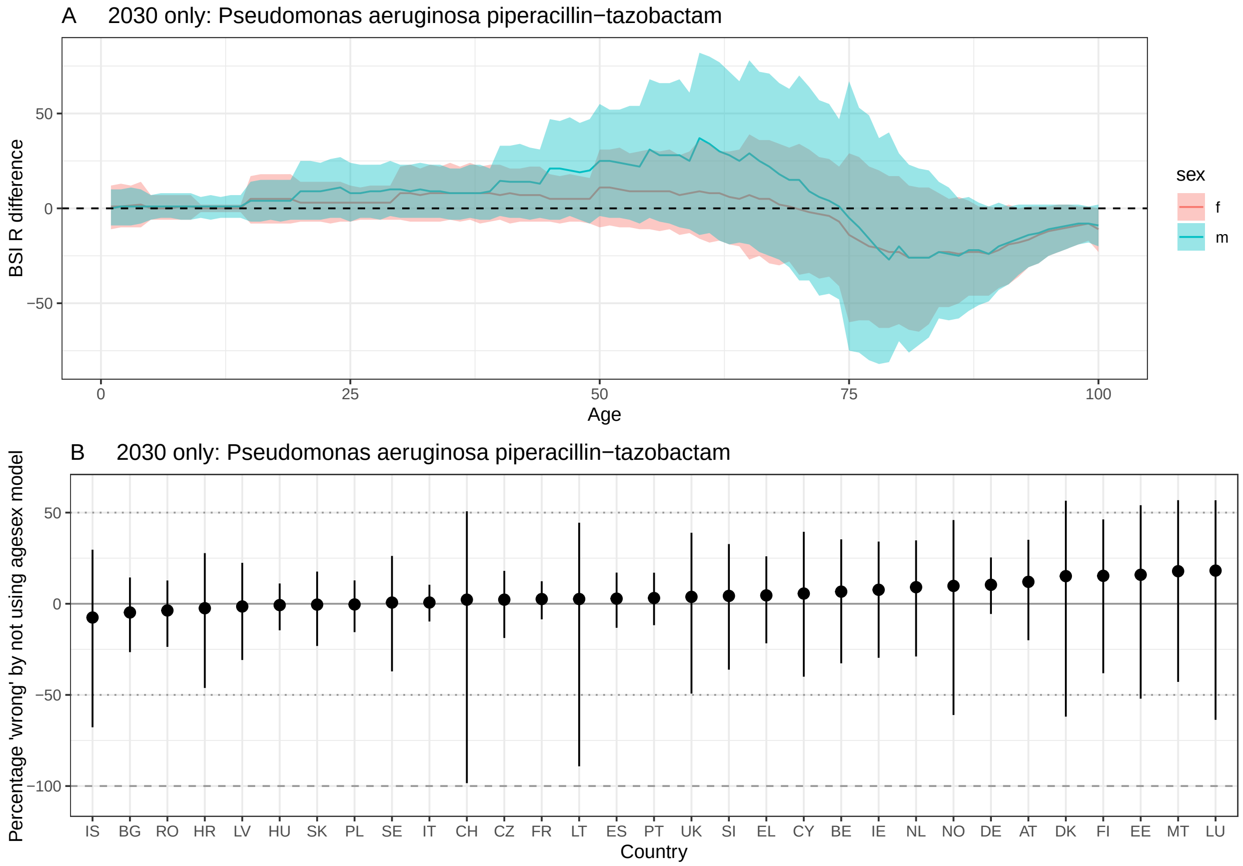

Figure A43: Difference between 'agesex' and 'base' model projections in 2030. A positive difference indicates that using the 'base' model underestimates resistant BSIs compared to the 'agesex' model. A) by age and sex. The line depicts the median and the ribbon the 95% quantiles. The dashed line at 0 indicates no difference. B) by country. The solid line indicates no difference, and the dotted / dashed lines represent a 50 and 100% difference respectively. The dot depicts the median and the ribbon the 95% quantiles. Pseudomonas aeruginosa piperacillin-tazobactam.

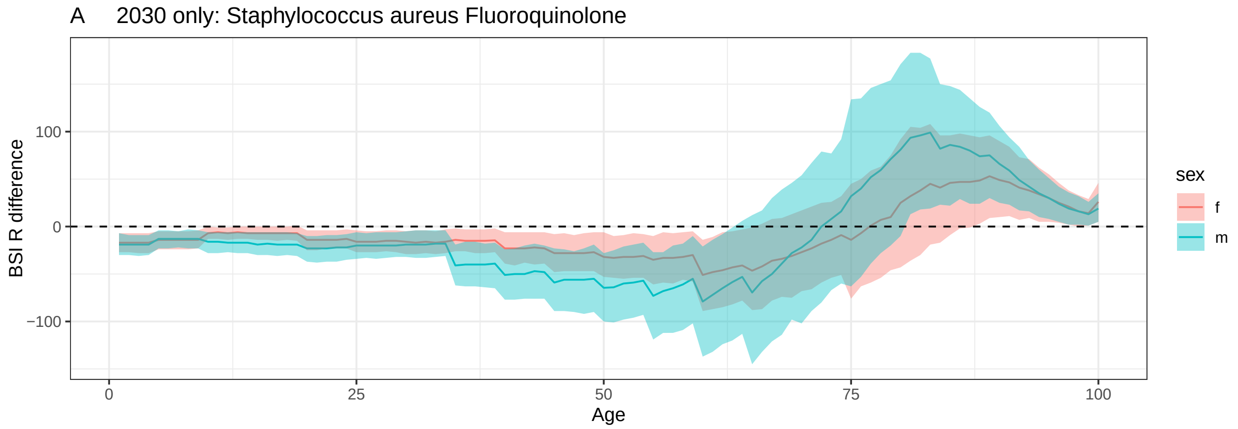

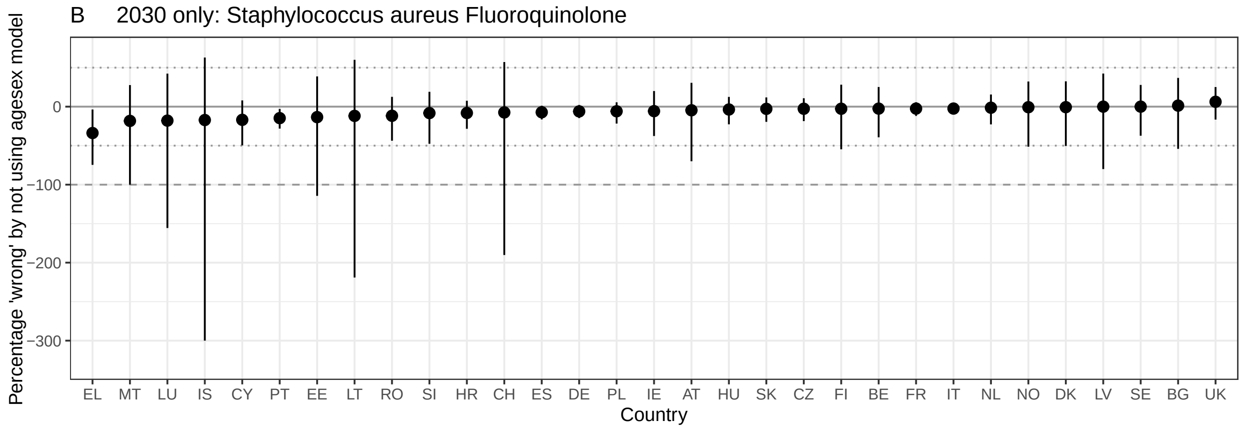

Figure A44: Difference between ‘agesex’ and ‘base’ model projections in 2030. A positive difference indicates that using the ‘base’ model underestimates resistant BSIs compared to the ‘agesex’ model. A) by age and sex. The line depicts the median and the ribbon the 95% quantiles. The dashed line at 0 indicates no difference. B) by country. The solid line indicates no difference, and the dotted / dashed lines represent a 50 and 100% difference respectively. The dot depicts the median and the ribbon the 95% quantiles. Staphylococcus aureus Fluoroquinolone.

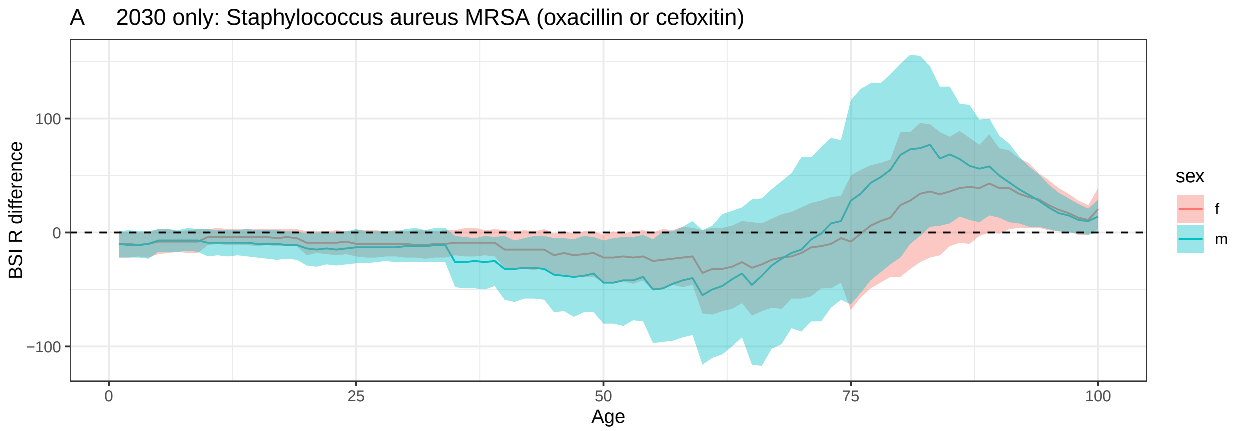

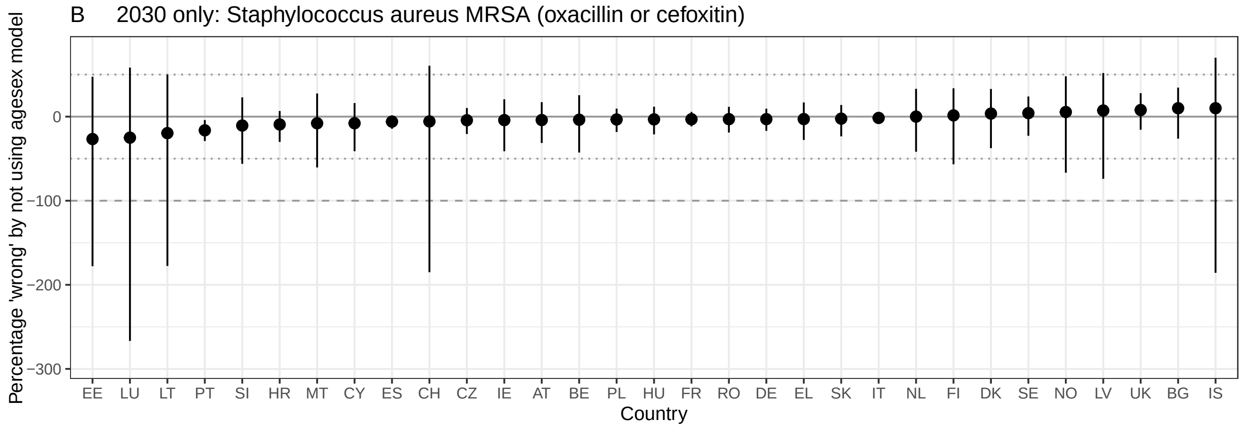

Figure A45: Difference between 'agesex' and 'base' model projections in 2030. A positive difference indicates that using the 'base' model underestimates resistant BSIs compared to the 'agesex' model. A) by age and sex. The line depicts the median and the ribbon the 95% quantiles. The dashed line at 0 indicates no difference. B) by country. The solid line indicates no difference, and the dotted / dashed lines represent a 50 and 100% difference respectively. The dot depicts the median and the ribbon the 95% quantiles. Staphylococcus aureus MRSA (oxacillin or cefoxitin).

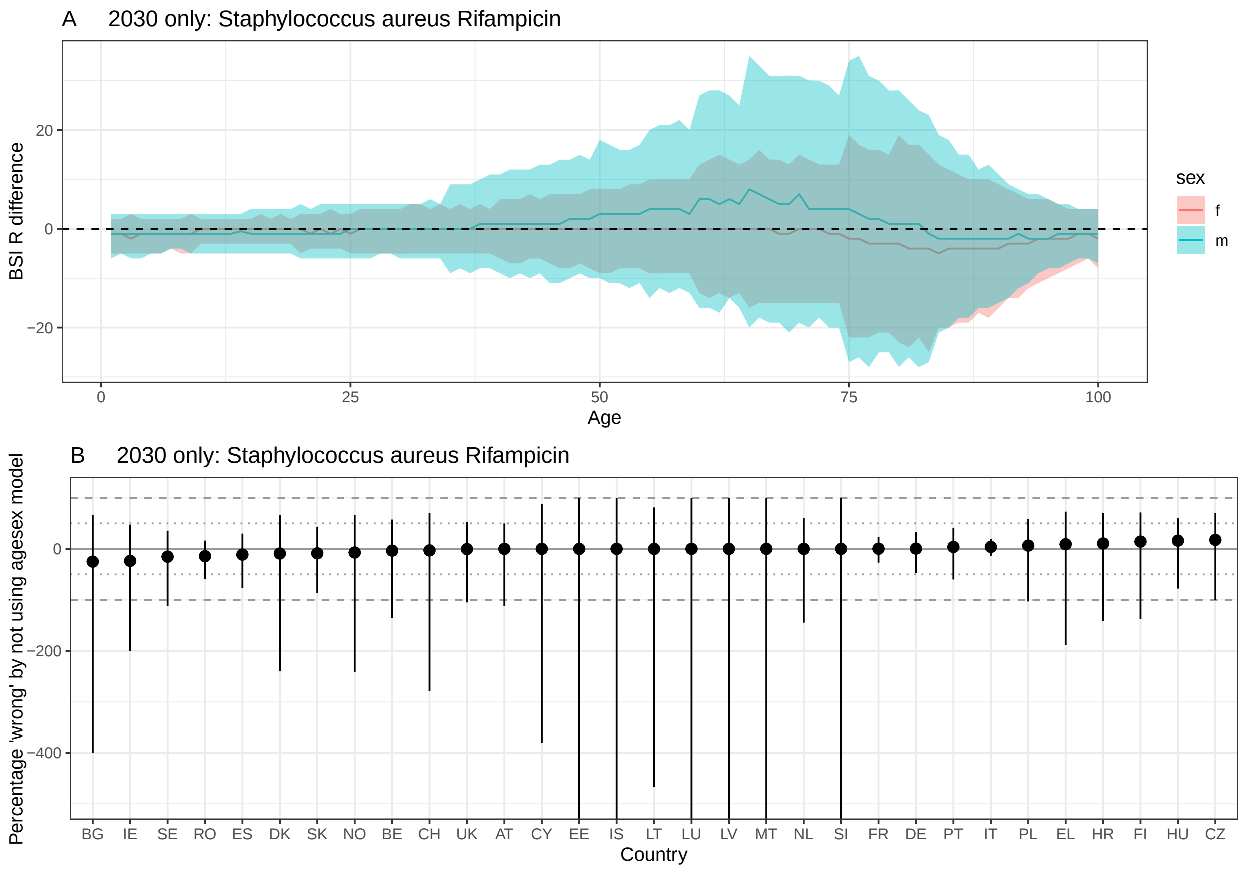

Figure A46: Difference between 'agesex' and 'base' model projections in 2030. A positive difference indicates that using the 'base' model underestimates resistant BSIs compared to the 'agesex' model. A) by age and sex. The line depicts the median and the ribbon the 95% quantiles. The dashed line at 0 indicates no difference. B) by country. The solid line indicates no difference, and the dotted / dashed lines represent a 50 and 100% difference respectively. The dot depicts the median and the ribbon the 95% quantiles. Staphylococcus aureus Rifampicin.

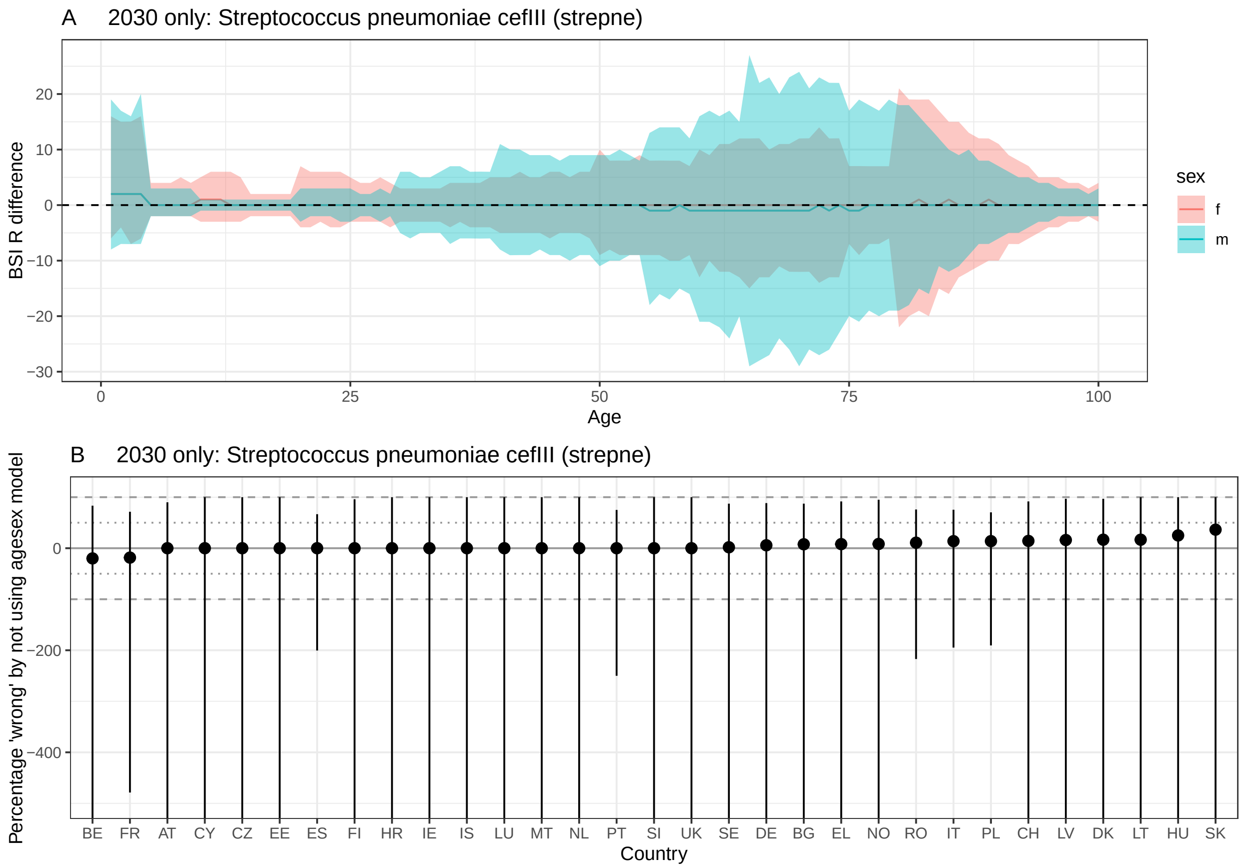

Figure A47: Difference between 'agesex' and 'base' model projections in 2030. A positive difference indicates that using the 'base' model underestimates resistant BSIs compared to the 'agesex' model. A) by age and sex. The line depicts the median and the ribbon the 95% quantiles. The dashed line at 0 indicates no difference. B) by country. The solid line indicates no difference, and the dotted / dashed lines represent a 50 and 100% difference respectively. The dot depicts the median and the ribbon the 95% quantiles. Streptococcus pneumoniae cefIII (strepne).

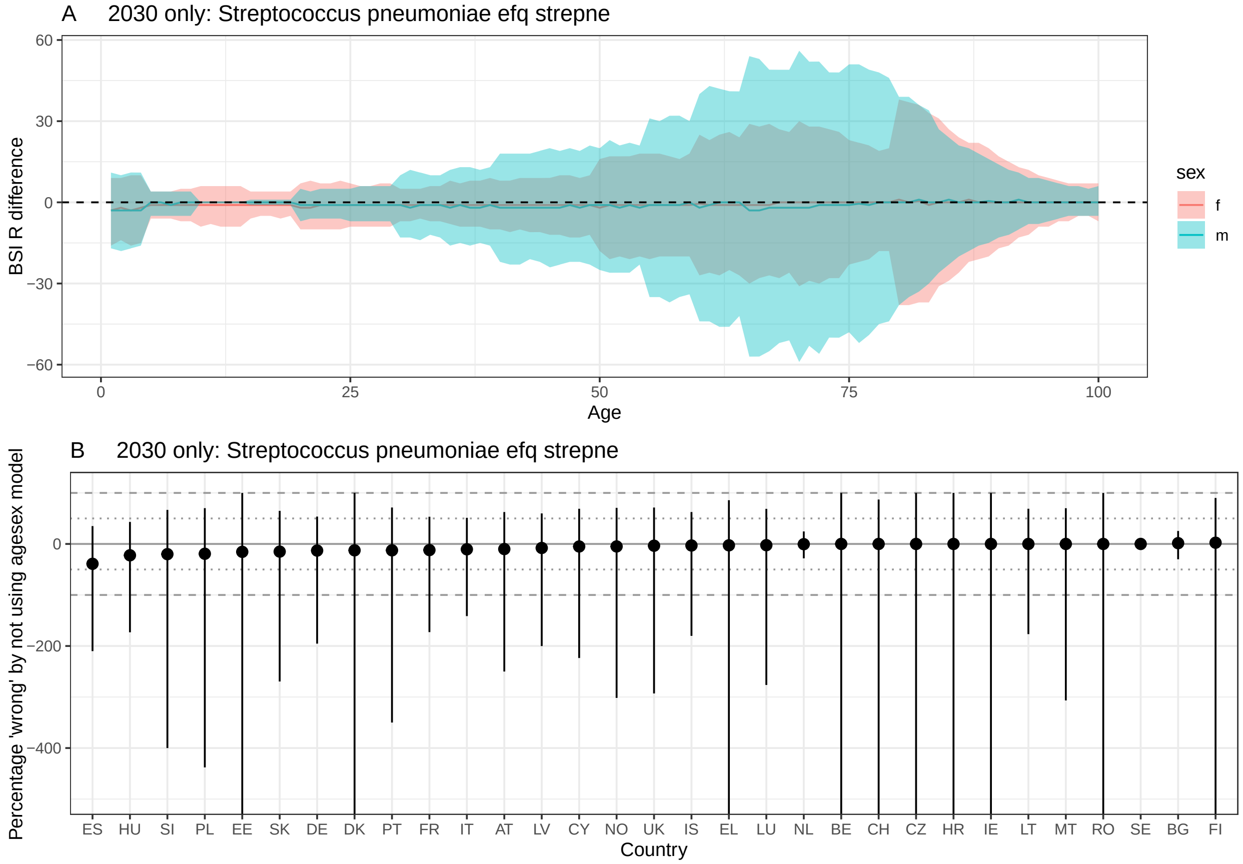

Figure A48: Difference between 'agesex' and 'base' model projections in 2030. A positive difference indicates that using the 'base' model underestimates resistant BSIs compared to the 'agesex' model. A) by age and sex. The line depicts the median and the ribbon the 95% quantiles. The dashed line at 0 indicates no difference. B) by country. The solid line indicates no difference, and the dotted / dashed lines represent a 50 and 100% difference respectively. The dot depicts the median and the ribbon the 95% quantiles. Streptococcus pneumoniae efq strepne.

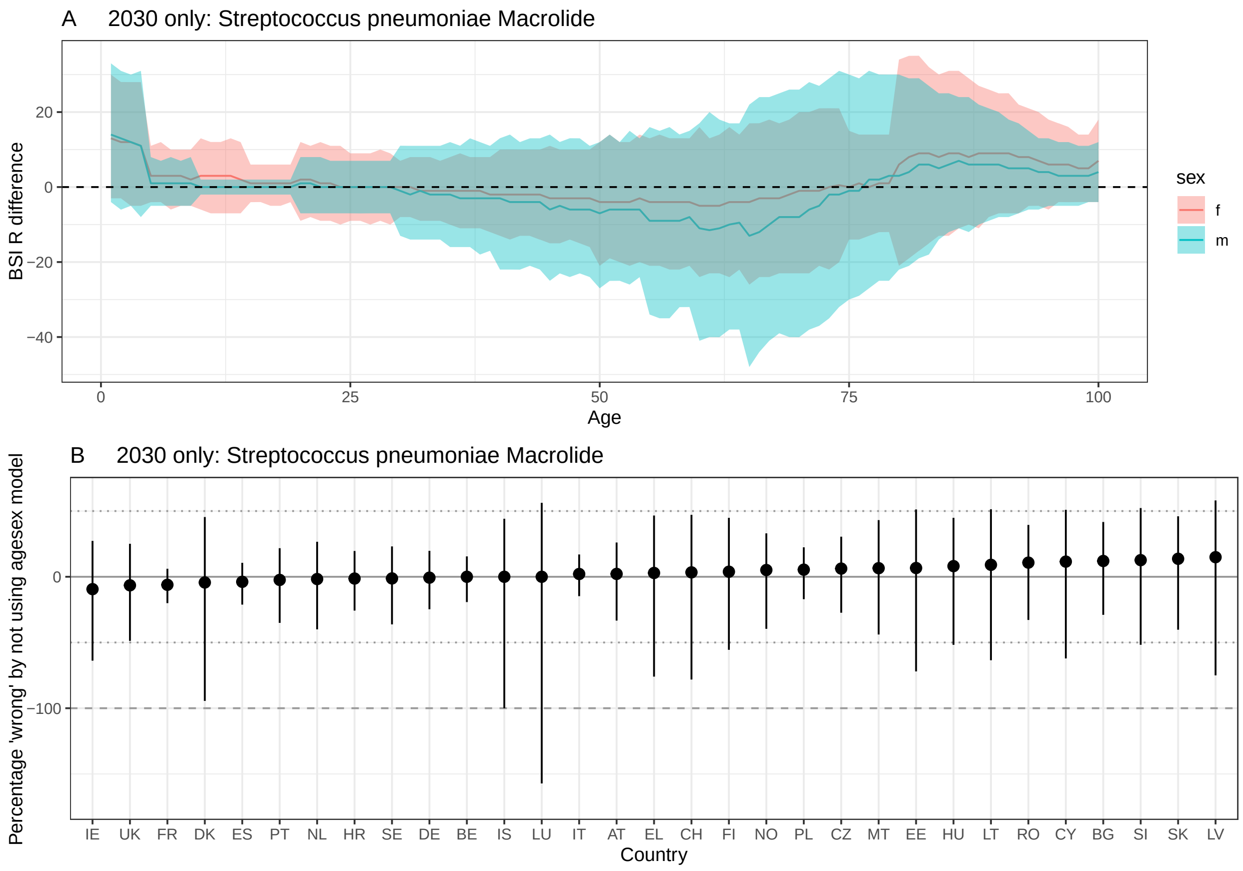

Figure A49: Difference between 'agesex' and 'base' model projections in 2030. A positive difference indicates that using the 'base' model underestimates resistant BSIs compared to the 'agesex' model. A) by age and sex. The line depicts the median and the ribbon the 95% quantiles. The dashed line at 0 indicates no difference. B) by country. The solid line indicates no difference, and the dotted / dashed lines represent a 50 and 100% difference respectively. The dot depicts the median and the ribbon the 95% quantiles. Streptococcus pneumoniae Macrolide.

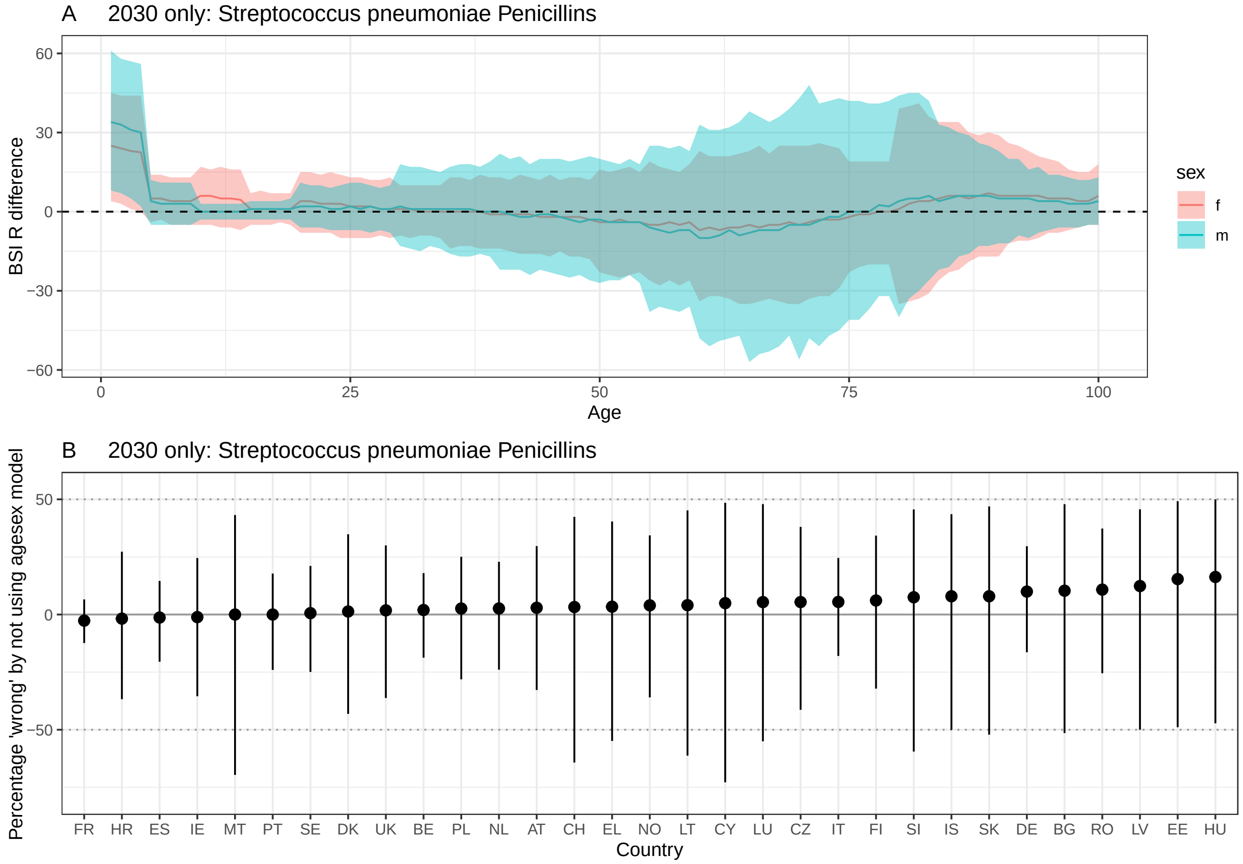

Figure A50: Difference between 'agesex' and 'base' model projections in 2030. A positive difference indicates that using the 'base' model underestimates resistant BSIs compared to the 'agesex' model. A) by age and sex. The line depicts the median and the ribbon the 95% quantiles. The dashed line at 0 indicates no difference. B) by country. The solid line indicates no difference, and the dotted / dashed lines represent a 50 and 100% difference respectively. The dot depicts the median and the ribbon the 95% quantiles. Streptococcus pneumoniae Penicillins.
