# Supplementary material for: Combining demographic shifts with age-based resistance prevalence to estimate future antimicrobial resistance burden in Europe and implications for targets: A modelling study
Source: PLoS Med. 2025 Nov 4;22(11):e1004579. doi: 10.1371/journal.pmed.1004579 (PMC12585039; doi:10.1371/journal.pmed.1004579)
Supplement: S3 Appendix — Figs A1–A38: Resistant BSI projections relative to 2019 cases, for each of the intervention scenarios (colour) as in Fig 5. The dashed line is at 0.9 (indicating a 10% relative reduction), and the dotted line at 2030, indicating the UN targets. The line depicts the median and the ribbon the 95% quantiles. The interventions reduce the annual rate of change of BSI incidence by minus 1, 5, or 20 per 100,000 in all ages or in only those older than 65 (minus5over65). A1 – Acinetobacter species Amikacin, A2 – Acinetobacter species Aminoglycosides, A3 – Acinetobacter species Carbapenems, A4 – Acinetobacter species Fluoroquinolones, A5 – Enterococcus faecalis Aminopenicillins, A6 – Enterococcus faecalis High-level aminoglycoside, A7 – Enterococcus faecalis Vancomycin, A8 – Enterococcus faecium Aminopenicillins, A9 – Enterococcus faecium High-level aminoglycoside, A10 – Enterococcus faecium Vancomycin, A11 – Escherichia coli Amikacin, A12 – Escherichia coli Aminoglycosides, A13 – Escherichia coli Aminopenicillins, A14 – Escherichia coli Carbapenems, A15 – Escherichia coli Third-generation cephalosporins, A16 – Escherichia coli Ertapenem, A17 – Escherichia coli Fluoroquinolones, A18 – Escherichia coli Piperacillin–tazobactam, A19 – Klebsiella pneumoniae Amikacin, A20 – Klebsiella pneumoniae Aminoglycosides, A21 – Klebsiella pneumoniae Carbapenems, A22 – Klebsiella pneumoniae Third-generation cephalosporins, A23 – Klebsiella pneumoniae Ertapenem, A24 – Klebsiella pneumoniae Fluoroquinolones, A25 – Klebsiella pneumoniae Piperacillin–tazobactam, A26 – Pseudomonas aeruginosa Amikacin, A27 – Pseudomonas aeruginosa Aminoglycoside, A28 – Pseudomonas aeruginosa Carbapenem, A29 – Pseudomonas aeruginosa Ceftazidime, A30 – Pseudomonas aeruginosa Fluoroquinolone, A31 – Pseudomonas aeruginosa Piperacillin–tazobactam, A32 – Staphylococcus aureus Fluoroquinolone, A33 – Staphylococcus aureus MRSA (oxacillin or cefoxitin), A34 – Staphylococcus aureus Rifampicin, A35 – Streptococcus pneumoniae [file pmed.1004579.s003.pdf]

S3 Appendix

Contents

1 - Intervention impacts **1**

1 - Intervention impacts

The impact of the different interventions on the resistant BSI projections is shown in the following Figures A1 - A38 for each bacteria-antibiotic combination.

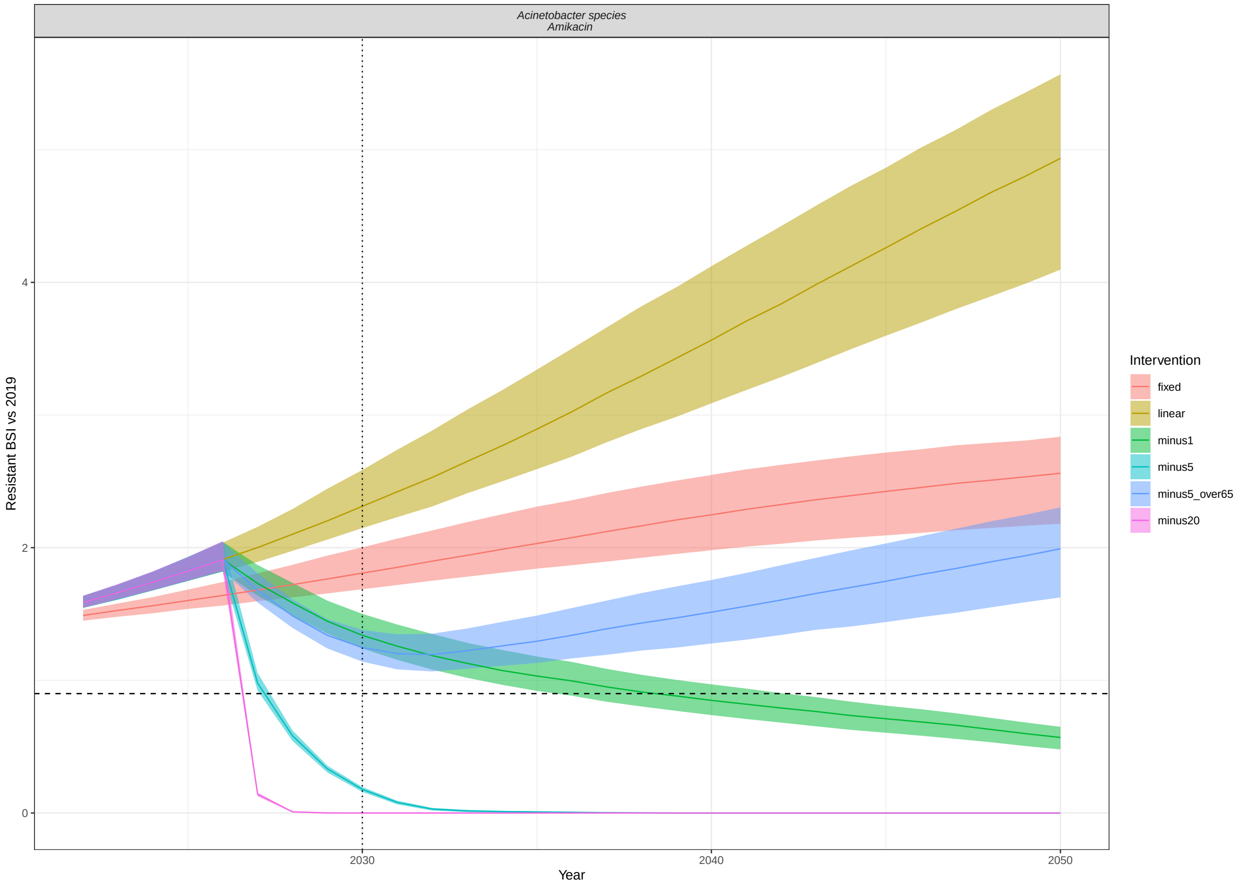

Figure A1: Resistant BSI projections relative to 2019 cases, for each of the intervention scenarios (colour) as in Figure 5. The dashed line is at 0.9 (indicating a 10% relative reduction), and the dotted line at 2030, indicating the UN targets. The line depicts the median and the ribbon the 95% quantiles. The interventions reduce the annual rate of change of BSI incidence by minus 1, 5 or 20 per 100,000 in all ages or in only those older than 65 (minus5over65).Acinetobacter species Amikacin .

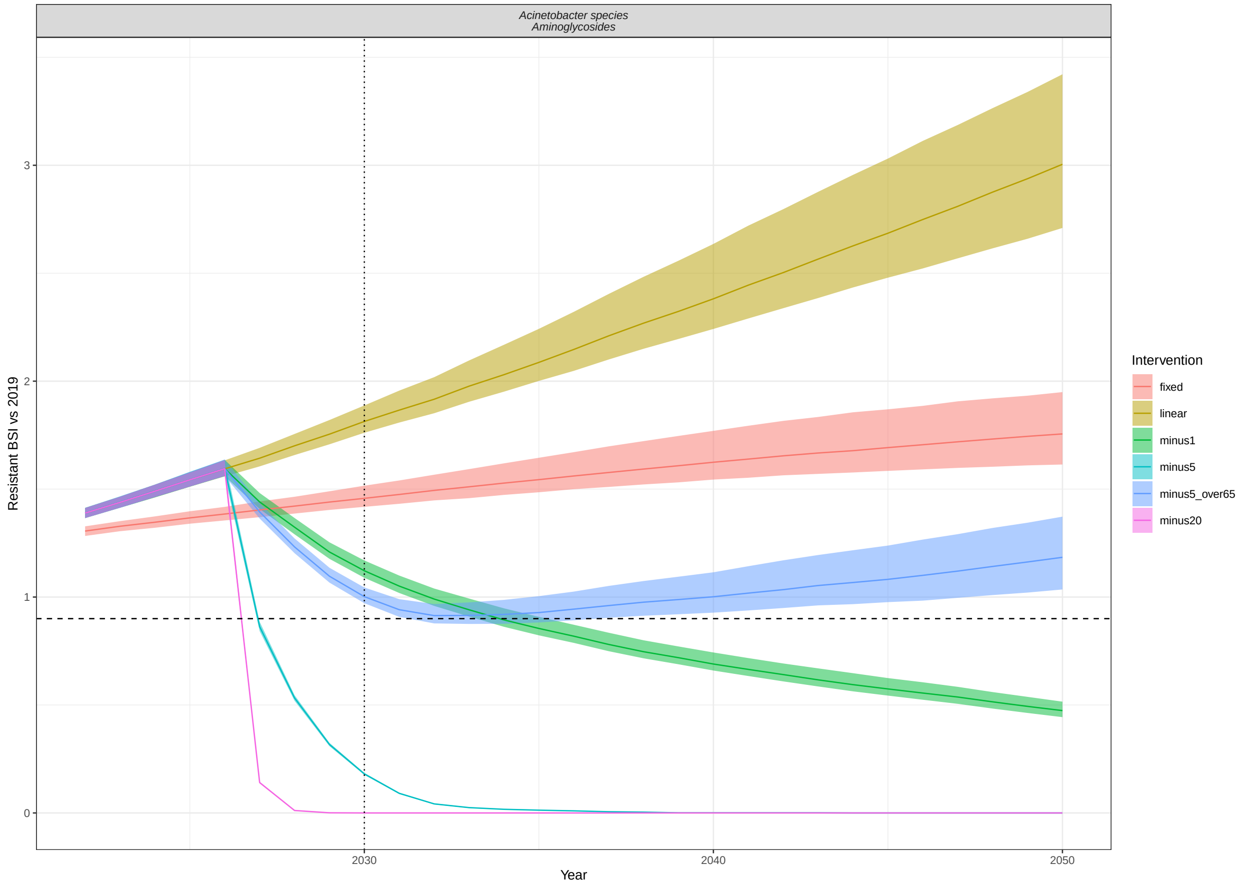

Figure A2: Resistant BSI projections relative to 2019 cases, for each of the intervention scenarios (colour) as in Figure 5. The dashed line is at 0.9 (indicating a 10% relative reduction), and the dotted line at 2030, indicating the UN targets. The line depicts the median and the ribbon the 95% quantiles. The interventions reduce the annual rate of change of BSI incidence by minus 1/5/20 per 100,000 in all ages or in only those aged 65+ (minus5over65). Acinetobacter species Aminoglycosides.

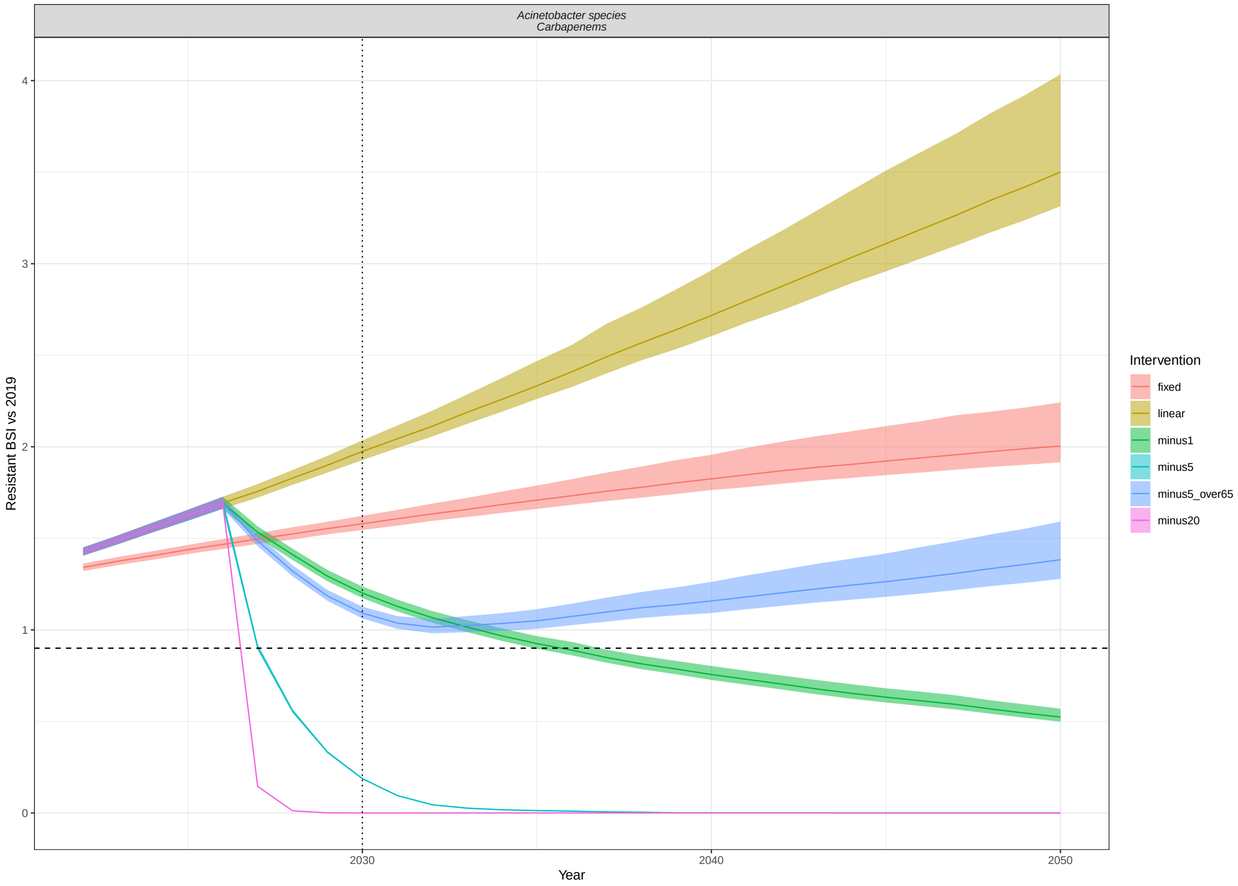

Figure A3: Resistant BSI projections relative to 2019 cases, for each of the intervention scenarios (colour) as in Figure 5. The dashed line is at 0.9 (indicating a 10% relative reduction), and the dotted line at 2030, indicating the UN targets. The line depicts the median and the ribbon the 95% quantiles. The interventions reduce the annual rate of change of BSI incidence by minus 1/5/20 per 100,000 in all ages or in only those aged 65+ (minus5over65). Acinetobacter species Carbapenems.

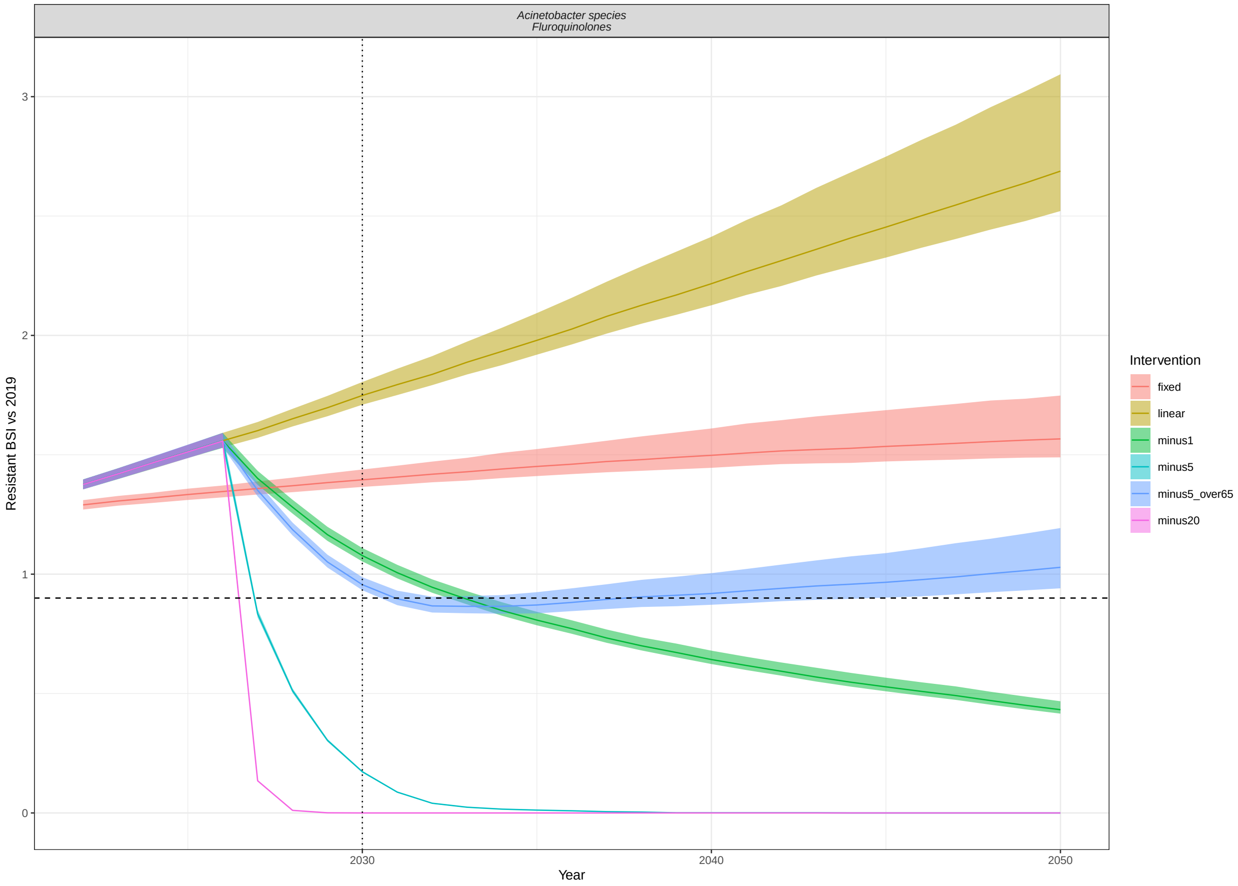

Figure A4: Resistant BSI projections relative to 2019 cases, for each of the intervention scenarios (colour) as in Figure 5. The dashed line is at 0.9 (indicating a 10% relative reduction), and the dotted line at 2030, indicating the UN targets. The line depicts the median and the ribbon the 95% quantiles. The interventions reduce the annual rate of change of BSI incidence by minus 1/5/20 per 100,000 in all ages or in only those aged 65+ (minus5over65). Acinetobacter species Fluroquinolones.

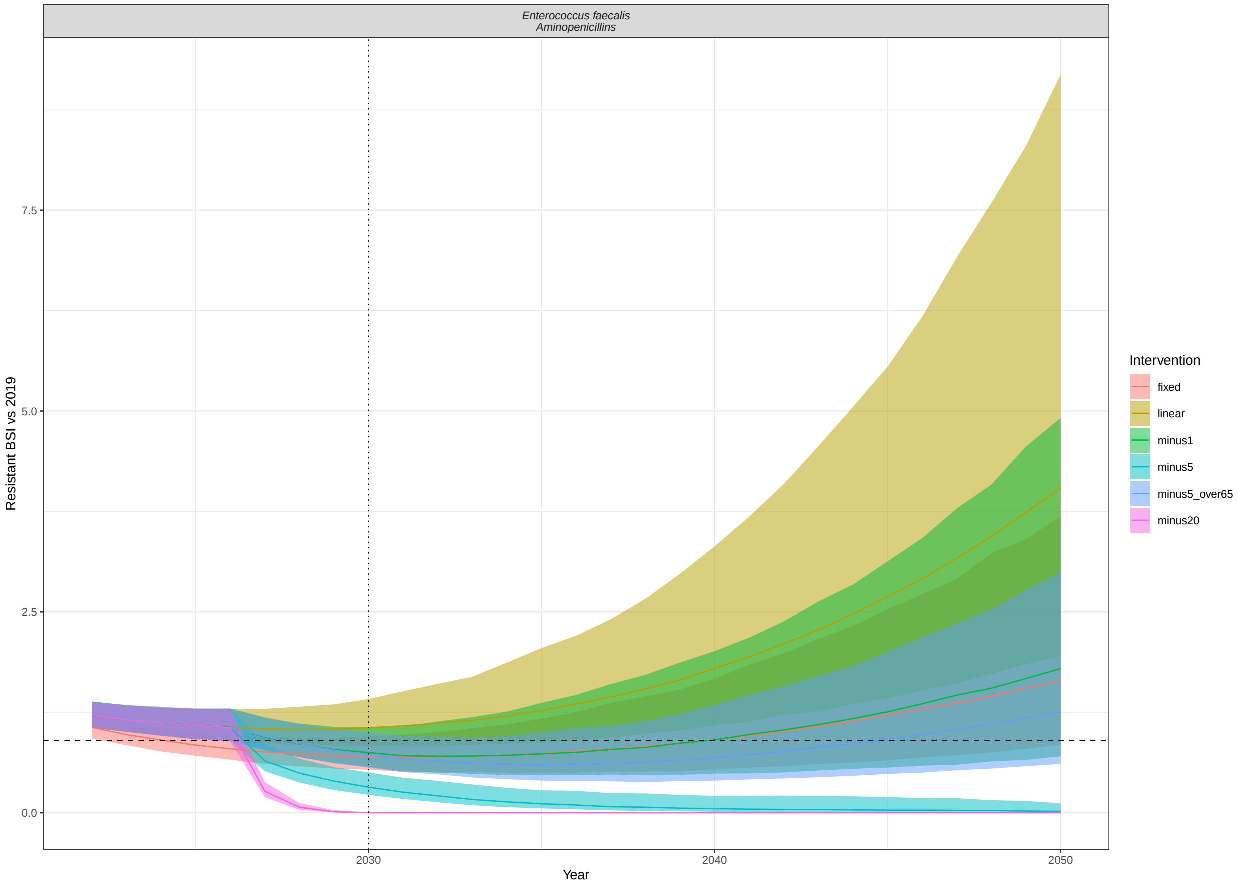

Figure A5: Resistant BSI projections relative to 2019 cases, for each of the intervention scenarios (colour) as in Figure 5. The dashed line is at 0.9 (indicating a 10% relative reduction), and the dotted line at 2030, indicating the UN targets. The line depicts the median and the ribbon the 95% quantiles. The interventions reduce the annual rate of change of BSI incidence by minus 1/5/20 per 100,000 in all ages or in only those aged 65+ (minus5over65). Enterococcus faecalis Aminopenicillins.

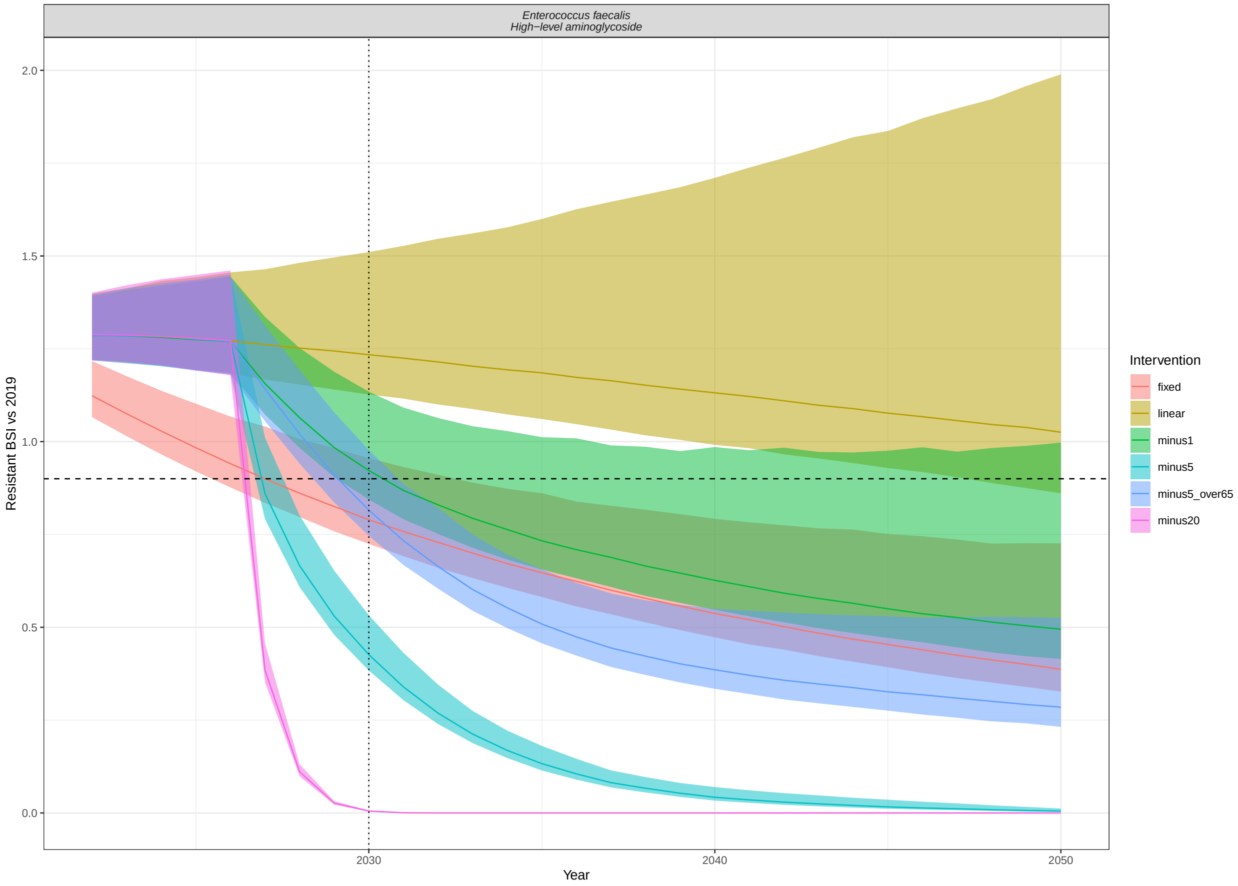

Figure A6: Resistant BSI projections relative to 2019 cases, for each of the intervention scenarios (colour) as in Figure 5. The dashed line is at 0.9 (indicating a 10% relative reduction), and the dotted line at 2030, indicating the UN targets. The line depicts the median and the ribbon the 95% quantiles. The interventions reduce the annual rate of change of BSI incidence by minus 1/5/20 per 100,000 in all ages or in only those aged 65+ (minus5over65). Enterococcus faecalis High-level aminoglycoside .

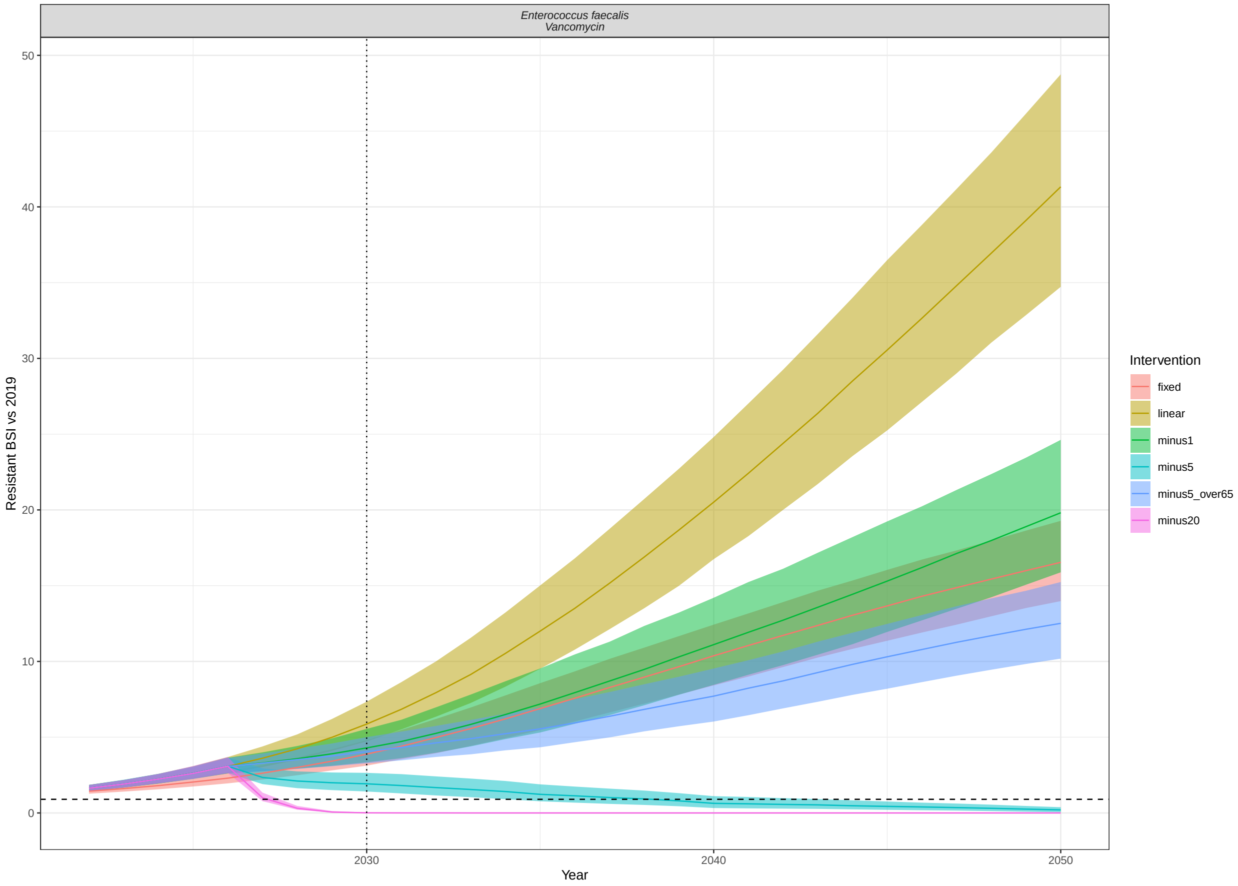

Figure A7: Resistant BSI projections relative to 2019 cases, for each of the intervention scenarios (colour) as in Figure 5. The dashed line is at 0.9 (indicating a 10% relative reduction), and the dotted line at 2030, indicating the UN targets. The line depicts the median and the ribbon the 95% quantiles. The interventions reduce the annual rate of change of BSI incidence by minus 1/5/20 per 100,000 in all ages or in only those aged 65+ (minus5over65). Enterococcus faecalis Vancomycin.

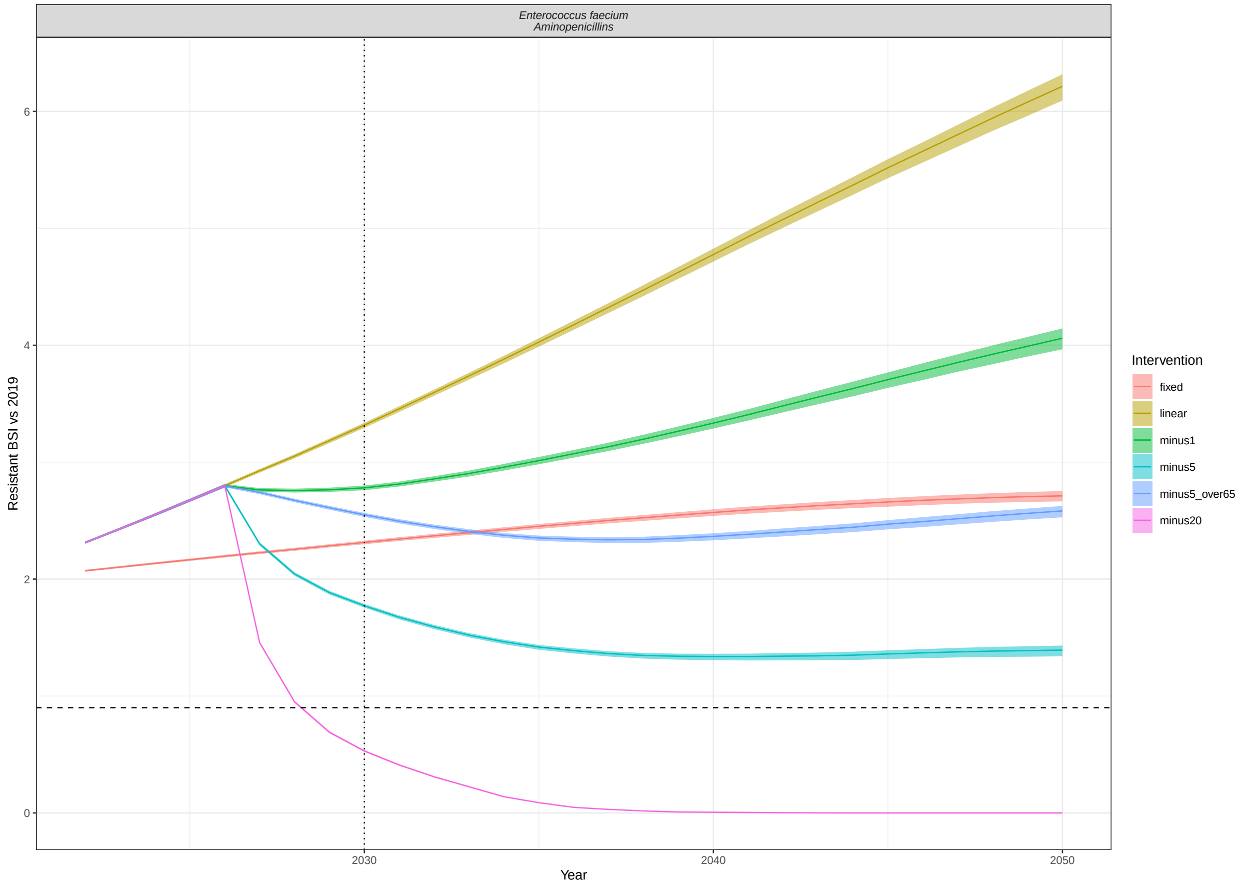

Figure A8: Resistant BSI projections relative to 2019 cases, for each of the intervention scenarios (colour) as in Figure 5. The dashed line is at 0.9 (indicating a 10% relative reduction), and the dotted line at 2030, indicating the UN targets. The line depicts the median and the ribbon the 95% quantiles. The interventions reduce the annual rate of change of BSI incidence by minus 1/5/20 per 100,000 in all ages or in only those aged 65+ (minus5over65). Enterococcus faecium Aminopenicillins.

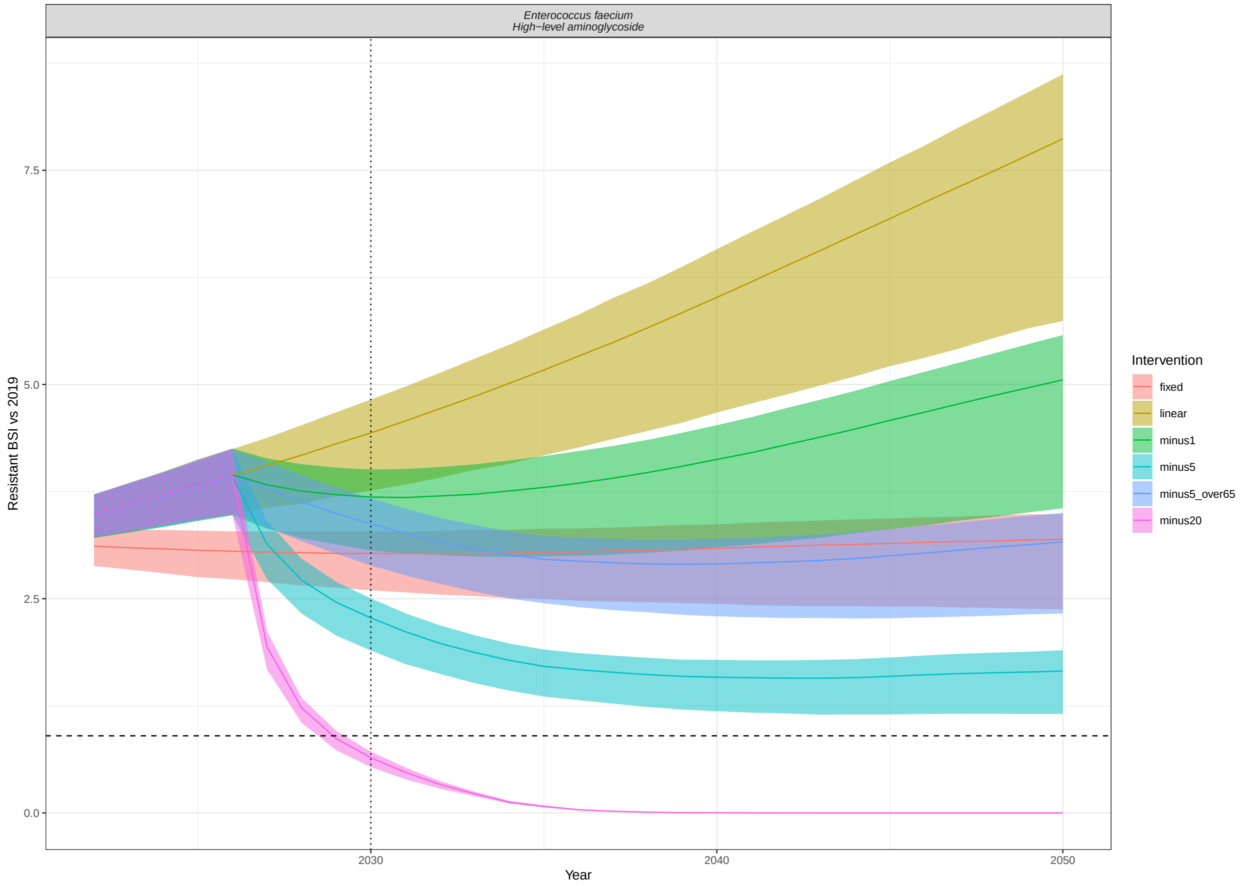

Figure A9: Resistant BSI projections relative to 2019 cases, for each of the intervention scenarios (colour) as in Figure 5. The dashed line is at 0.9 (indicating a 10% relative reduction), and the dotted line at 2030, indicating the UN targets. The line depicts the median and the ribbon the 95% quantiles. The interventions reduce the annual rate of change of BSI incidence by minus 1/5/20 per 100,000 in all ages or in only those aged 65+ (minus5over65). Enterococcus faecium High-level aminoglycoside .

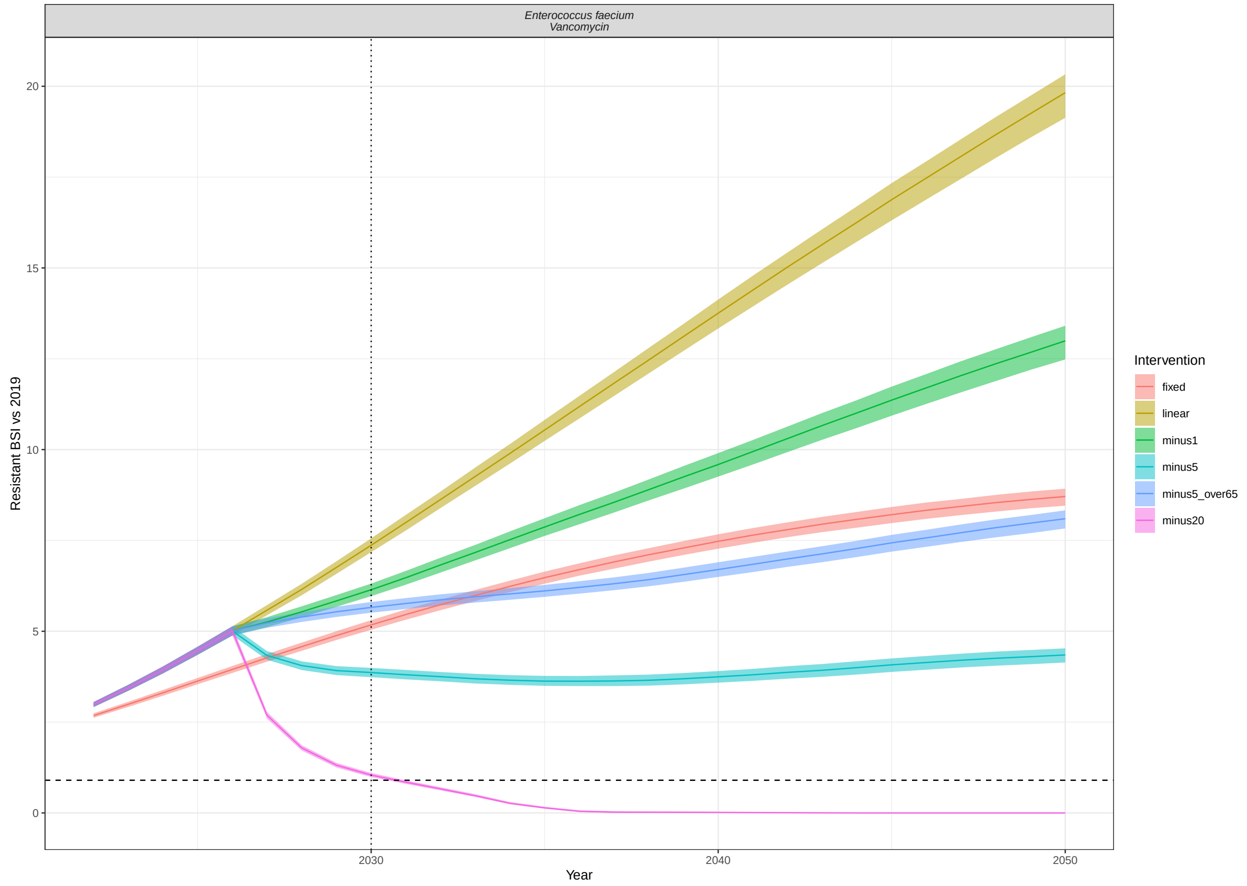

Figure A10: Resistant BSI projections relative to 2019 cases, for each of the intervention scenarios (colour) as in Figure 5. The dashed line is at 0.9 (indicating a 10% relative reduction), and the dotted line at 2030, indicating the UN targets. The line depicts the median and the ribbon the 95% quantiles. The interventions reduce the annual rate of change of BSI incidence by minus 1/5/20 per 100,000 in all ages or in only those aged 65+ (minus5over65). Enterococcus faecium Vancomycin.

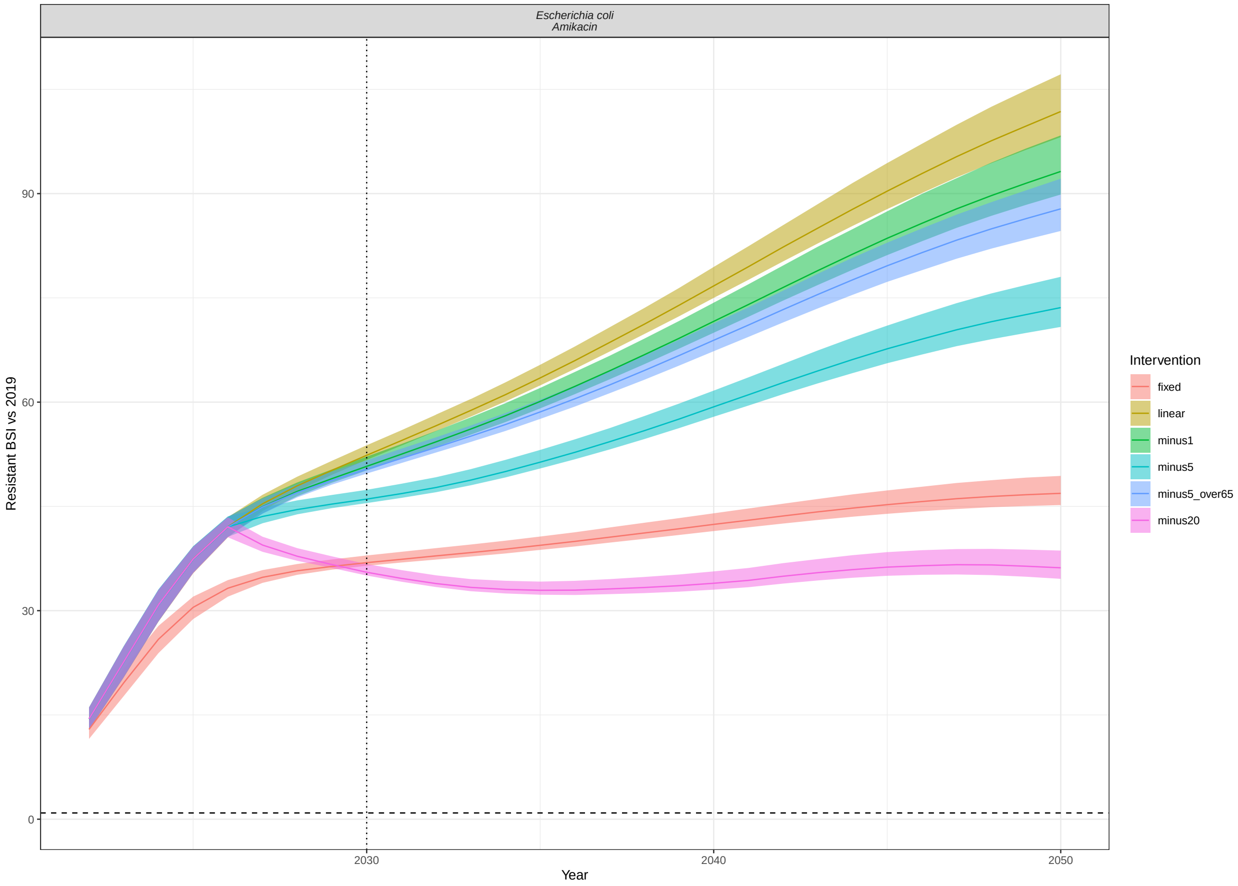

Figure A11: Resistant BSI projections relative to 2019 cases, for each of the intervention scenarios (colour) as in Figure 5. The dashed line is at 0.9 (indicating a 10% relative reduction), and the dotted line at 2030, indicating the UN targets. The line depicts the median and the ribbon the 95% quantiles. The interventions reduce the annual rate of change of BSI incidence by minus 1/5/20 per 100,000 in all ages or in only those aged 65+ (minus5over65). Escherichia coli Amikacin.

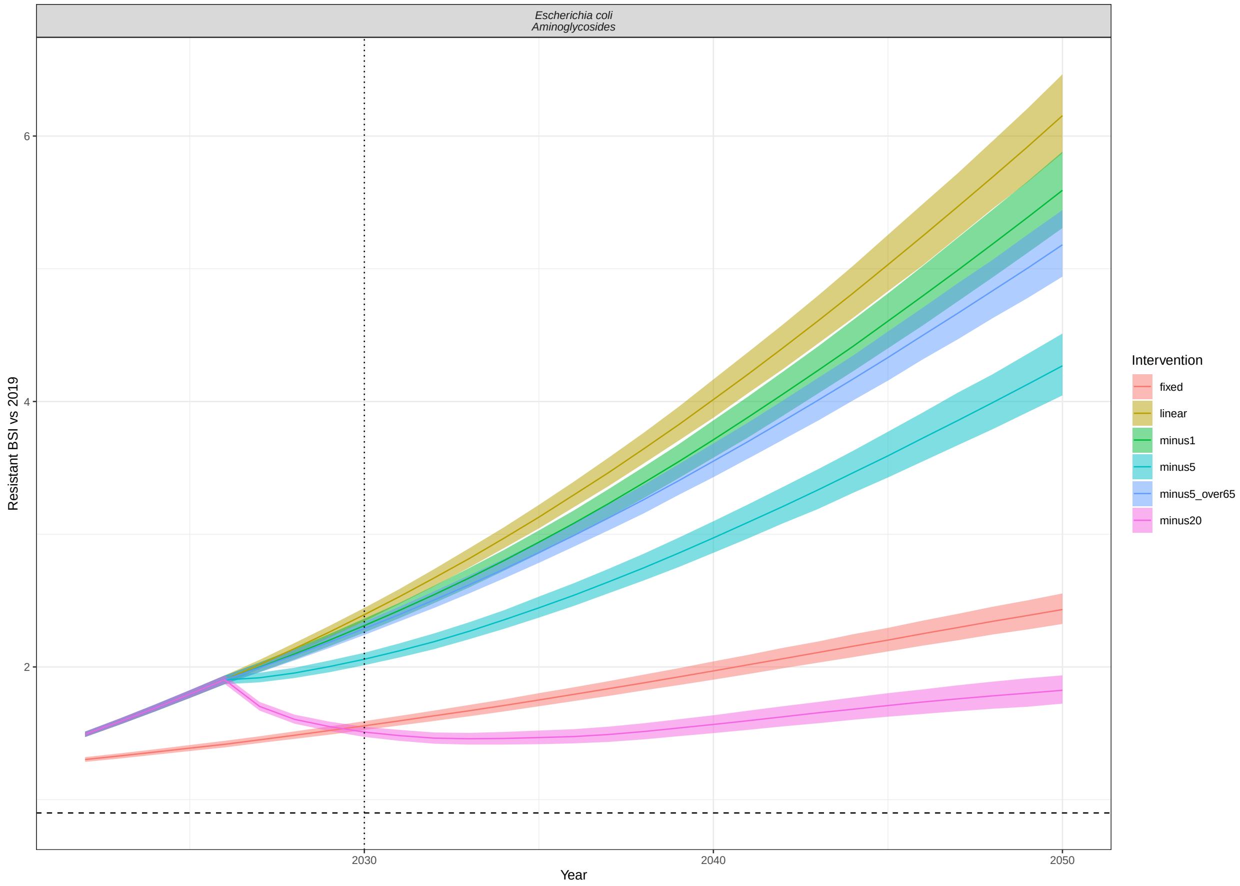

Figure A12: Resistant BSI projections relative to 2019 cases, for each of the intervention scenarios (colour) as in Figure 5. The dashed line is at 0.9 (indicating a 10% relative reduction), and the dotted line at 2030, indicating the UN targets. The line depicts the median and the ribbon the 95% quantiles. The interventions reduce the annual rate of change of BSI incidence by minus 1/5/20 per 100,000 in all ages or in only those aged 65+ (minus5over65). Escherichia coli Aminoglycosides.

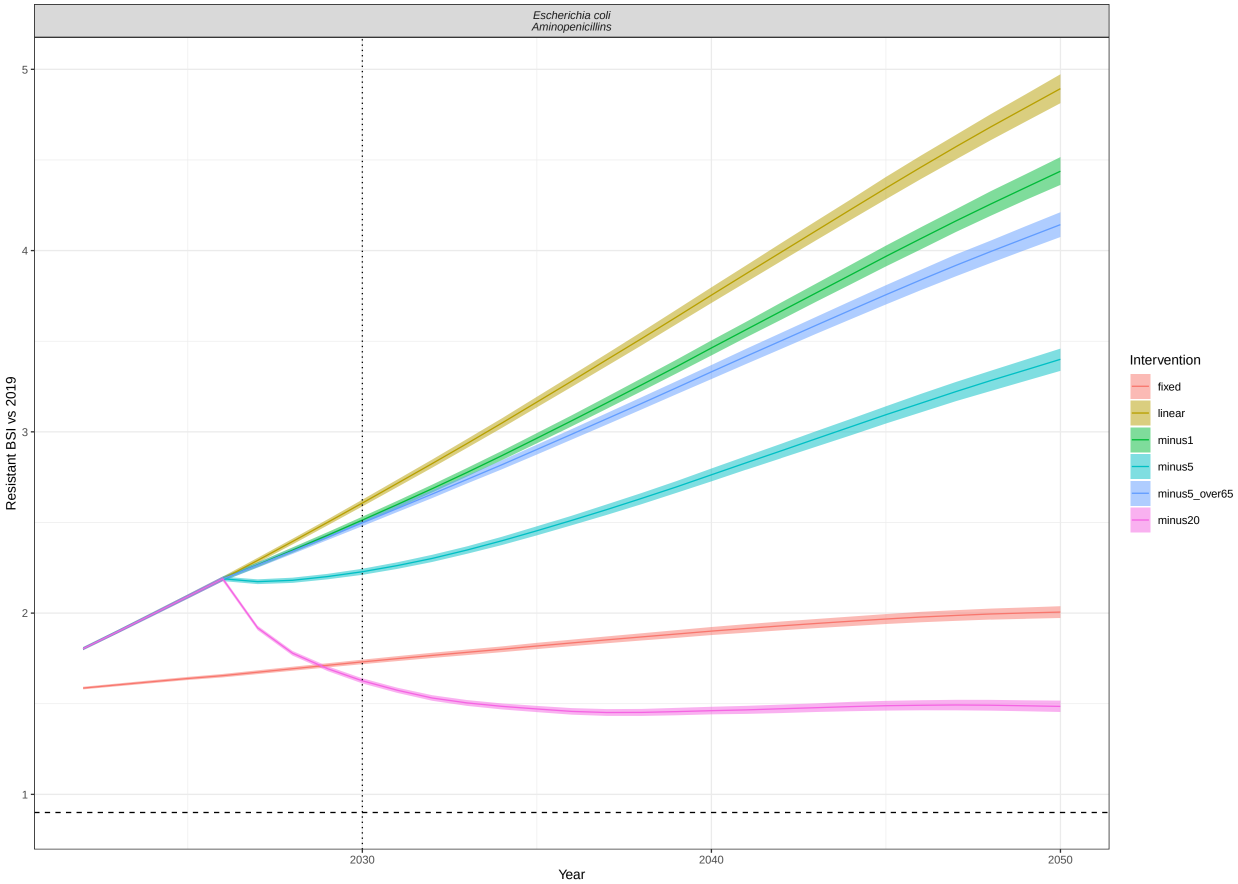

Figure A13: Resistant BSI projections relative to 2019 cases, for each of the intervention scenarios (colour) as in Figure 5. The dashed line is at 0.9 (indicating a 10% relative reduction), and the dotted line at 2030, indicating the UN targets. The line depicts the median and the ribbon the 95% quantiles. The interventions reduce the annual rate of change of BSI incidence by minus 1/5/20 per 100,000 in all ages or in only those aged 65+ (minus5over65). Escherichia coli Aminopenicillins.

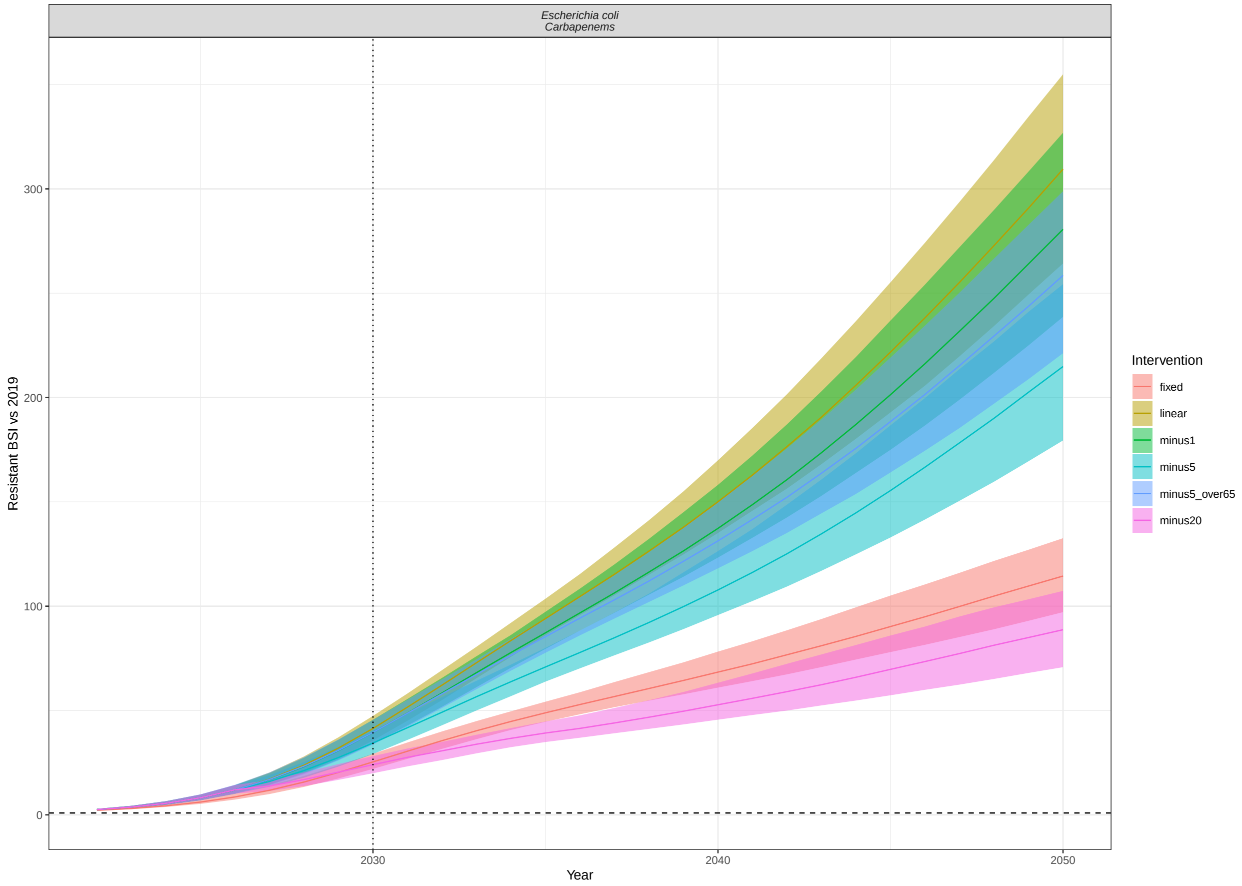

Figure A14: Resistant BSI projections relative to 2019 cases, for each of the intervention scenarios (colour) as in Figure 5. The dashed line is at 0.9 (indicating a 10% relative reduction), and the dotted line at 2030, indicating the UN targets. The line depicts the median and the ribbon the 95% quantiles. The interventions reduce the annual rate of change of BSI incidence by minus 1/5/20 per 100,000 in all ages or in only those aged 65+ (minus5over65). Escherichia coli Carbapenems.

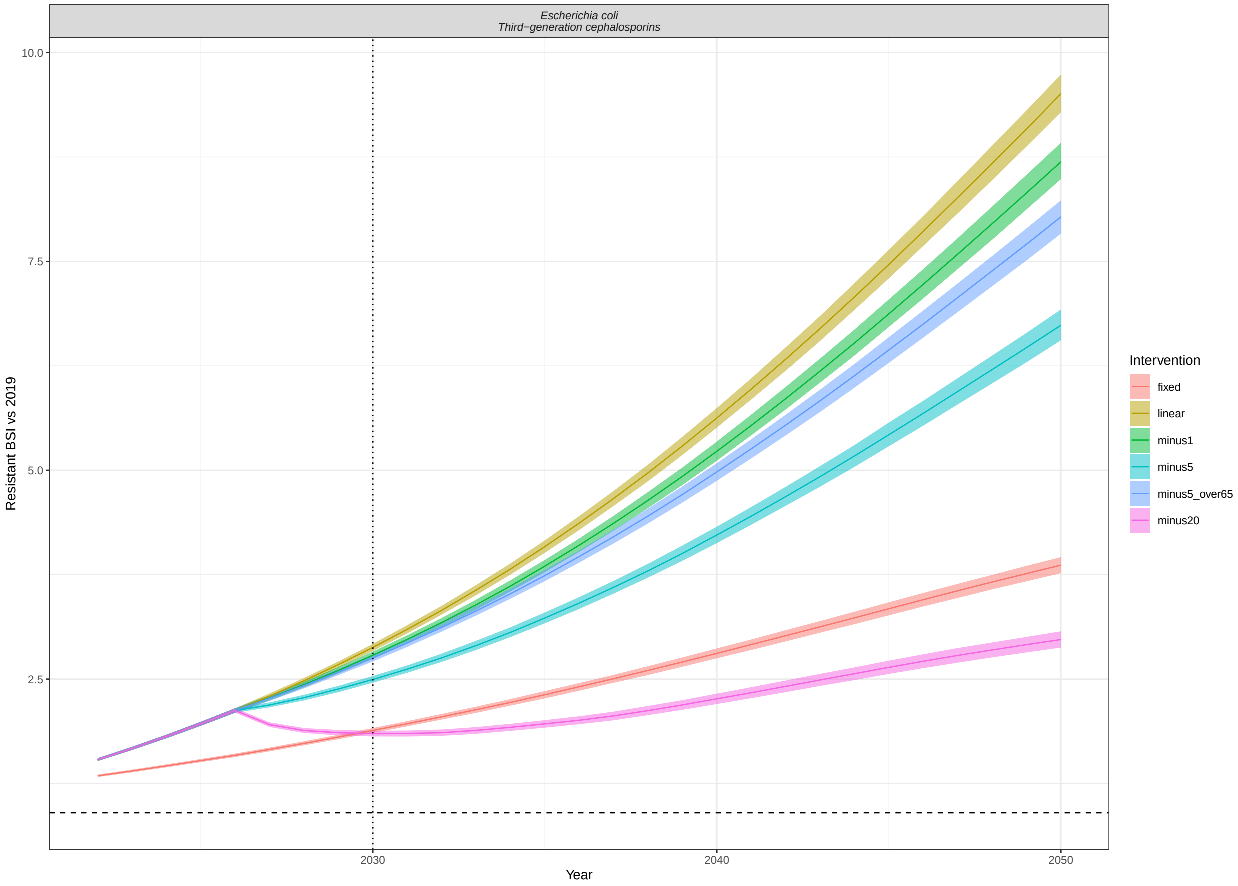

Figure A15: Resistant BSI projections relative to 2019 cases, for each of the intervention scenarios (colour) as in Figure 5. The dashed line is at 0.9 (indicating a 10% relative reduction), and the dotted line at 2030, indicating the UN targets. The line depicts the median and the ribbon the 95% quantiles. The interventions reduce the annual rate of change of BSI incidence by minus 1/5/20 per 100,000 in all ages or in only those aged 65+ (minus5over65). Escherichia coli Third-generation cephalosporins.

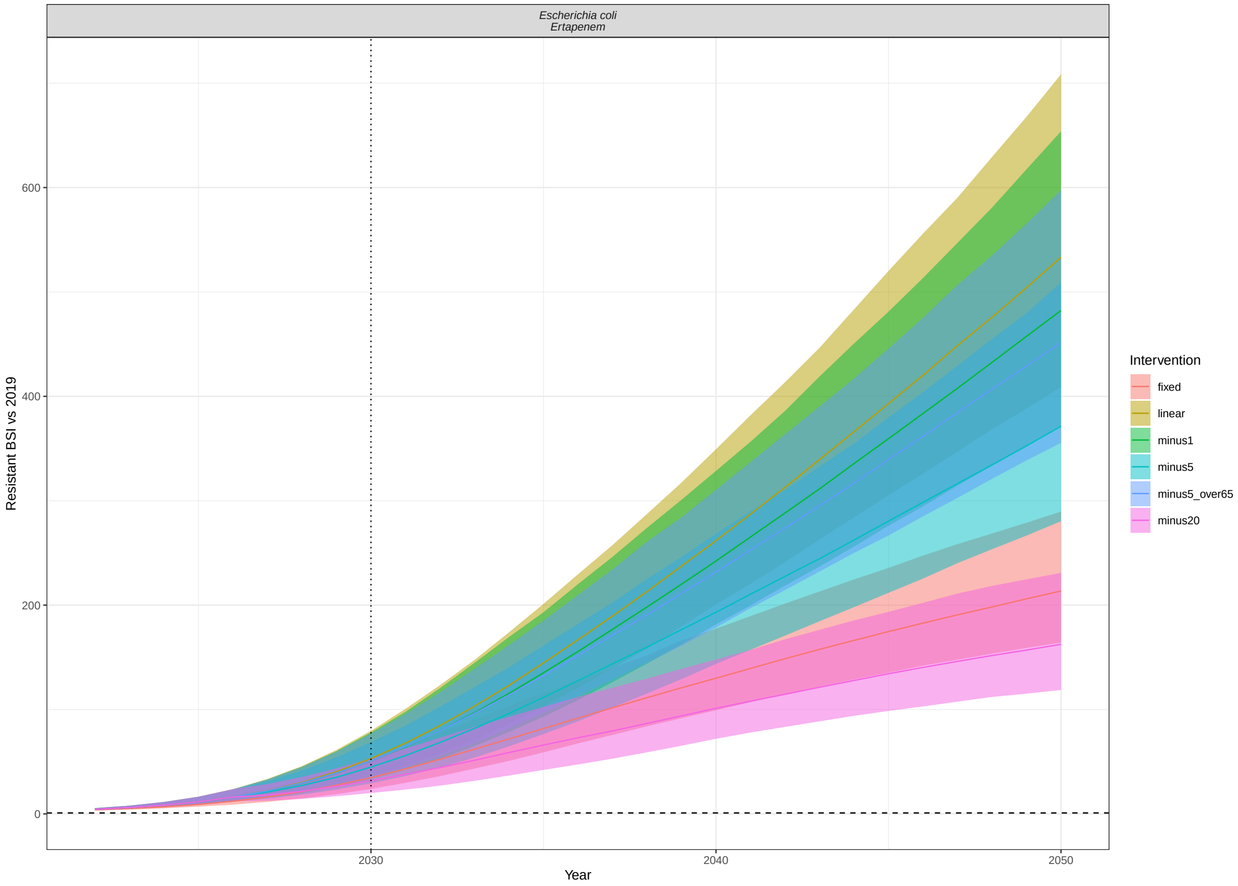

Figure A16: Resistant BSI projections relative to 2019 cases, for each of the intervention scenarios (colour) as in Figure 5. The dashed line is at 0.9 (indicating a 10% relative reduction), and the dotted line at 2030, indicating the UN targets. The line depicts the median and the ribbon the 95% quantiles. The interventions reduce the annual rate of change of BSI incidence by minus 1/5/20 per 100,000 in all ages or in only those aged 65+ (minus5over65). Escherichia coli Ertapenem.

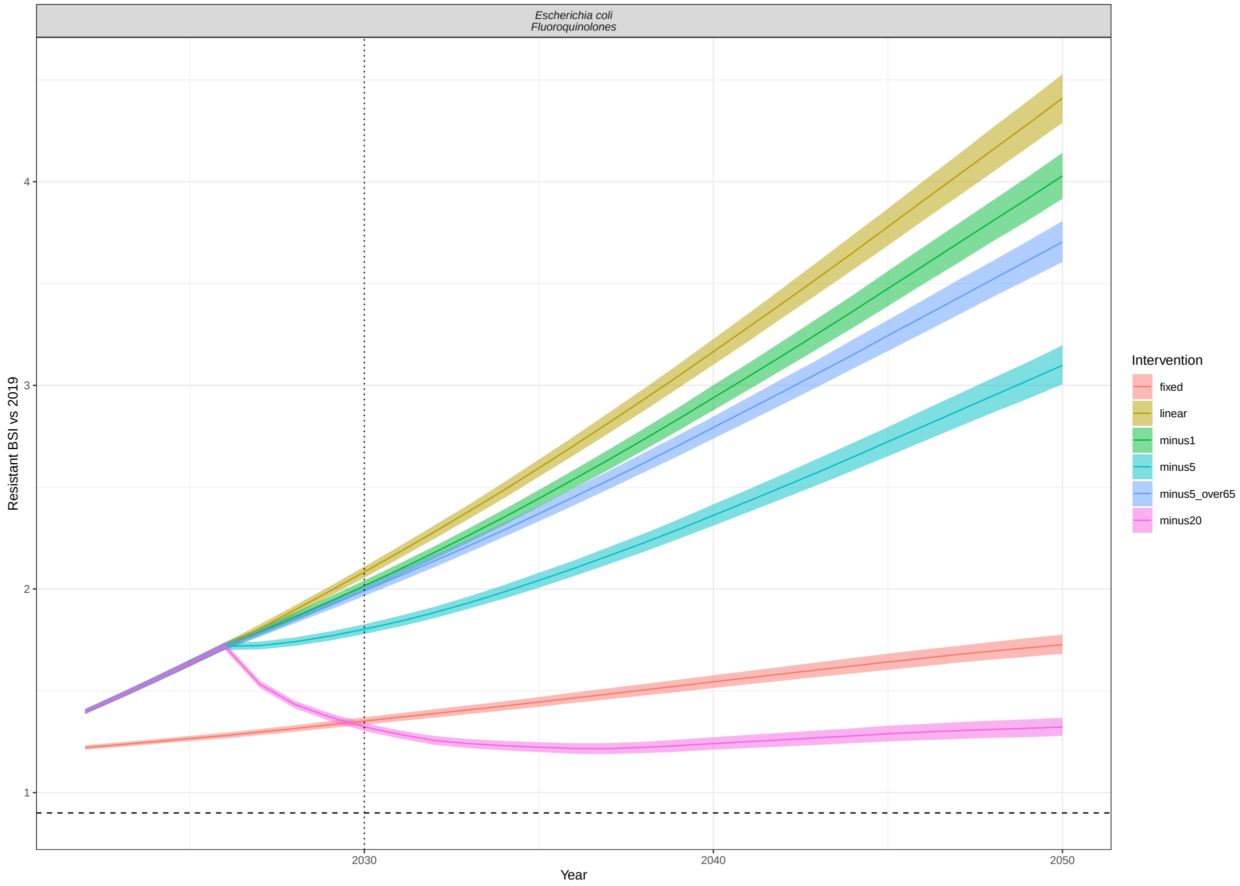

Figure A17: Resistant BSI projections relative to 2019 cases, for each of the intervention scenarios (colour) as in Figure 5. The dashed line is at 0.9 (indicating a 10% relative reduction), and the dotted line at 2030, indicating the UN targets. The line depicts the median and the ribbon the 95% quantiles. The interventions reduce the annual rate of change of BSI incidence by minus 1/5/20 per 100,000 in all ages or in only those aged 65+ (minus5over65). Escherichia coli Fluoroquinolones.

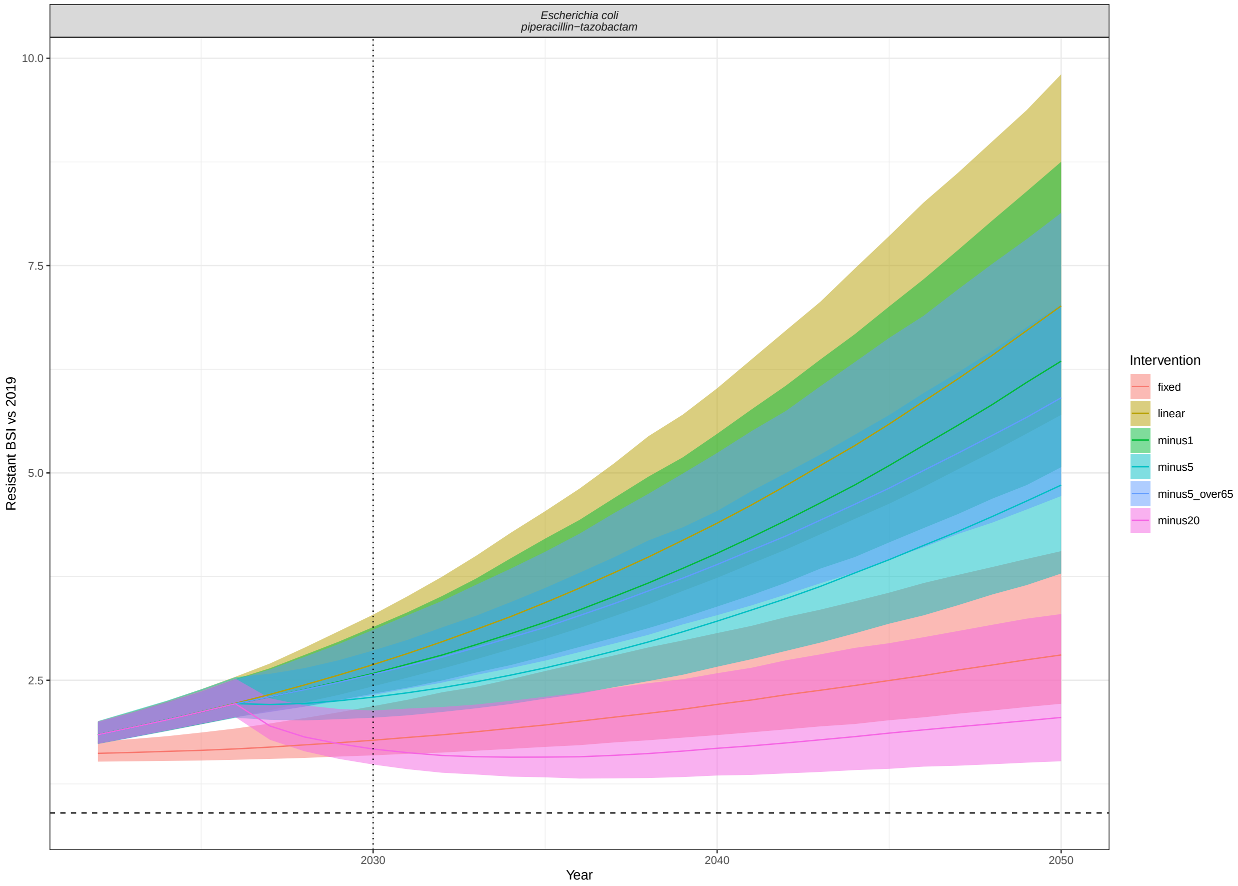

Figure A18: Resistant BSI projections relative to 2019 cases, for each of the intervention scenarios (colour) as in Figure 5. The dashed line is at 0.9 (indicating a 10% relative reduction), and the dotted line at 2030, indicating the UN targets. The line depicts the median and the ribbon the 95% quantiles. The interventions reduce the annual rate of change of BSI incidence by minus 1/5/20 per 100,000 in all ages or in only those aged 65+ (minus5over65). Escherichia coli piperacillin-tazobactam.

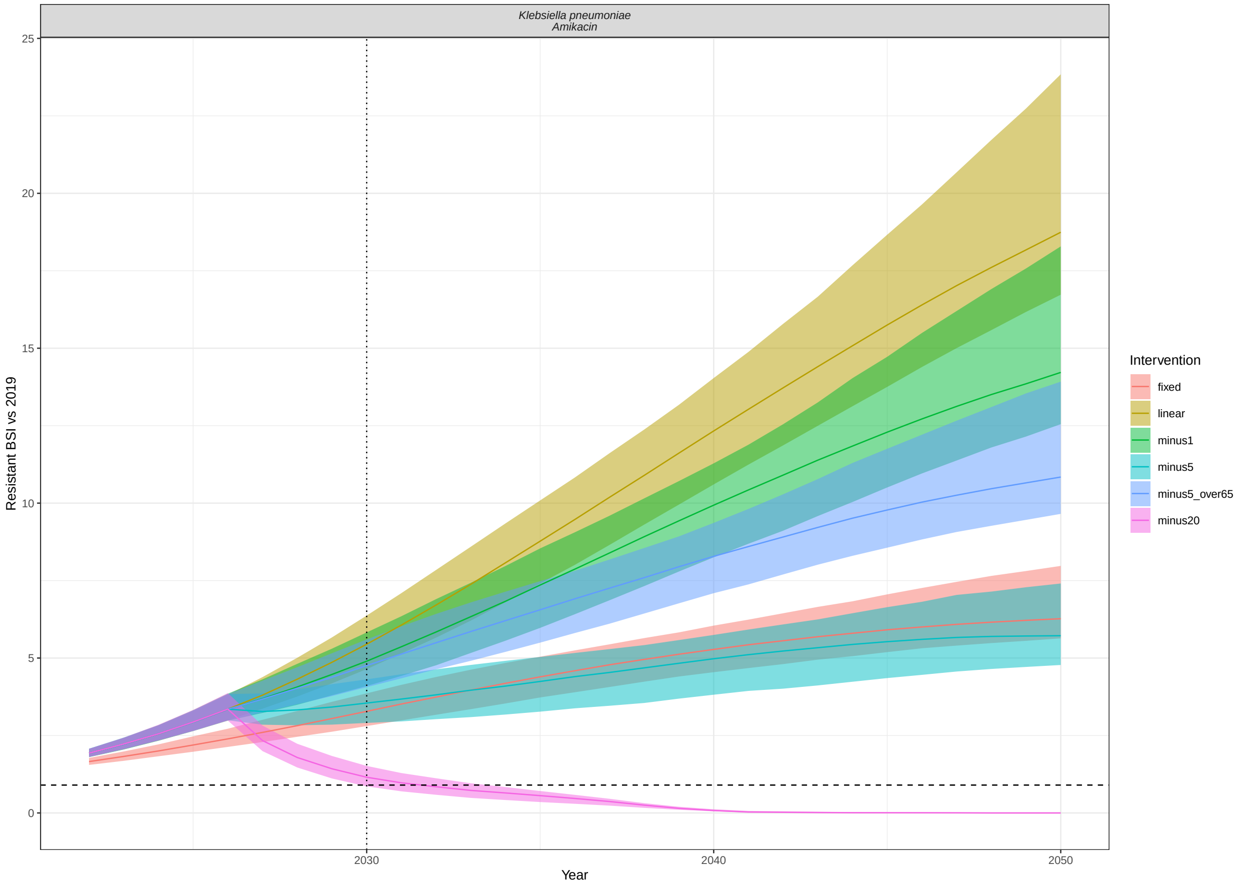

Figure A19: Resistant BSI projections relative to 2019 cases, for each of the intervention scenarios (colour) as in Figure 5. The dashed line is at 0.9 (indicating a 10% relative reduction), and the dotted line at 2030, indicating the UN targets. The line depicts the median and the ribbon the 95% quantiles. The interventions reduce the annual rate of change of BSI incidence by minus 1/5/20 per 100,000 in all ages or in only those aged 65+ (minus5over65). Klebsiella pneumoniae Amikacin.

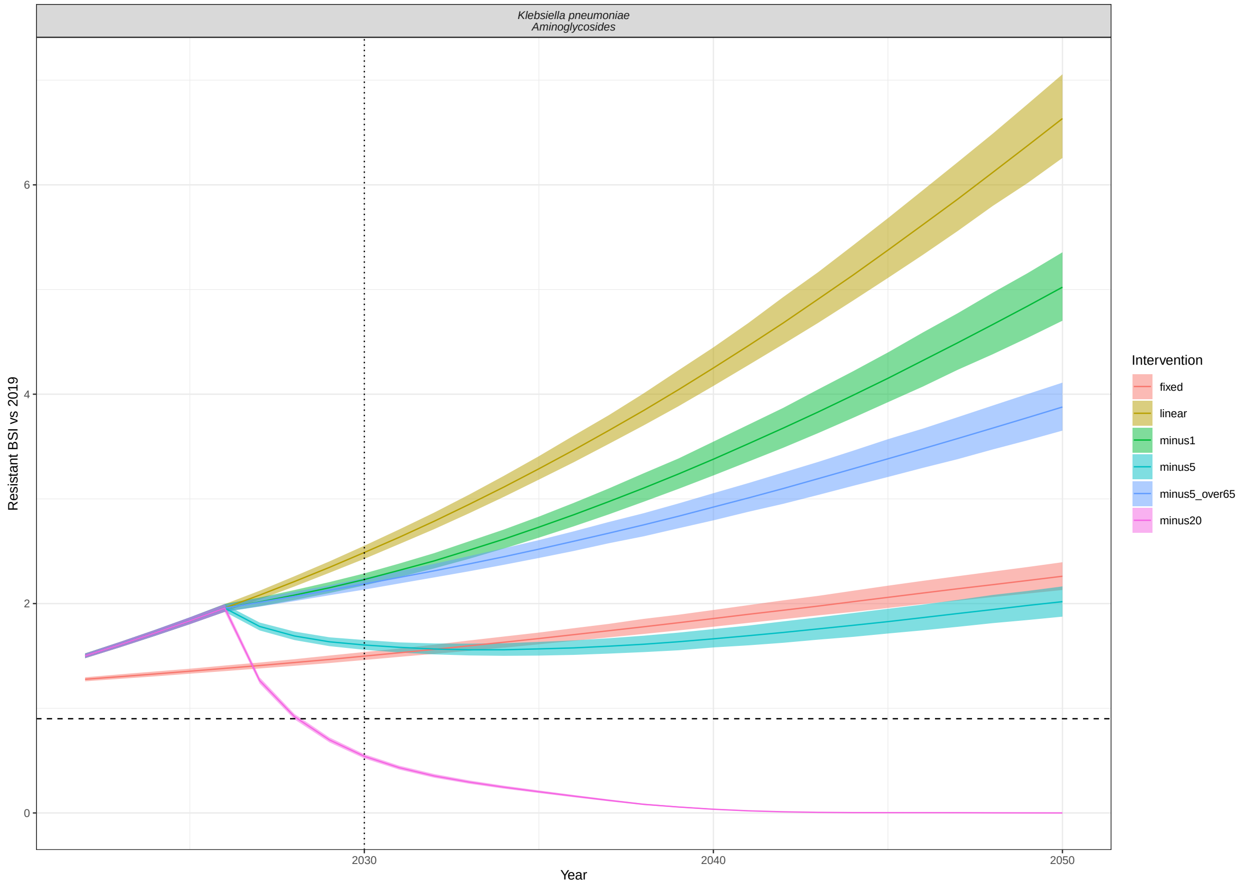

Figure A20: Resistant BSI projections relative to 2019 cases, for each of the intervention scenarios (colour) as in Figure 5. The dashed line is at 0.9 (indicating a 10% relative reduction), and the dotted line at 2030, indicating the UN targets. The line depicts the median and the ribbon the 95% quantiles. The interventions reduce the annual rate of change of BSI incidence by minus 1/5/20 per 100,000 in all ages or in only those aged 65+ (minus5over65). Klebsiella pneumoniae Aminoglycosides.

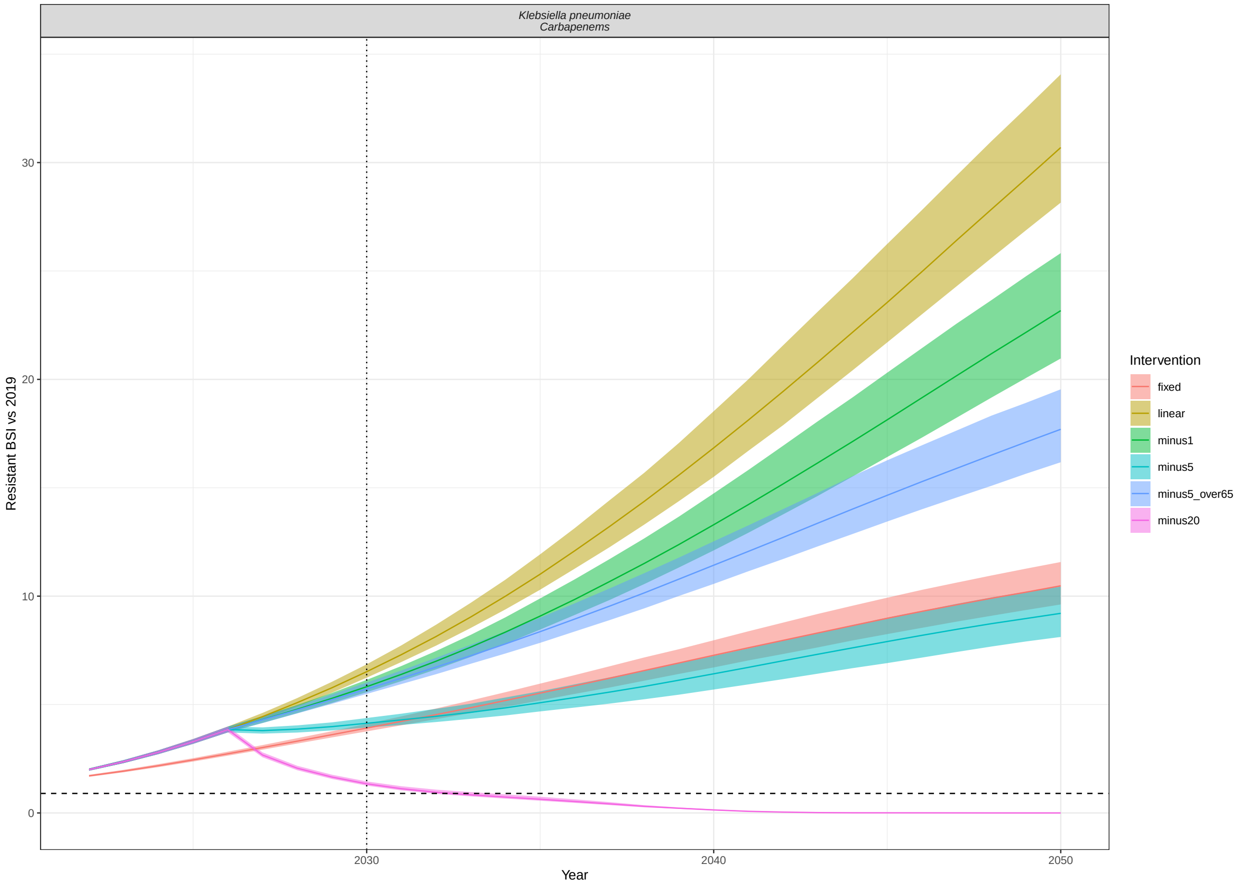

Figure A21: Resistant BSI projections relative to 2019 cases, for each of the intervention scenarios (colour) as in Figure 5. The dashed line is at 0.9 (indicating a 10% relative reduction), and the dotted line at 2030, indicating the UN targets. The line depicts the median and the ribbon the 95% quantiles. The interventions reduce the annual rate of change of BSI incidence by minus 1/5/20 per 100,000 in all ages or in only those aged 65+ (minus5over65). Klebsiella pneumoniae Carbapenems.

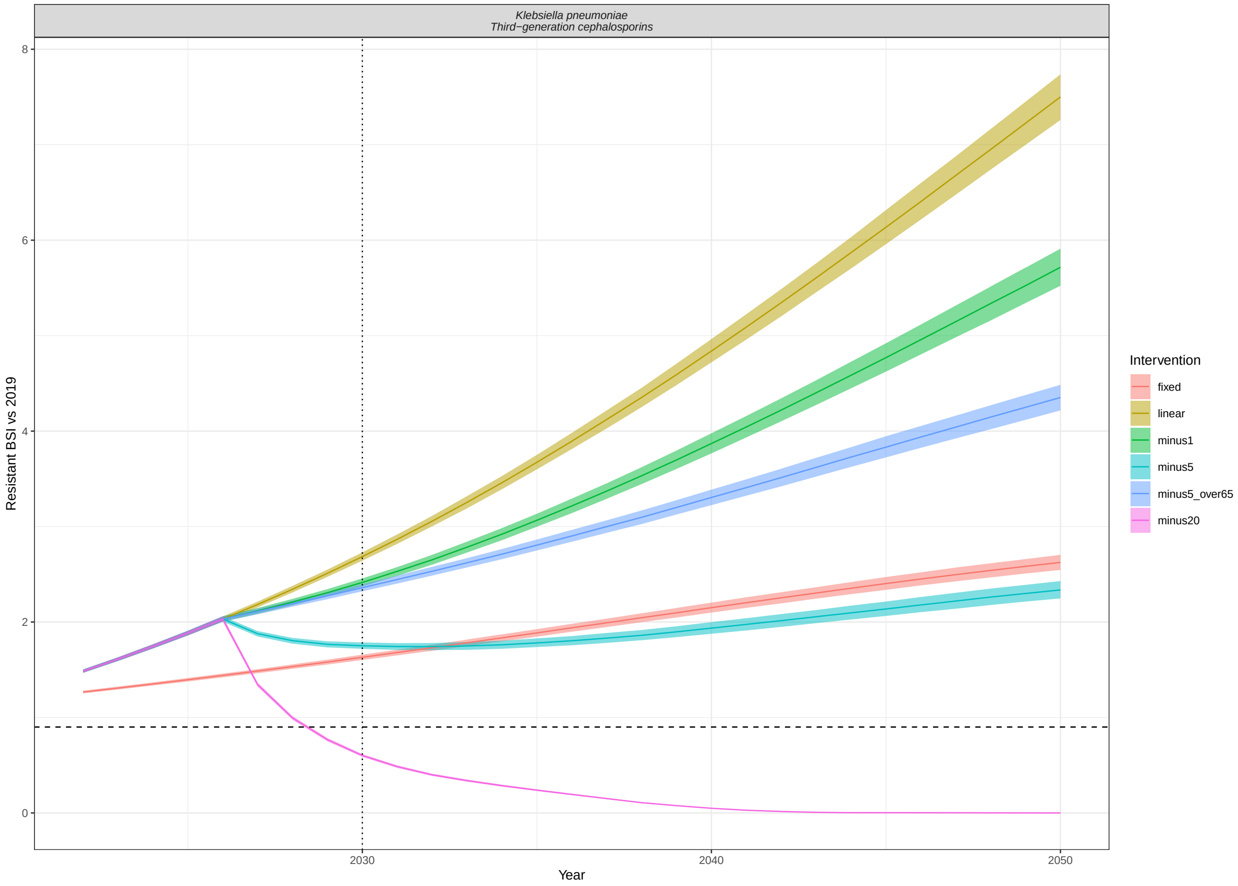

Figure A22: Resistant BSI projections relative to 2019 cases, for each of the intervention scenarios (colour) as in Figure 5. The dashed line is at 0.9 (indicating a 10% relative reduction), and the dotted line at 2030, indicating the UN targets. The line depicts the median and the ribbon the 95% quantiles. The interventions reduce the annual rate of change of BSI incidence by minus 1/5/20 per 100,000 in all ages or in only those aged 65+ (minus5over65). Klebsiella pneumoniae Third-generation cephalosporins.

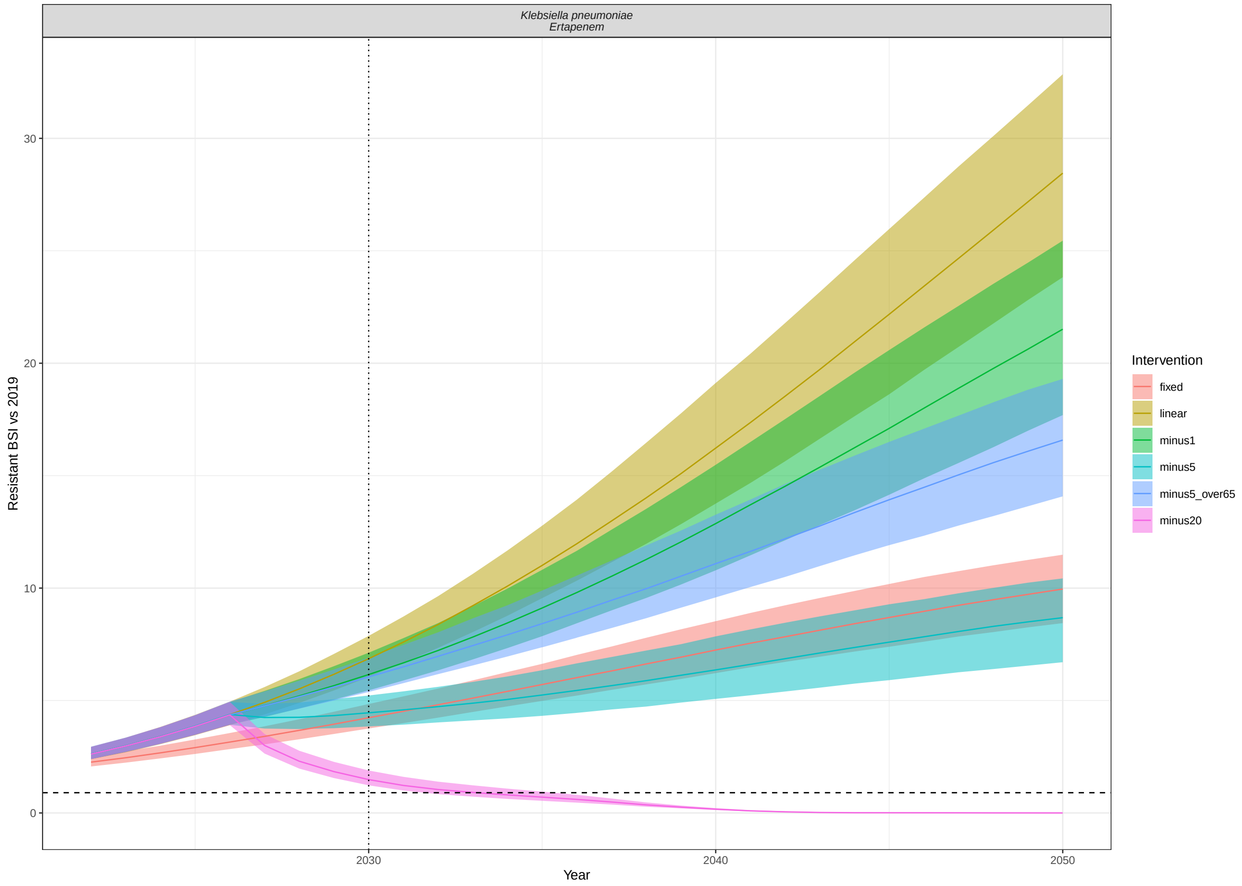

Figure A23: Resistant BSI projections relative to 2019 cases, for each of the intervention scenarios (colour) as in Figure 5. The dashed line is at 0.9 (indicating a 10% relative reduction), and the dotted line at 2030, indicating the UN targets. The line depicts the median and the ribbon the 95% quantiles. The interventions reduce the annual rate of change of BSI incidence by minus 1/5/20 per 100,000 in all ages or in only those aged 65+ (minus5over65). Klebsiella pneumoniae Ertapenem.

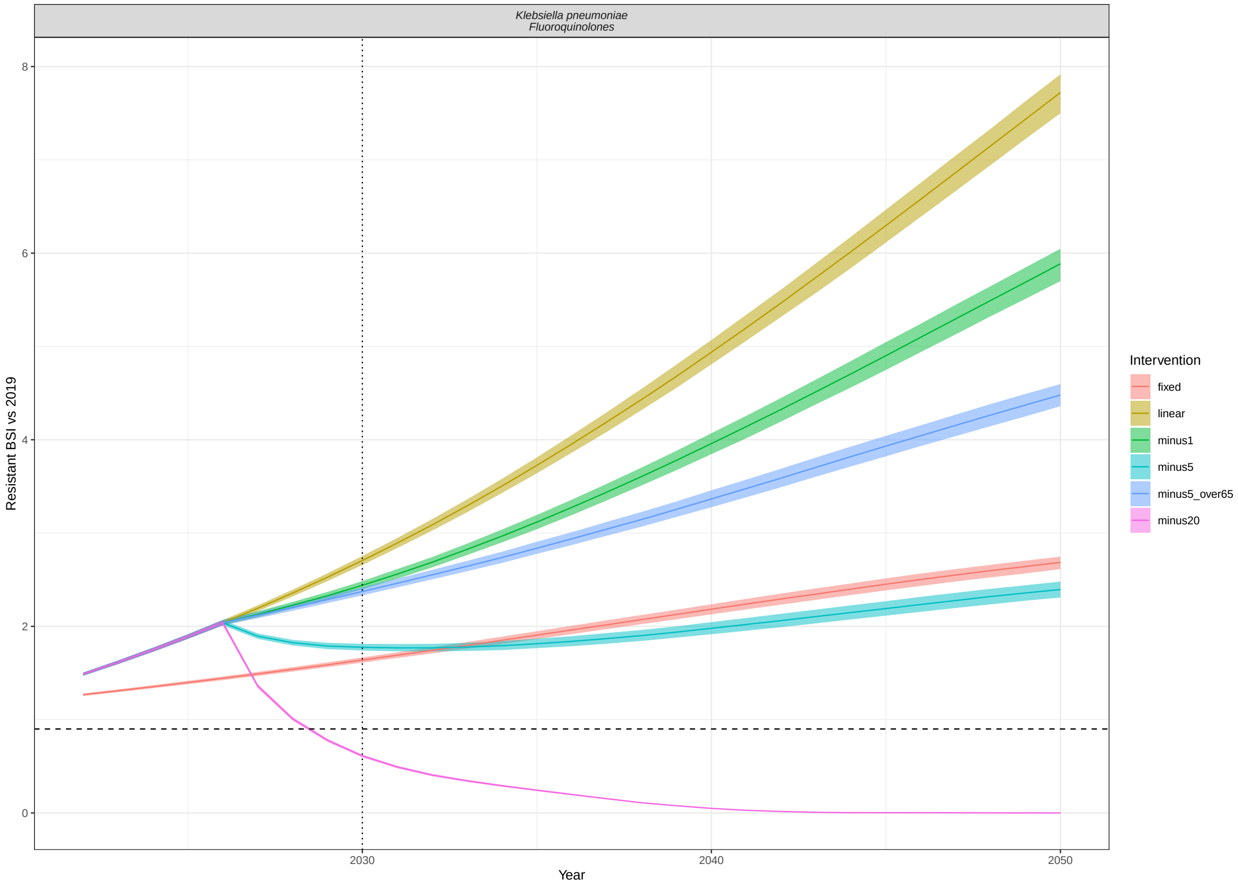

Figure A24: Resistant BSI projections relative to 2019 cases, for each of the intervention scenarios (colour) as in Figure 5. The dashed line is at 0.9 (indicating a 10% relative reduction), and the dotted line at 2030, indicating the UN targets. The line depicts the median and the ribbon the 95% quantiles. The interventions reduce the annual rate of change of BSI incidence by minus 1/5/20 per 100,000 in all ages or in only those aged 65+ (minus5over65). Klebsiella pneumoniae Fluoroquinolones.

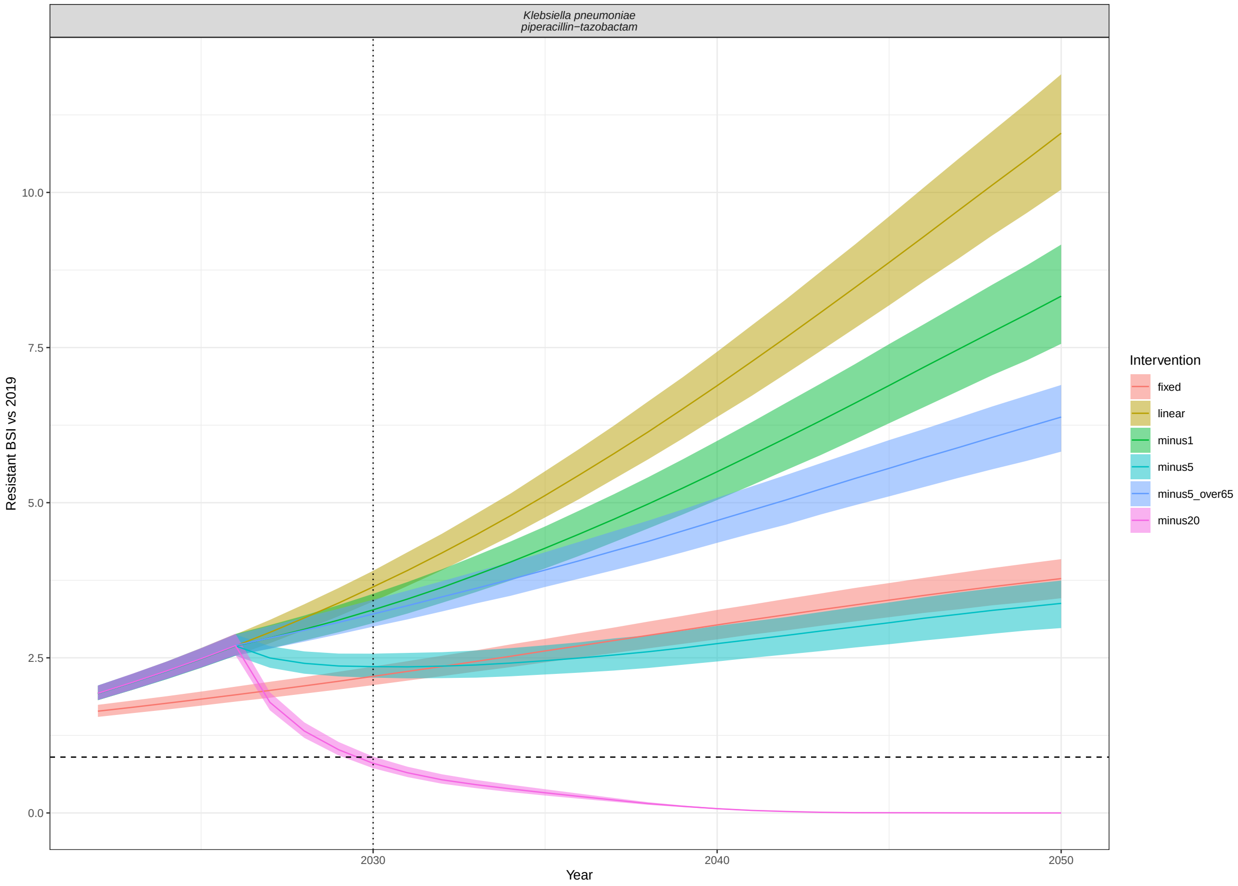

Figure A25: Resistant BSI projections relative to 2019 cases, for each of the intervention scenarios (colour) as in Figure 5. The dashed line is at 0.9 (indicating a 10% relative reduction), and the dotted line at 2030, indicating the UN targets. The line depicts the median and the ribbon the 95% quantiles. The interventions reduce the annual rate of change of BSI incidence by minus 1/5/20 per 100,000 in all ages or in only those aged 65+ (minus5over65). Klebsiella pneumoniae piperacillin-tazobactam.

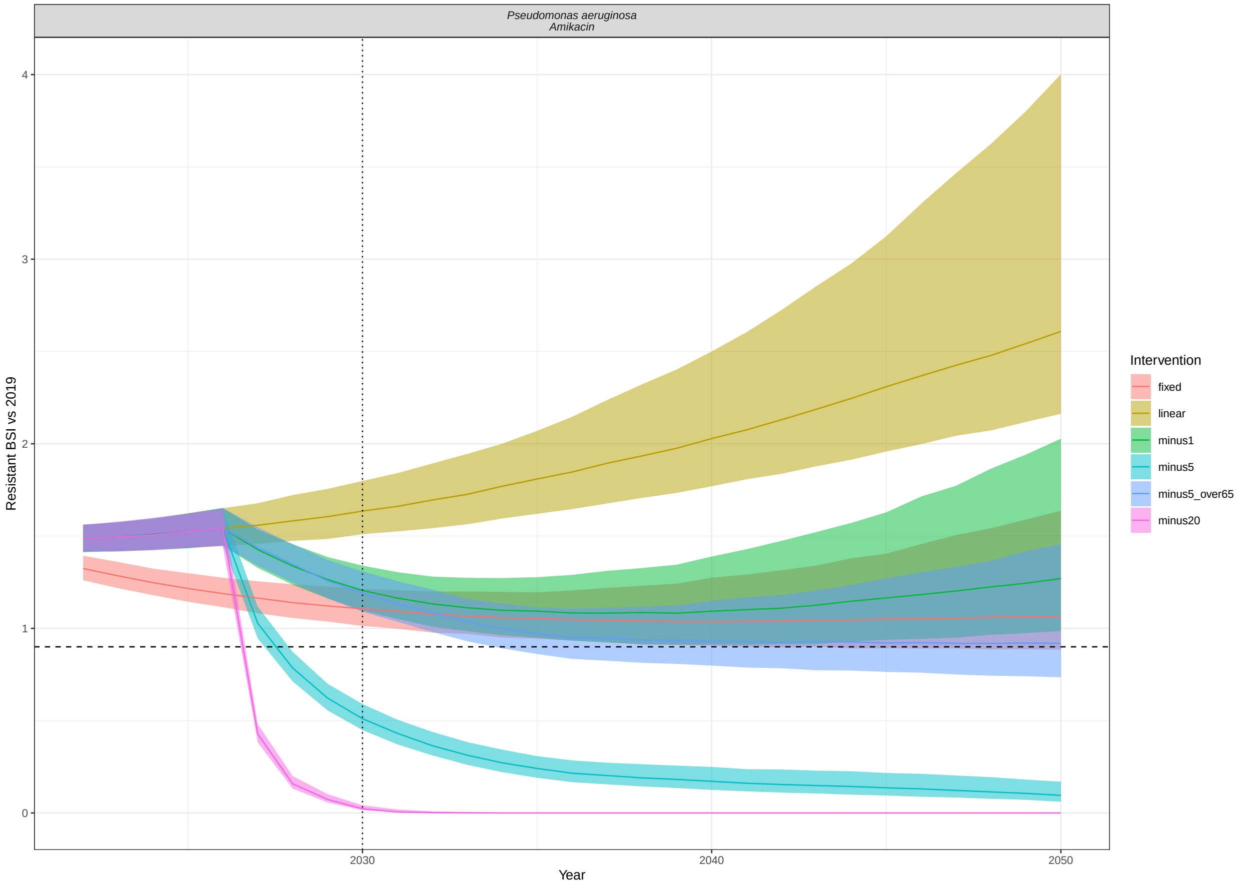

Figure A26: Resistant BSI projections relative to 2019 cases, for each of the intervention scenarios (colour) as in Figure 5. The dashed line is at 0.9 (indicating a 10% relative reduction), and the dotted line at 2030, indicating the UN targets. The line depicts the median and the ribbon the 95% quantiles. The interventions reduce the annual rate of change of BSI incidence by minus 1/5/20 per 100,000 in all ages or in only those aged 65+ (minus5over65). Pseudomonas aeruginosa Amikacin.

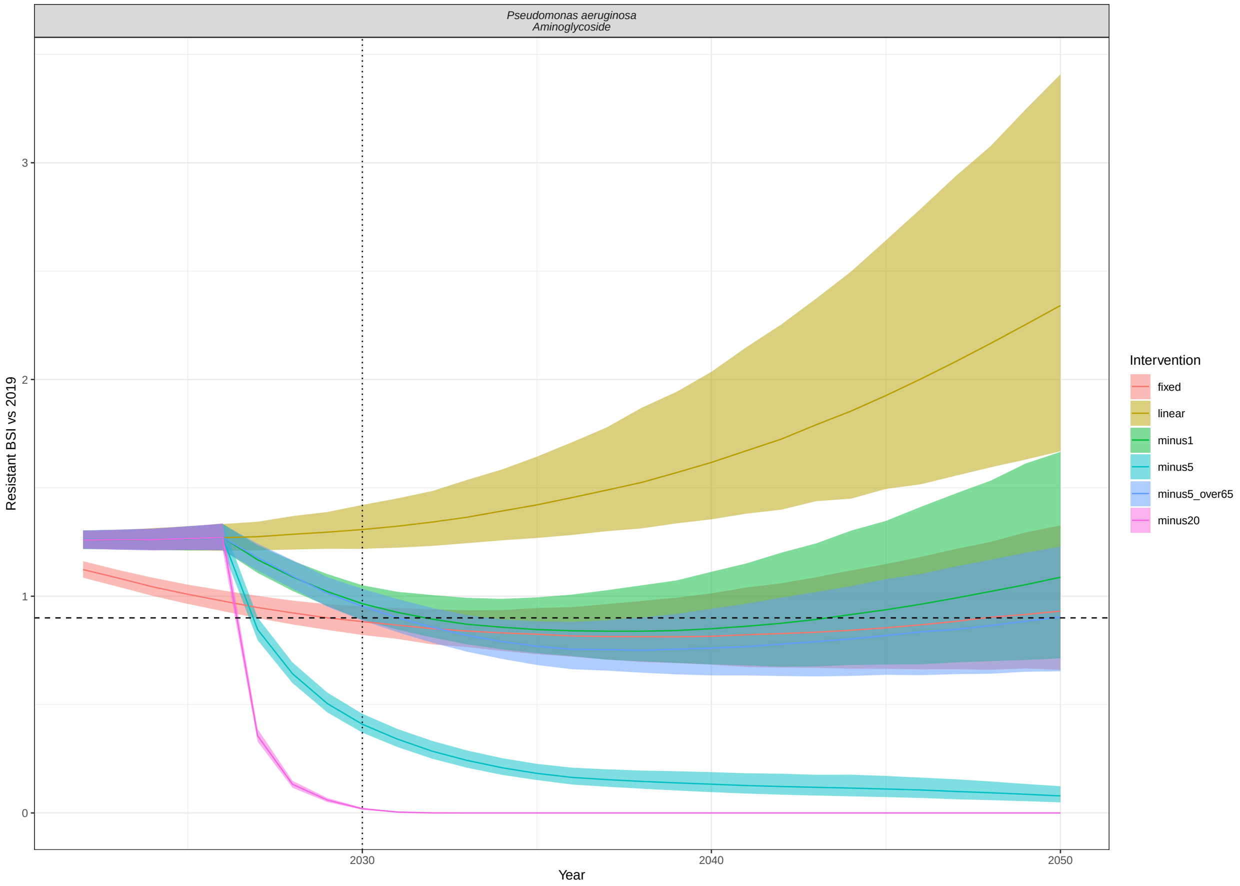

Figure A27: Resistant BSI projections relative to 2019 cases, for each of the intervention scenarios (colour) as in Figure 5. The dashed line is at 0.9 (indicating a 10% relative reduction), and the dotted line at 2030, indicating the UN targets. The line depicts the median and the ribbon the 95% quantiles. The interventions reduce the annual rate of change of BSI incidence by minus 1/5/20 per 100,000 in all ages or in only those aged 65+ (minus5over65). Pseudomonas aeruginosa Aminoglycoside.

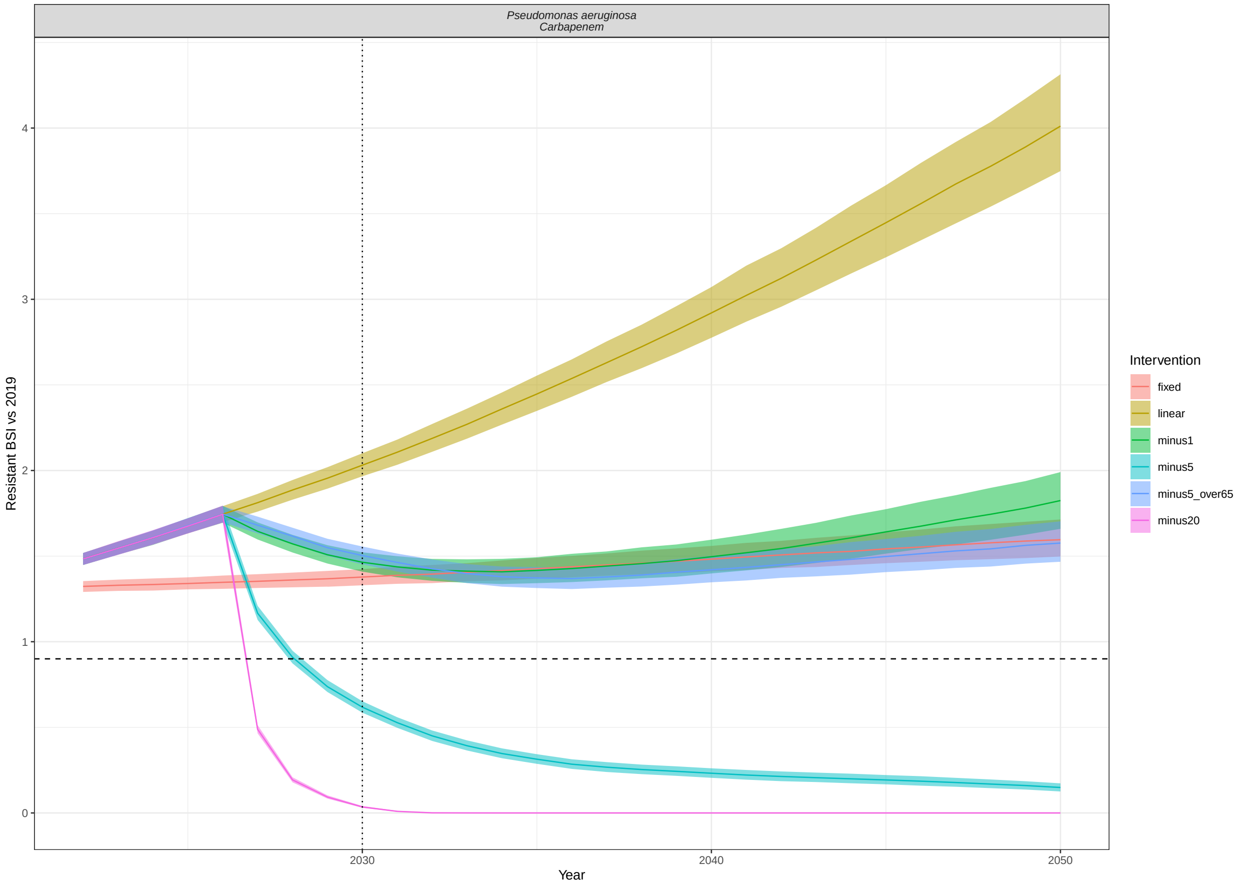

Figure A28: Resistant BSI projections relative to 2019 cases, for each of the intervention scenarios (colour) as in Figure 5. The dashed line is at 0.9 (indicating a 10% relative reduction), and the dotted line at 2030, indicating the UN targets. The line depicts the median and the ribbon the 95% quantiles. The interventions reduce the annual rate of change of BSI incidence by minus 1/5/20 per 100,000 in all ages or in only those aged 65+ (minus5over65). Pseudomonas aeruginosa Carbapenem.

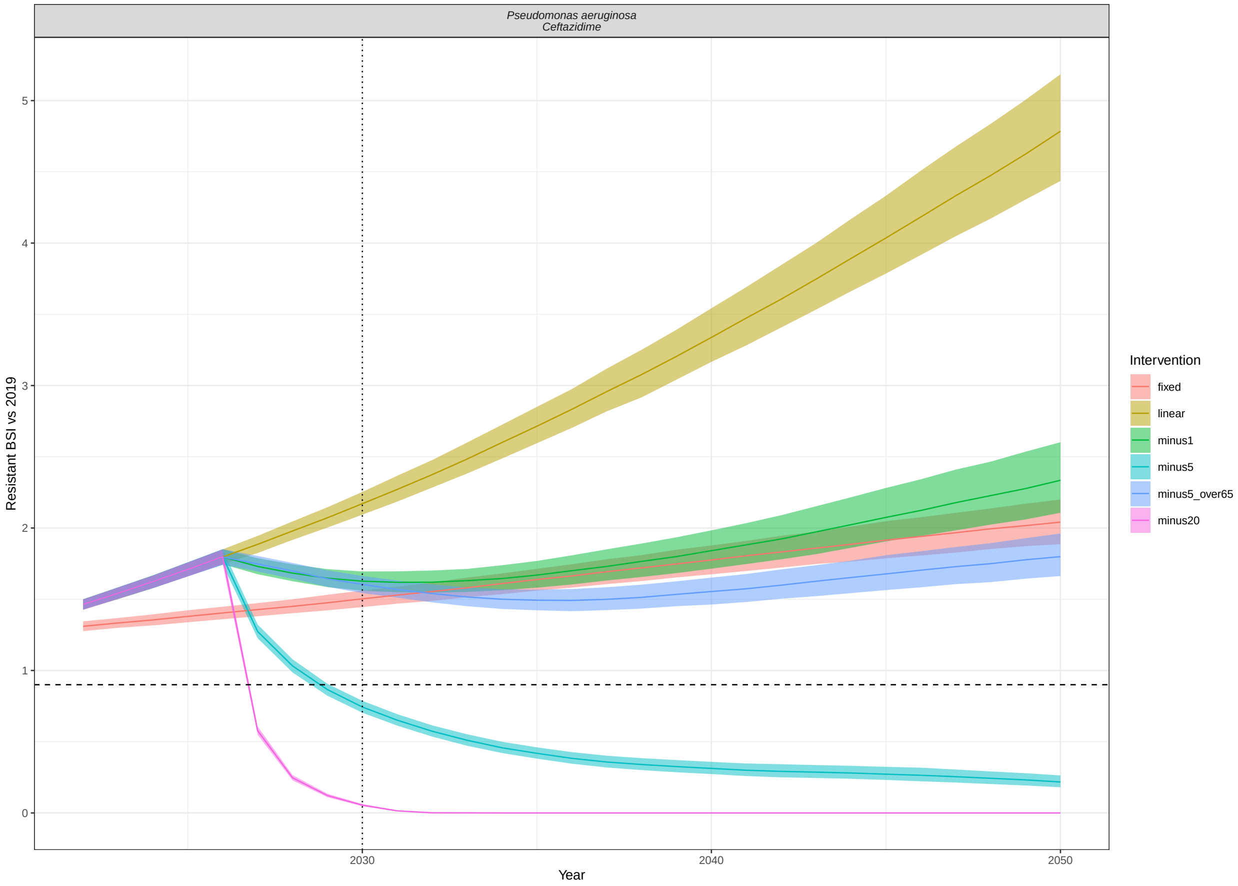

Figure A29: Resistant BSI projections relative to 2019 cases, for each of the intervention scenarios (colour) as in Figure 5. The dashed line is at 0.9 (indicating a 10% relative reduction), and the dotted line at 2030, indicating the UN targets. The line depicts the median and the ribbon the 95% quantiles. The interventions reduce the annual rate of change of BSI incidence by minus 1/5/20 per 100,000 in all ages or in only those aged 65+ (minus5over65). Pseudomonas aeruginosa Ceftazidime.

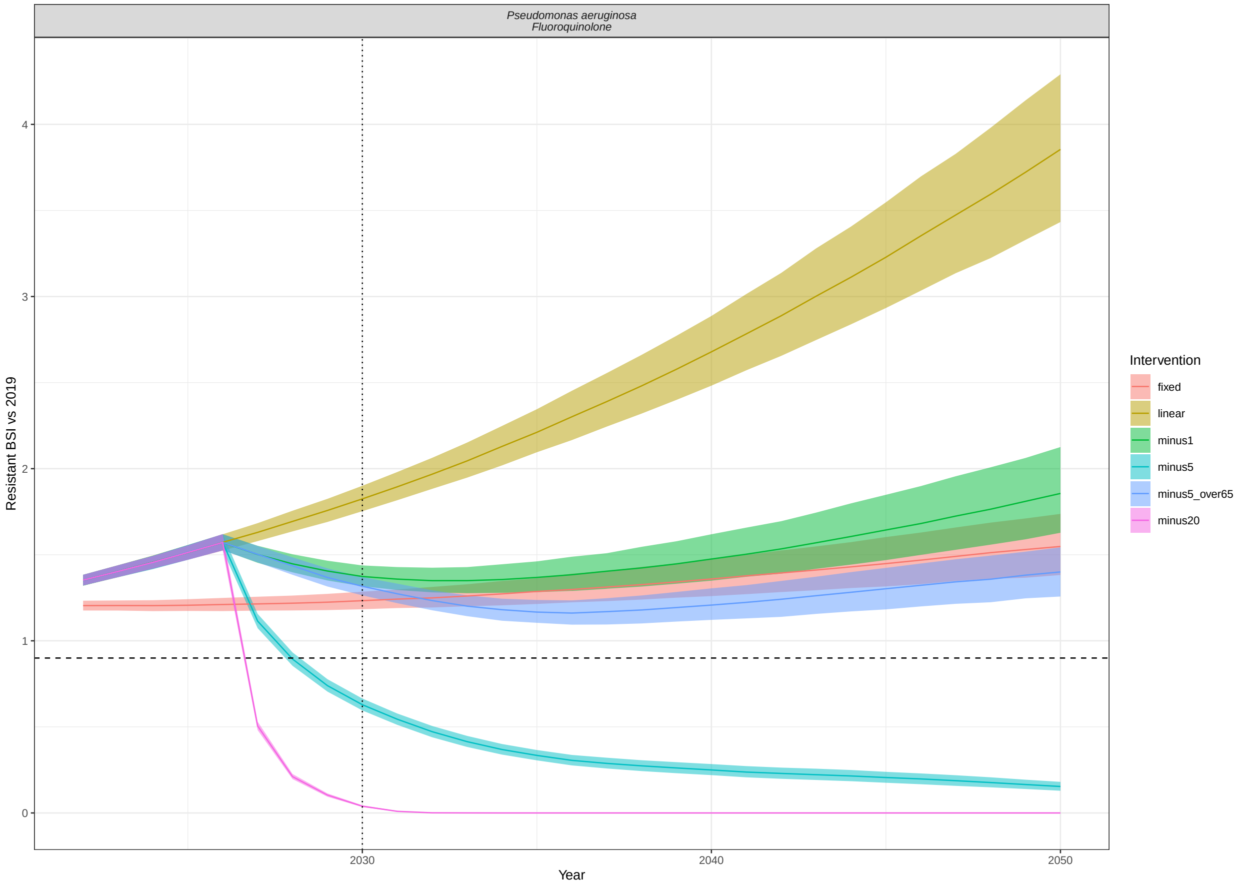

Figure A30: Resistant BSI projections relative to 2019 cases, for each of the intervention scenarios (colour) as in Figure 5. The dashed line is at 0.9 (indicating a 10% relative reduction), and the dotted line at 2030, indicating the UN targets. The line depicts the median and the ribbon the 95% quantiles. The interventions reduce the annual rate of change of BSI incidence by minus 1/5/20 per 100,000 in all ages or in only those aged 65+ (minus5over65). Pseudomonas aeruginosa Fluoroquinolone.

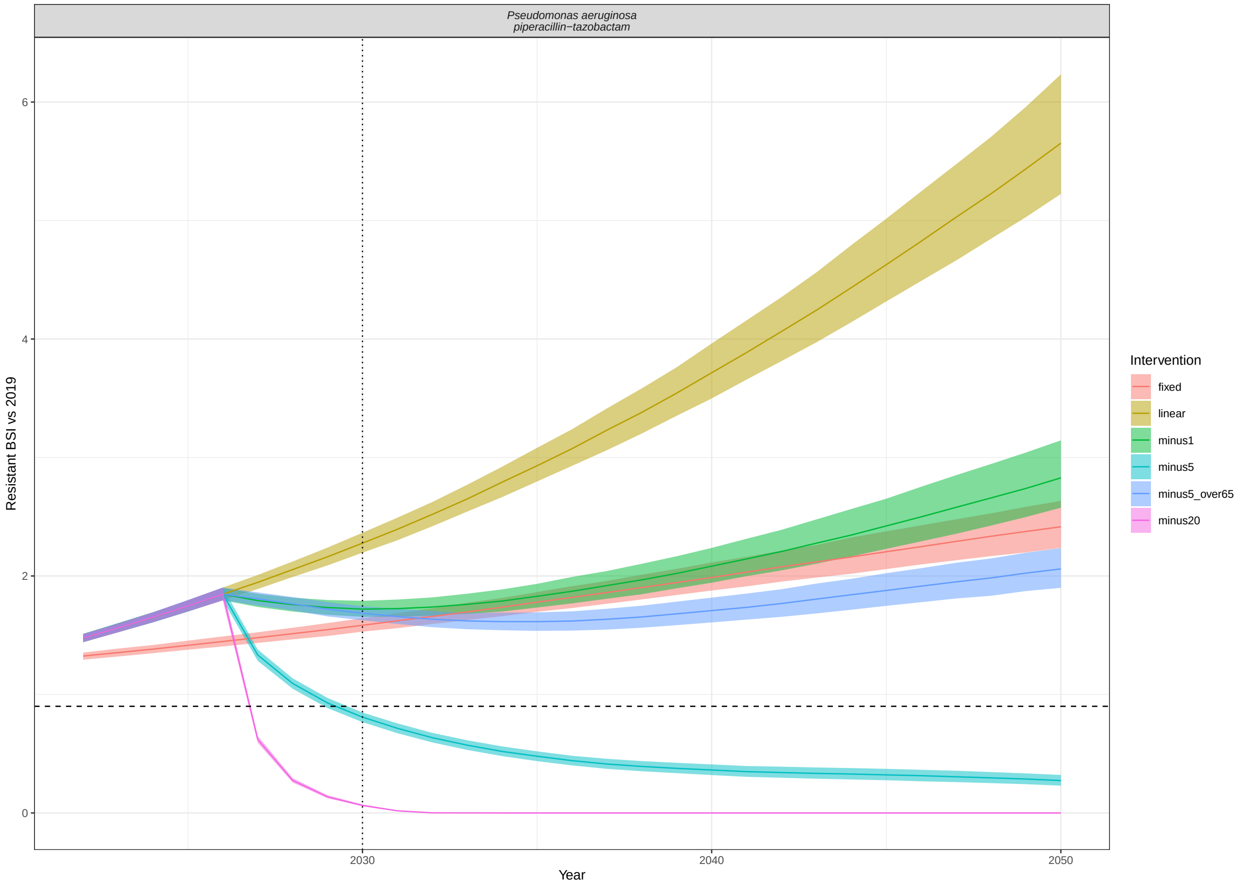

Figure A31: Resistant BSI projections relative to 2019 cases, for each of the intervention scenarios (colour) as in Figure 5. The dashed line is at 0.9 (indicating a 10% relative reduction), and the dotted line at 2030, indicating the UN targets. The line depicts the median and the ribbon the 95% quantiles. The interventions reduce the annual rate of change of BSI incidence by minus 1/5/20 per 100,000 in all ages or in only those aged 65+ (minus5over65). Pseudomonas aeruginosa piperacillin-tazobactam.

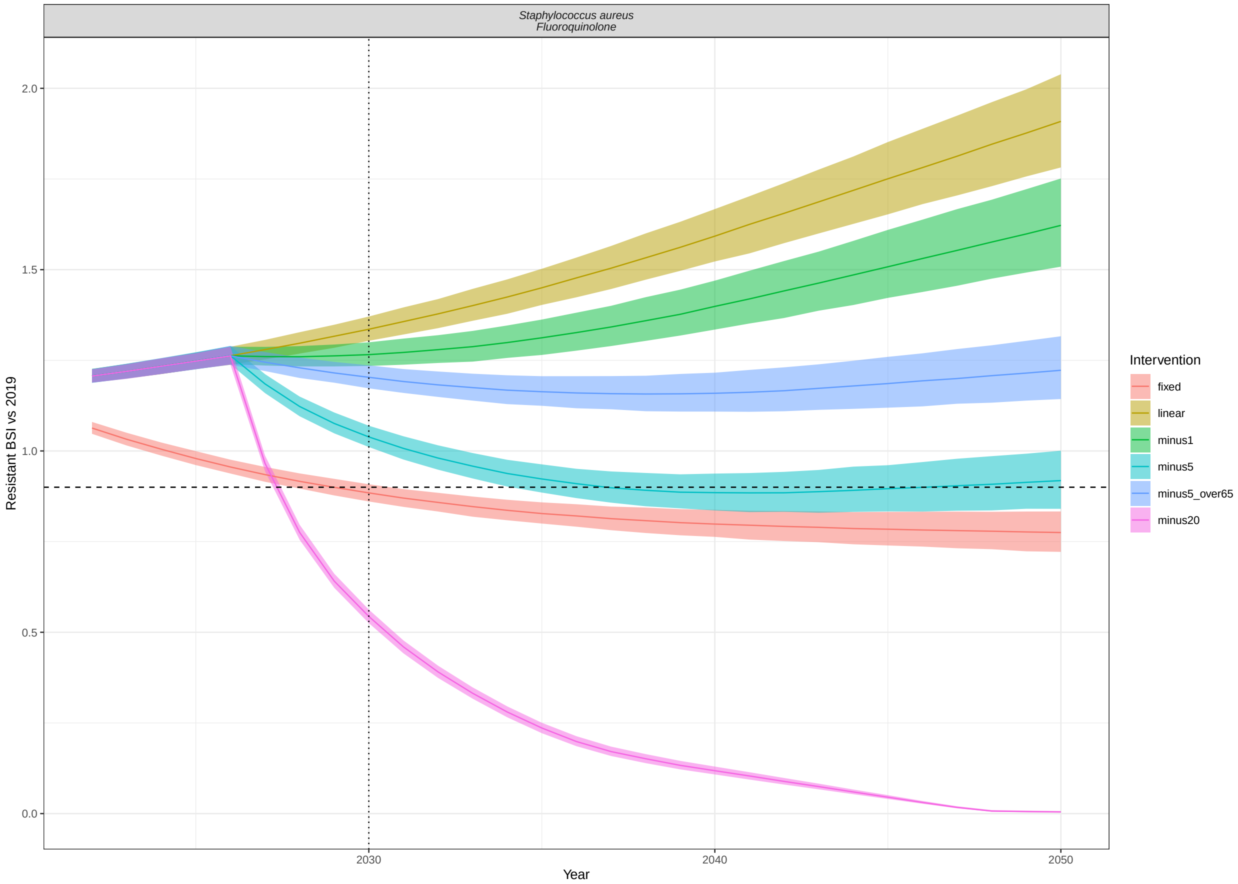

Figure A32: Resistant BSI projections relative to 2019 cases, for each of the intervention scenarios (colour) as in Figure 5. The dashed line is at 0.9 (indicating a 10% relative reduction), and the dotted line at 2030, indicating the UN targets. The line depicts the median and the ribbon the 95% quantiles. The interventions reduce the annual rate of change of BSI incidence by minus 1/5/20 per 100,000 in all ages or in only those aged 65+ (minus5over65). Staphylococcus aureus Fluoroquinolone.

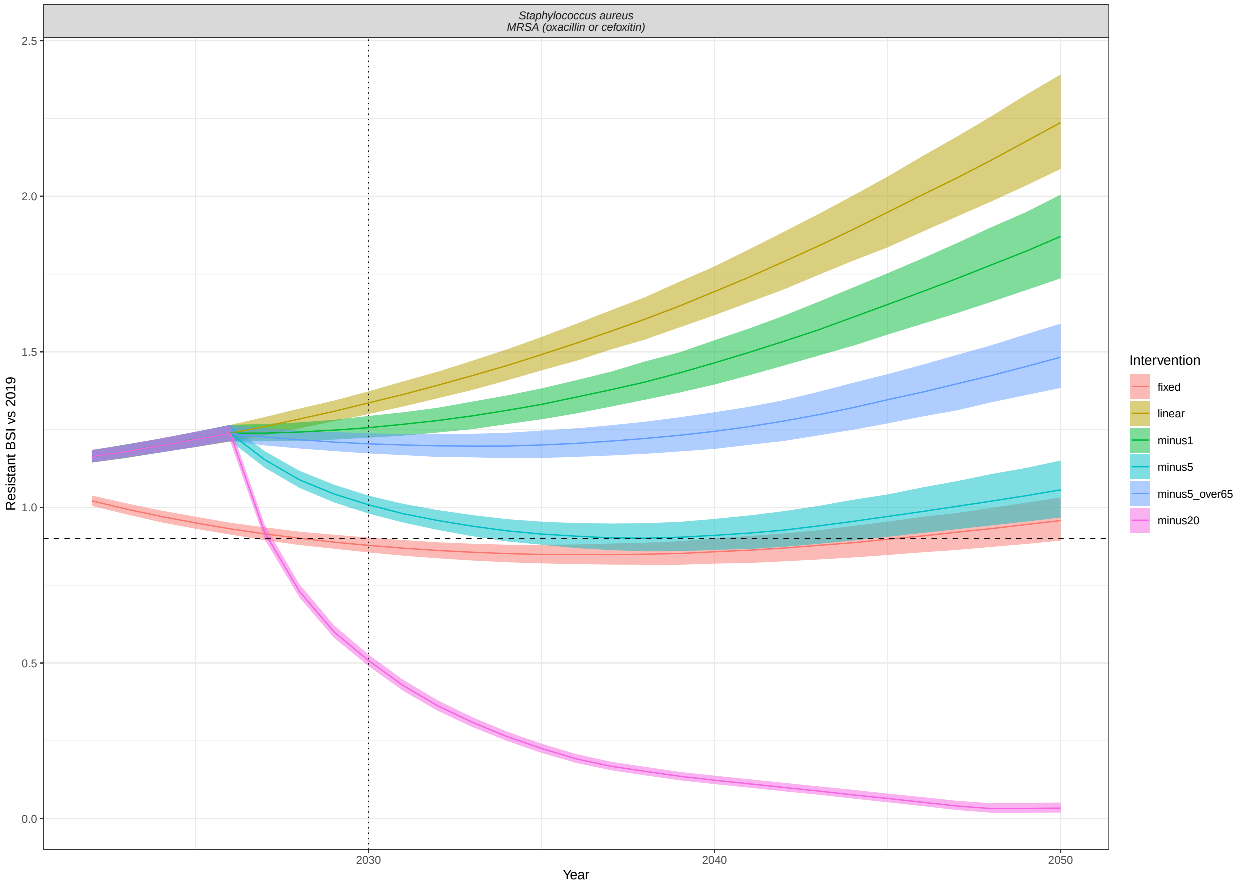

Figure A33: Resistant BSI projections relative to 2019 cases, for each of the intervention scenarios (colour) as in Figure 5. The dashed line is at 0.9 (indicating a 10% relative reduction), and the dotted line at 2030, indicating the UN targets. The line depicts the median and the ribbon the 95% quantiles. The interventions reduce the annual rate of change of BSI incidence by minus 1/5/20 per 100,000 in all ages or in only those aged 65+ (minus5over65). Staphylococcus aureus MRSA (oxacillin or cefoxitin).

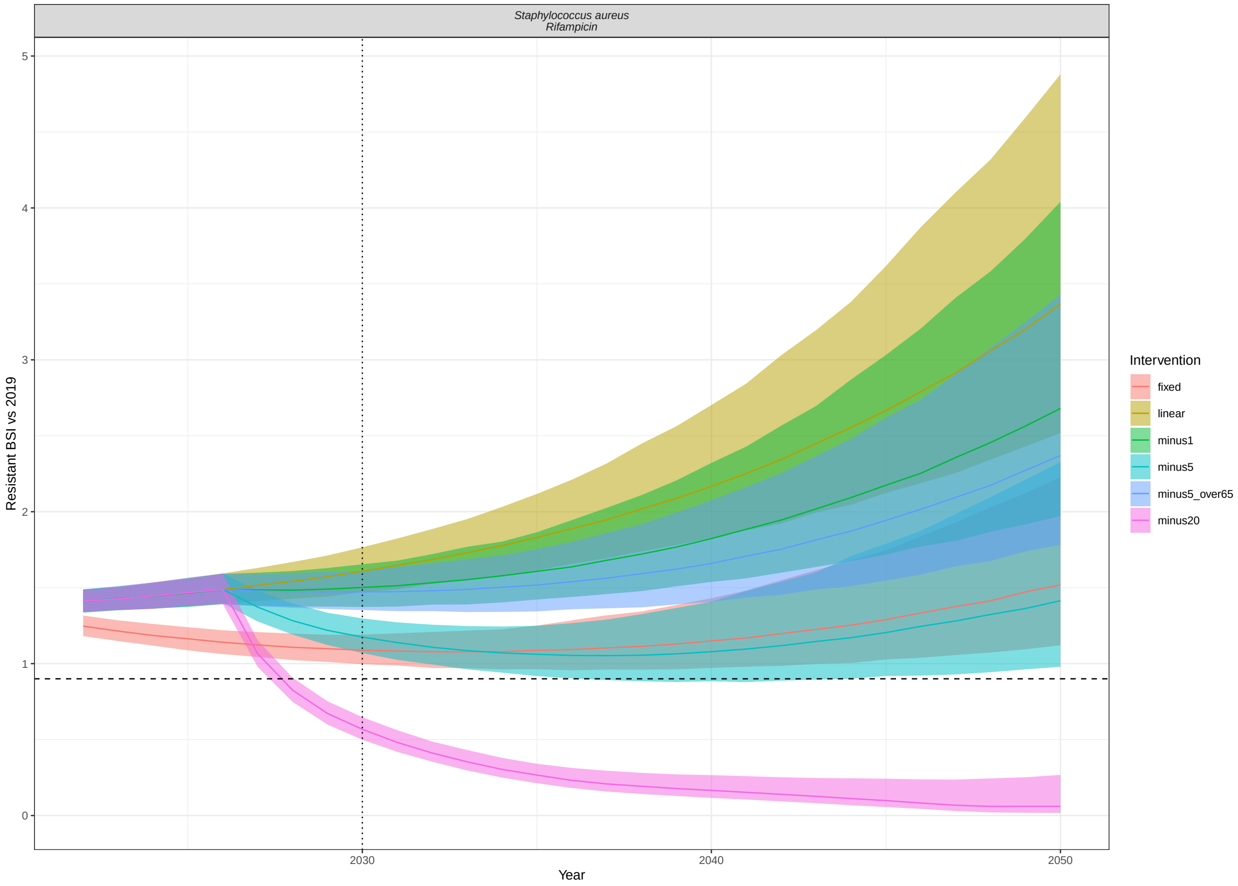

Figure A34: Resistant BSI projections relative to 2019 cases, for each of the intervention scenarios (colour) as in Figure 5. The dashed line is at 0.9 (indicating a 10% relative reduction), and the dotted line at 2030, indicating the UN targets. The line depicts the median and the ribbon the 95% quantiles. The interventions reduce the annual rate of change of BSI incidence by minus 1/5/20 per 100,000 in all ages or in only those aged 65+ (minus5over65). Staphylococcus aureus Rifampicin.

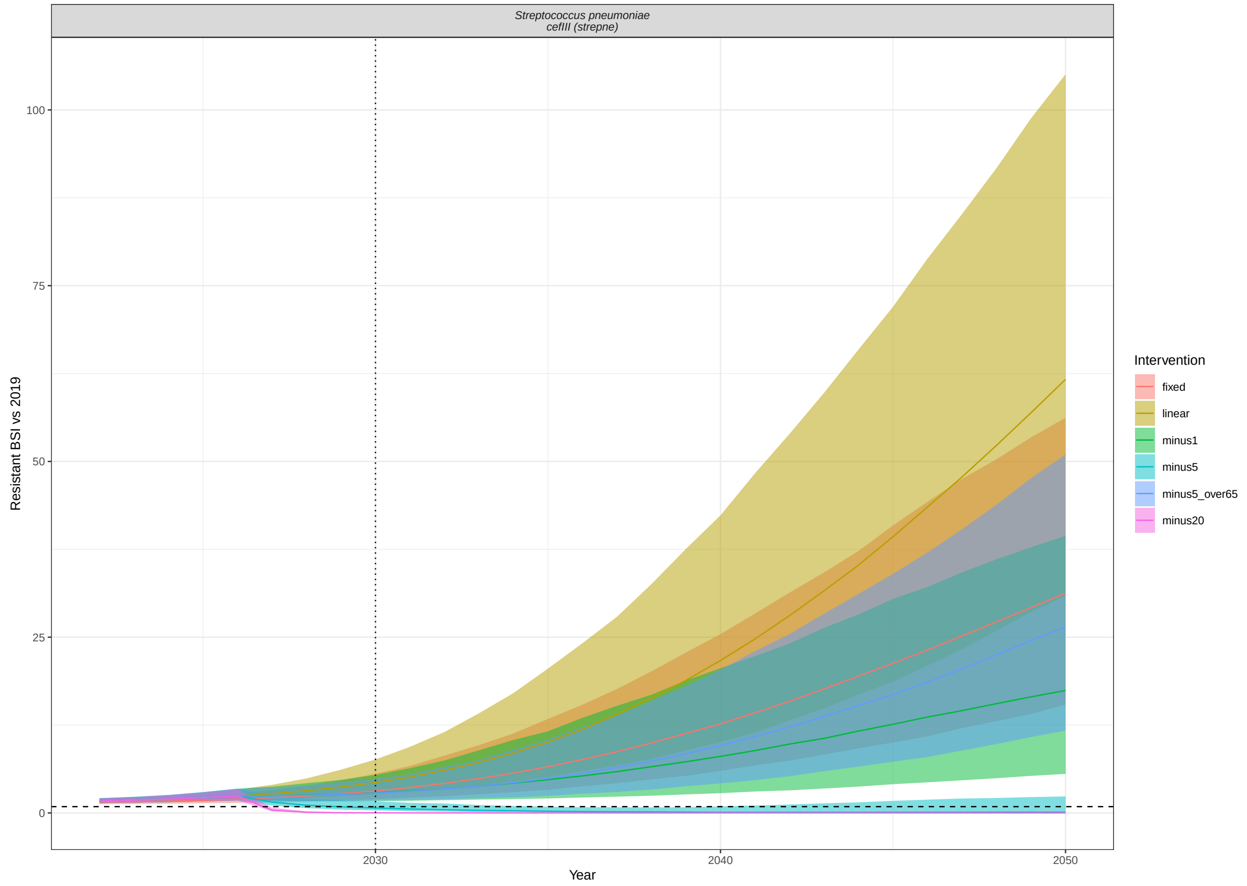

Figure A35: Resistant BSI projections relative to 2019 cases, for each of the intervention scenarios (colour) as in Figure 5. The dashed line is at 0.9 (indicating a 10% relative reduction), and the dotted line at 2030, indicating the UN targets. The line depicts the median and the ribbon the 95% quantiles. The interventions reduce the annual rate of change of BSI incidence by minus 1/5/20 per 100,000 in all ages or in only those aged 65+ (minus5over65). Streptococcus pneumoniae cefIII (strepne).

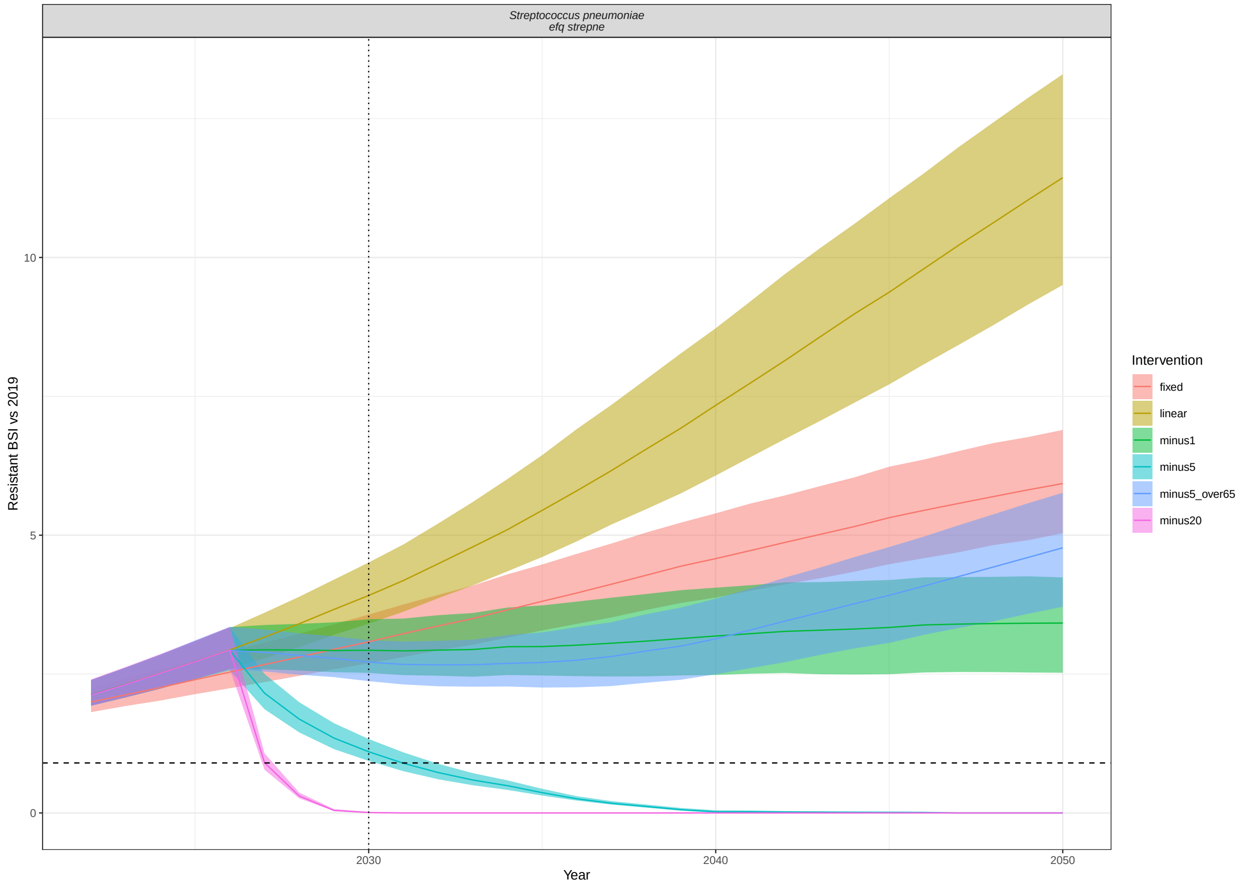

Figure A36: Resistant BSI projections relative to 2019 cases, for each of the intervention scenarios (colour) as in Figure 5. The dashed line is at 0.9 (indicating a 10% relative reduction), and the dotted line at 2030, indicating the UN targets. The line depicts the median and the ribbon the 95% quantiles. The interventions reduce the annual rate of change of BSI incidence by minus 1/5/20 per 100,000 in all ages or in only those aged 65+ (minus5over65). Streptococcus pneumoniae efq strepne.

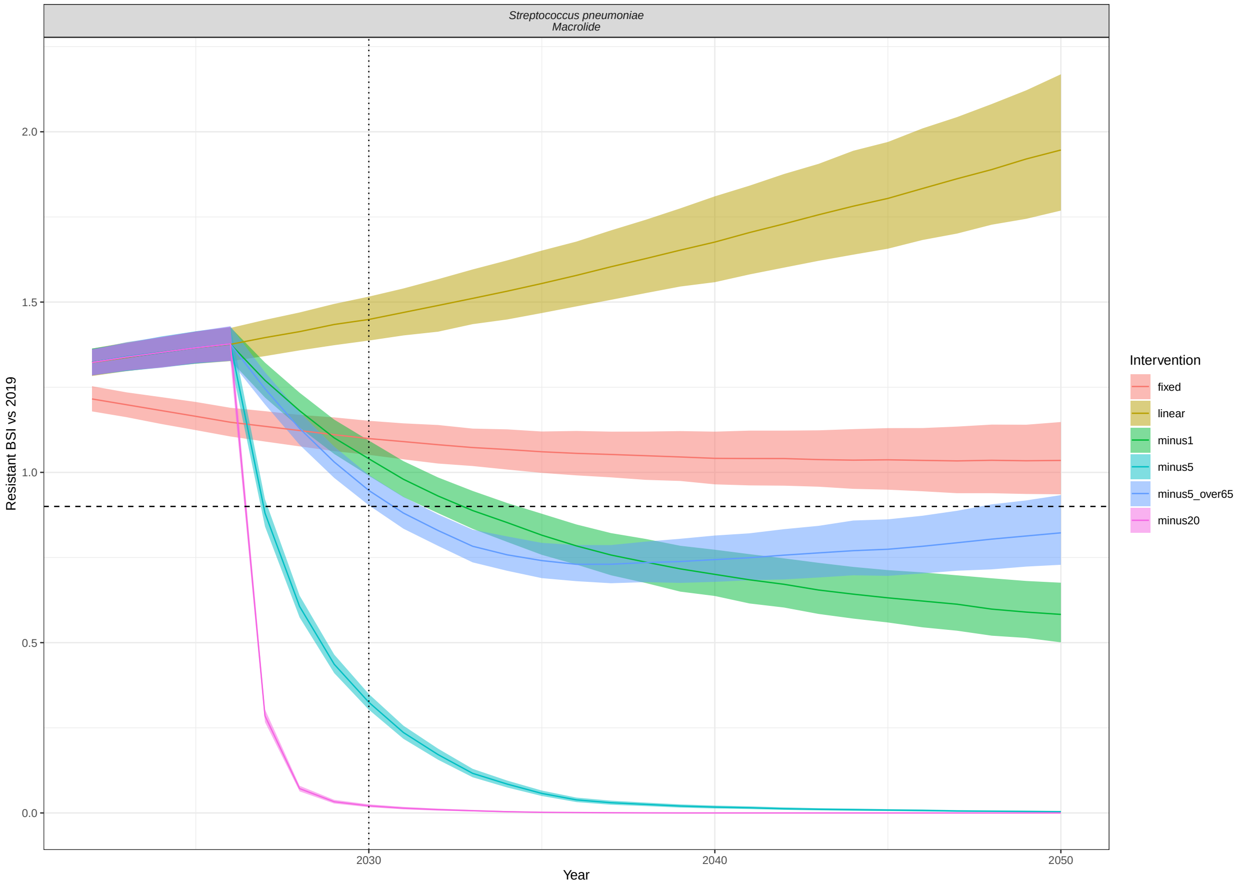

Figure A37: Resistant BSI projections relative to 2019 cases, for each of the intervention scenarios (colour) as in Figure 5. The dashed line is at 0.9 (indicating a 10% relative reduction), and the dotted line at 2030, indicating the UN targets. The line depicts the median and the ribbon the 95% quantiles. The interventions reduce the annual rate of change of BSI incidence by minus 1/5/20 per 100,000 in all ages or in only those aged 65+ (minus5over65). Streptococcus pneumoniae Macrolide.

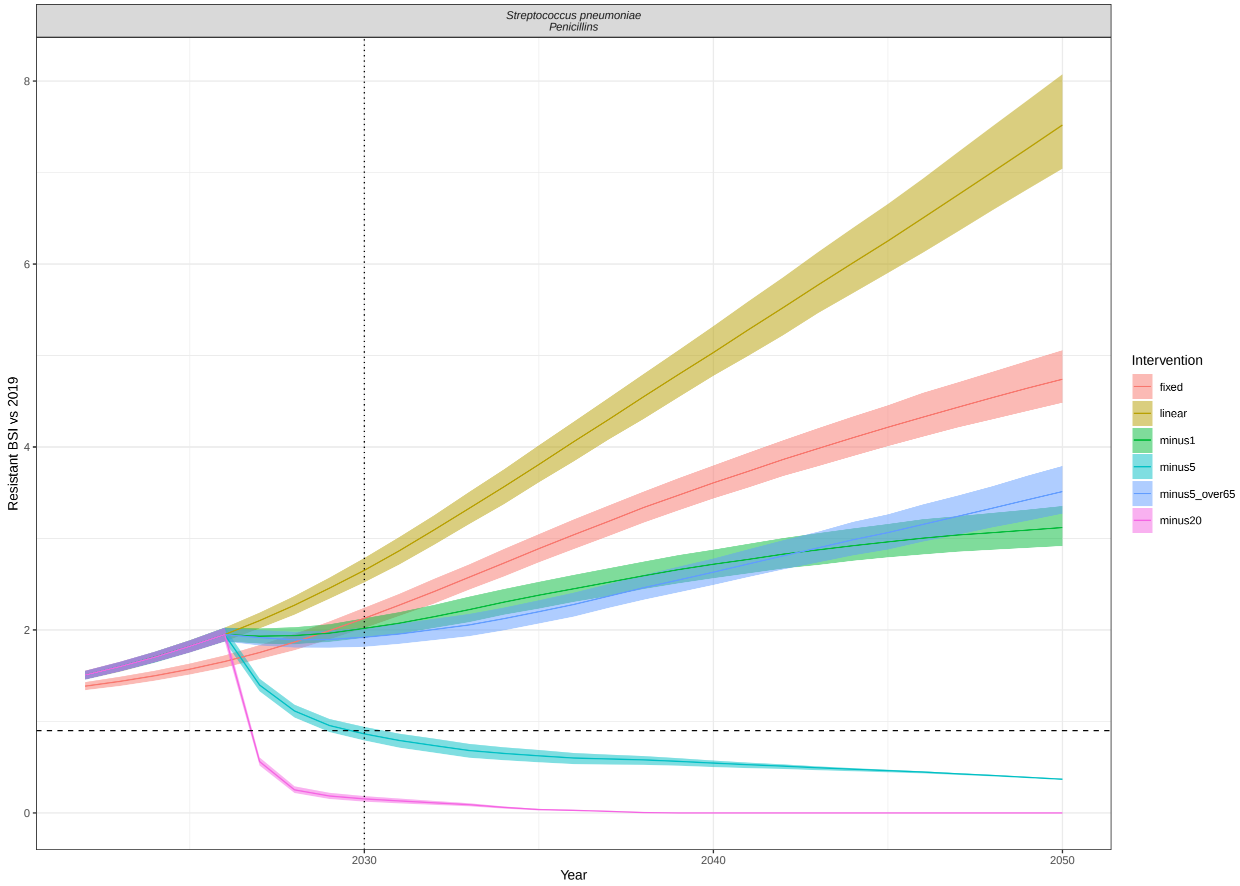

Figure A38: Resistant BSI projections relative to 2019 cases, for each of the intervention scenarios (colour) as in Figure 5. The dashed line is at 0.9 (indicating a 10% relative reduction), and the dotted line at 2030, indicating the UN targets. The line depicts the median and the ribbon the 95% quantiles. The interventions reduce the annual rate of change of BSI incidence by minus 1/5/20 per 100,000 in all ages or in only those aged 65+ (minus5over65). Streptococcus pneumoniae Penicillins.
