# Supplementary material for: Combining demographic shifts with age-based resistance prevalence to estimate future antimicrobial resistance burden in Europe and implications for targets: A modelling study
Source: PLoS Med. 2025 Nov 4;22(11):e1004579. doi: 10.1371/journal.pmed.1004579 (PMC12585039; doi:10.1371/journal.pmed.1004579)
Supplement: Attachment — Submitted filename: 2507 Response to reviewers.docx [file pmed.1004579.s006.docx]

Dear Dr Farrell,

Thank you for the opportunity to revise and resubmit our manuscript. Please find attached a clean and track-changes version of the manuscript. Our response to reviewers is below. Line numbers refer to those in the cleaned version of the manuscript.

We have also included an Author Summary in the manuscript and provided figures in the correct format.

On behalf of all co-authors,

Gwen

--------

Dear Dr Knight,

Many thanks for submitting your manuscript "Combining demographic shifts with age-based resistance prevalence: a modelling estimate of future AMR burden in Europe and implications for targets" (PMEDICINE-D-25-00946R1) to PLOS Medicine. The paper has been reviewed by subject experts and a statistician; their comments are included below and can also be accessed here:

As you will see, the reviewers find the work interesting and potentially important for predicting future AMR trends. After discussing the paper with the editorial team and an academic editor with relevant expertise, I'm pleased to invite you to revise the paper in response to the reviewers' comments. We plan to send the revised paper to some or all of the original reviewers, and we cannot provide any guarantees at this stage regarding publication. We ask that you address the comments of the statistical reviewer (#3), clarify the rationale for the antibiotic-bacteria combinations chosen, and consider the potential effects of changing patterns of diagnosis in the elderly. Please provide an Author Summary with bullet points (the last point should briefly state the study limitations), and please include an email address for the contact for EARS-Net.

In addition to these revisions, you will need to complete some formatting changes, which you will receive in a follow up email. A member of our team will be in touch with a set of requests shortly.

When you upload your revision, please include a point-by-point response that addresses all of the reviewer and editorial points, indicating the changes made in the manuscript and either an excerpt of the revised text or the location (eg: page and line number) where each change can be found. Please also be sure to check the general editorial comments at the end of this letter and include these in your point-by-point response. When you resubmit your paper, please include a clean version of the paper as the main article file and a version with changes tracked as a marked-up manuscript. It may also be helpful to check the guidelines for revised papers at [http://journals.plos.org/plosmedicine/s/revising-your-manuscript](http://url.uk.m.mimecastprotect.com/s/wiGdCR1NGcXY3EOtNhwh1EWQm?domain=journals.plos.org) for any that apply to your paper.

We ask that you submit your revision by Jul 15 2025 11:59PM. However, if this deadline is not feasible, please contact me by email, and we can discuss a suitable alternative.

Don't hesitate to contact me directly with any questions (afarrell@plos.org).

Best regards,

Alison

Alison Farrell, Ph.D.

Senior Editor

PLOS Medicine

afarrell@plos.org

-----------------------------------------------------------

**Response to reviewers in bold after “Response”:**

**Comments in Editorial letter:**

We ask that you address the

- comments of the statistical reviewer (#3)

**Response: We have addressed these below.**

- clarify the rationale for the antibiotic-bacteria combinations chosen

**Response: We have added in a sentence on this as detailed below.**

- consider the potential effects of changing patterns of diagnosis in the elderly.

**Response: We have included a section on bias in sampling and diagnosis in the discussion as detailed below.**

- Please provide an Author Summary with bullet points (the last point should briefly state the study limitations), and

**Response: We have added this to the manuscript.**

- please include an email address for the contact for EARS-Net.

**Response: The email address is** [**data.access@ecdc.europa.eu**](mailto:data.access@ecdc.europa.eu)**. We have added this to the data availability section in the manuscript.**

**Comments from the reviewers:**

**Reviewer #1:** I was invited to review this manuscript, labeled as revision, but I don't recall reviewing an earlier version, and I couldn't find a response to comments made on the initial submission.

**Response: We were asked to address some editorial suggestions after our first submission (further clarification and discussion points). This might explain this labelling as a “revision”.**

The authors have provided a very relevant and important contribution to better predict future trends in AMR infection incidence and burden. It is very obvious from their work that age- and gender incidences should be included. They are - correctly - very careful in their communication about the precision of future incidence rates, and - very wisely - did not include assumptions on attributable mortality or sequelae caused by AMR. As such, this manuscript will set the methodological standard for any future prediction on the burden of AMR.

**Response: Thank you for these comments. We worked hard to ensure that we did not over step the data limitations.**

From a methods point I wondered why the key bug-drug combinations were chosen? Aminopenicillin resistance among E. coli is interesting, but these antibiotics have long been replaced as first-line treatment of E. coli infections (bc of AMR and better alternatives). This could be better explained.

**Response: thank you for this. We have now added in an explanatory sentence in the methods. These were chosen in part due to their wider importance and in part due to their exemplar behaviour in this analysis:**

**Line 175: “We chose these three examples due to these bacteria having a large contributions (>40% [19]) to the aetiology of BSIs, a high number of samples and, after exploration of the trends, variation in the country-level burden estimates”**

Another relevant question is whether we can assume that age in itself does not influence the incidence of BSI. That incidence is associated with age, and a population that gets - on average - older will thus see more episodes of BSI. Yet, BSI relies on a diagnosis made in healthcare settings, and thus a decline in diagnostics applied may reduce the incidence of BSI. At least in some countries (certainly mine) there is a growing tendency to limit medical interventions, including diagnostics, in elderly, especially in subjects with severe comorbidities and needing chronic care. I would not call "less diagnostics" an intervention, but it may well have the same effect on future BSI rates.

**Response: Thank you for highlighting something that we have as an ongoing concern - the importance and interaction of diagnostics with our data surveillance. We have included a further point in the future work section:**

**Line 479: “In particular, surveillance and the analysis of trends must always account for diagnostic coverage. Variations in diagnostic coverage may not only lead to bias in resistance burden (with over sampling of more resistant infections often thought to be likely at lower levels of diagnostic coverage [48]) but potentially an undercounting of BSIs. For example, given a growing tendency to limit medical interventions in some elderly populations (such as those with severe comorbidities needing chronic care) this may lead to an apparent reduction in BSI.”**

Marc Bonten

**Reviewer #2:** Population aging is an important driver of the burden of antimicrobial resistance (AMR) because of the disproportionate impact on the elderly and hospitalized populations. This paper aims to estimates future AMR burden in Europe in light of demographic shifts using data from 12 million plus bloodstream infection (BSI) susceptibility tests.

BSI incidence rates are predicted to increase more in men than women across 7 of 56 the 8 bacteria (P. aeruginosa was the exception) and are projected to increase more dramatically in 57 older age groups (74+ years) but stabilize or even decline in younger age groups. Overall, this is an excellent paper and will add usefully to the literature.

**Response: Many thanks for your review and support for the work.**

**Reviewer #3**: This is a modeling study of the incidence rate of AMR by age and sex until 2025. Overall the analysis is appropriate and builds heavily on prior work by the authors. My minor comments are:

- There are several claims of under-estimation. As the truth is unknown it is hard to call it under-estimation.

**Response: Many thanks for highlighting this - we have gone through and changed this to either clarify that there are “fewer cases estimated” in comparison to a model with age and sex. This has been changed in the abstract and in the results (paragraph starting line 347). We have also altered the times in this paragraph where we refer to the model without age and sex as “incorrect” in agreement with this comment.**

- The models use notation that is never defined (e.g., what do the subscripts i and c refer to).

**Response: The notation is defined - *i* and *c* were given in the paragraph below the equations. To make this clearer we have moved the description of the models to a paragraph above the model equations (starting line 255). We have also clarified the repeated / novel parts of each model and hope this makes things simpler.**

- Is there a reason no interaction between age and sex was included in the models.

**Response: The age:sex interaction term was less important in our previous work than the age^2 term and hence we chose to limit the complexity of the model for this work. Future work could include this term.**

- "Data cleaning and analysis of missing data was reported previously" I would include the missing data component here as it is important to the robustness of the findings.

**Response: We have reported the findings of the missingness analysis in the methods now: line 207: “Missingness in the data appeared completely at random with fewer than 3% of isolates missing both age and sex values”.**

- The limitation section is good and I think appropriately incorporates how uncertain all of this is.

**Response: Thank you for this comment.**

Any attachments provided with reviews can be seen via the following link:

--------------------------------------------------------- ---

General editorial requests:

(Note: not all will apply to your paper, but please check each item carefully)

* We ask every co-author listed on the manuscript to fill in a contributing author statement, making sure to declare all competing interests. If any of the co-authors have not filled in the statement, we will remind them to do so when the paper is revised. If all statements are not completed in a timely fashion this could hold up the re-review process. If new competing interests are declared later in the revision process, this may also hold up the submission. Should there be a problem getting one of your co-authors to fill in a statement we will be in contact. Please do not add or remove authors without first discussing this with the handling editor. You can see our competing interests policy here: [http://journals.plos.org/plosmedicine/s/competing-interests](http://url.uk.m.mimecastprotect.com/s/2zkuCVQRMU8Bp3Zuyi6hEZJsf?domain=journals.plos.org).

**Response: We have done this.**

* Please upload any figures associated with your paper as individual TIF or EPS files with 300dpi resolution at resubmission; please read our figure guidelines for more information on our requirements: [http://journals.plos.org/plosmedicine/s/figures](http://url.uk.m.mimecastprotect.com/s/UBbZCWqVNf83nO4unsyhokPKP?domain=journals.plos.org). While revising your submission, please upload your figure files to the PACE digital diagnostic tool, [https://pacev2.apexcovantage.com/](https://url.uk.m.mimecastprotect.com/s/M7diCX5WOIZAL29hmt8hWKRDe?domain=pacev2.apexcovantage.com). PACE helps ensure that figures meet PLOS requirements. To use PACE, you must first register as a user. Then, login and navigate to the UPLOAD tab, where you will find detailed instructions on how to use the tool. If you encounter any issues or have any questions when using PACE, please email us at PLOSMedicine@plos.org.

**Response: We have done this.**

* Please ensure that the paper adheres to the PLOS Data Availability Policy (see [http://journals.plos.org/plosmedicine/s/data-availability](http://url.uk.m.mimecastprotect.com/s/Rwf5CY58PIXYyM9tZulhxE-aX?domain=journals.plos.org)), which requires that all data underlying the study's findings be provided in a repository or as Supporting Information. For data residing with a third party, authors are required to provide instructions with contact information (web or email address) for obtaining the data. Please note that a study author cannot be the contact person for the data. PLOS journals do not allow statements supported by "data not shown" or "unpublished results." For such statements, authors must provide supporting data or cite public sources that include it.

**Response: The contact information is** [**data.access@ecdc.europa.eu**](mailto:data.access@ecdc.europa.eu)**.**

* We expect all researchers with submissions to PLOS in which author-generated code underpins the findings in the manuscript to make all author-generated code available without restrictions upon publication of the work. In cases where code is central to the manuscript, we may require the code to be made available as a condition of publication. Authors are responsible for ensuring that the code is reusable and well documented. Please make any custom code available, either as part of your data deposition or as a supplementary file. Please add a sentence to your data availability statement regarding any code used in the study, e.g. "The code used in the analysis is available from Github [URL] and archived in Zenodo [DOI link]" Please review our guidelines at [https://journals.plos.org/plosmedicine/s/materials-software-and-code-sharing](https://url.uk.m.mimecastprotect.com/s/Ia96CZ01QiYvWljUXCBhBtkPo?domain=journals.plos.org) and ensure that your code is shared in a way that follows best practice and facilitates reproducibility and reuse. Because Github depositions can

be readily changed or deleted, we encourage you to make a permanent DOI'd copy (e.g. in Zenodo) and provide the URL.

**Response: We have done this, providing both a Git and Zenodo link.**

* Please ensure that the study is reported according to the appropriate guideline, such as TRIPOD if relevant, and include the completed checklist as Supporting Information. When completing the checklist, please use section and paragraph numbers, rather than page numbers. Please add the following statement, or similar, to the Methods: "This study is reported as per [XXXX] guideline (S1 Checklist)."

FORMATTING - GENERAL

* Abstract: Please structure your abstract using the PLOS Medicine headings (Background, Methods and Findings, Conclusions). Please combine the Methods and Findings sections into one section.

**Response: We have done this.**

* At this stage, we ask that you include a short, non-technical Author Summary of your research to make findings accessible to a wide audience that includes both scientists and non-scientists. The Author Summary should immediately follow the Abstract in your revised manuscript. This text is subject to editorial change and should be distinct from the scientific abstract. Ideally each sub-heading should contain 2-3 single sentence, concise bullet points containing the most salient points from your study. In the final bullet point of 'What Do These Findings Mean?', please include the main limitations of the study in non-technical language. Please see our author guidelines for more information: [https://journals.plos.org/plosmedicine/s/revising-your-manuscript#loc-author-summary](https://url.uk.m.mimecastprotect.com/s/_RmHC14LACXgQ3ZtOFDhVl-yw?domain=journals.plos.org).

**Response: We have added this.**

* Please express the main results with 95% CIs as well as p values. When reporting p values please report as p<0.001 and where higher as the exact p value p=0.002, for example. Throughout, suggest reporting statistical information as follows to improve clarity for the reader "22% (95% CI [13%,28%]; p</=)". Please be sure to define all numerical values at first use.

* Please include page numbers and line numbers in the manuscript file. Use continuous line numbers (do not restart the numbering on each page).

* Please cite the reference numbers in square brackets. Citations should precede punctuation.

**Response: we have changed this.**

FIGURES AND TABLES

* Please provide titles and legends for all figures and tables (including those in Supporting Information files).

* Please define all abbreviations used in each figure/table (including those in Supporting Information files).

* Please consider avoiding the use of red and green in order to make your figure more accessible to those with color blindness.

**Response: we have addressed this.**

SUPPLEMENTARY MATERIAL

* Please note that supplementary material will be posted as supplied by the authors. Therefore, please amend it according to the relevant comments outlined here.

* Please cite your Supporting Information as outlined here: [https://journals.plos.org/plosmedicine/s/supporting-information](https://url.uk.m.mimecastprotect.com/s/i0v8C2WMBTQrD1mHLHyh5mpAc?domain=journals.plos.org)

**Response: we have addressed this.**

REFERENCES

* PLOS uses the numbered citation (citation-sequence) method and first six authors, et al.

* Please ensure that journal name abbreviations match those found in the National Center for Biotechnology Information (NCBI) databases ([http://www.ncbi.nlm.nih.gov/nlmcatalog/journals](http://url.uk.m.mimecastprotect.com/s/Sj2yC3wNDcv3rq4HpI5hQFdoY?domain=ncbi.nlm.nih.gov)), and are appropriately formatted and capitalised.

* Where website addresses are cited, please include the complete URL and specify the date of access (e.g. [accessed: 12/06/2023]).

* Please also see [https://journals.plos.org/plosmedicine/s/submission-guidelines#loc-references](https://url.uk.m.mimecastprotect.com/s/ly1cC4LO0CxPwg8t5S2h4cjqX?domain=journals.plos.org) for further details on reference formatting.

**Response: we have addressed this.**

* [FOR POPULATION HEALTH/REGISTRY STUDIES] Please ensure that the study is reported according to the RECORD guideline (available from [https://www.record-statement.org](https://url.uk.m.mimecastprotect.com/s/wUKvC5LPGC18rElHVTQhkZnIN?domain=record-statement.org)) and include the completed checklist as Supporting Information. Please add the following statement, or similar, to the Methods: "This study is reported as per the Reporting of Studies Conducted using Observational Routinely-Collected Data (RECORD) guideline (S1 Checklist)." When completing the checklist, please use section and paragraph numbers, rather than page numbers.

* [FOR POPULATION HEALTH ESTIMATES] Please ensure that the study is reported according to the GATHER statement (available from [https://www.equator-network.org/reporting-guidelines/gather-statement](https://url.uk.m.mimecastprotect.com/s/ifebC69QJSJk4zWHQUph5Dhbj?domain=equator-network.org)) and include the completed checklist as Supporting Information. Please add the following statement, or similar, to the Methods: "This study is reported as per the Guidelines for Accurate and Transparent Health Estimates Reporting (GATHER) statement (S1 Checklist)." When completing the checklist, please use section and paragraph numbers, rather than page numbers.

MODELLING STUDIES

The following list is derived from Geoffrey P Garnett, Simon Cousens, Timothy B Hallett, Richard Steketee, Neff Walker. Mathematical models in the evaluation of health programmes. (2011) Lancet DOI:10.1016/S0140-6736(10)61505-X:

* If pertinent, please provide a diagram that shows the model structure, including how the natural history of the disease is represented, the process and determinants of disease acquisition, and how the putative intervention could affect the system.

* Please provide a complete list of model parameters, including clear and precise descriptions of the meaning of each parameter, together with the values or ranges for each, with justification or the primary source cited and important caveats about the use of these values noted.

* Please provide a clear statement about how the model was fitted to the data, including goodness-of-fit measure, the numerical algorithm used, which parameter varied, constraints imposed on parameter values, and starting conditions.

* For uncertainty analyses, please state the sources of uncertainties quantified and not quantified [can include parameter, data, and model structure].

* Please provide sensitivity analyses to identify which parameter values are most important in the model. Uncertainty estimates seek to derive a range of credible results on the basis of an exploration of the range of reasonable parameter values. The choice of method should be presented and justified.

* Please discuss the scientific rationale for the choice of model structure and identify points where this choice could influence conclusions drawn. Please also describe the strength of the scientific basis underlying the key model assumptions.

* For studies that develop a prediction model or evaluate its performance, please ensure that the study is reported according to the TRIPOD statement ([https://www.equator-network.org/reporting-guidelines/tripod-statement](https://url.uk.m.mimecastprotect.com/s/s9EVC7XRKiYMBlRU1ckho-Gp8?domain=equator-network.org)) and include the completed checklist as Supporting Information. Please add the following statement, or similar, to the Methods: "This study is reported as per the Transparent Reporting of a Multivariable Prediction Model for Individual Prognosis Or Diagnosis (TRIPOD) statement (S1 Checklist)." For studies using machine learning, please use the TRIPOD-AI checklist. When completing the checklist, please use section and paragraph numbers, rather than page numbers.
